# Scientific figures interpreted by ChatGPT: strengths in plot recognition and limits in color perception

**DOI:** 10.1038/s41698-024-00576-z

**Published:** 2024-04-05

**Authors:** Jinge Wang, Qing Ye, Li Liu, Nancy Lan Guo, Gangqing Hu

**Affiliations:** 1 Department of Microbiology, Immunology & Cell Biology, West Virginia University, Morgantown, WV 26506 USA; 2 West Virginia University Cancer Institute, West Virginia University, Morgantown, WV 26506 USA; 3 College of Health Solutions, Arizona State University, Phoenix, AZ 85004 USA; 4 Biodesign Institute, Arizona State University, Tempe, AZ 85281 USA; 5 Department of Occupational and Environmental Health Sciences, West Virginia University, Morgantown, WV 26506 USA

**Keywords:** Computational biology and bioinformatics, Cancer

## Abstract

Emerging studies underscore the promising capabilities of large language model-based chatbots in conducting basic bioinformatics data analyses. The recent feature of accepting image inputs by ChatGPT, also known as GPT-4V(ision), motivated us to explore its efficacy in deciphering bioinformatics scientific figures. Our evaluation with examples in cancer research, including sequencing data analysis, multimodal network-based drug repositioning, and tumor clonal evolution, revealed that ChatGPT can proficiently explain different plot types and apply biological knowledge to enrich interpretations. However, it struggled to provide accurate interpretations when color perception and quantitative analysis of visual elements were involved. Furthermore, while the chatbot can draft figure legends and summarize findings from the figures, stringent proofreading is imperative to ensure the accuracy and reliability of the content.

## Introduction

Known for its remarkable conversational capabilities and extensive knowledge spanning numerous disciplines, large language model (LLM) chatbots like ChatGPT have gathered significant interests in education^1^, research^2^, and clinical practice^3^. In the field of bioinformatics, ChatGPT serves as an instrumental aid for learning basic bioinformatics^4,5^. Several literatures offer recommendations on harnessing the chatbot for more efficient data analysis^4–12^. Further evaluations, centering on biomedical text mining^13,14^, code generation^15,16^, and expertise in genomics/genetics^17–19^, underscore ChatGPT’s potential to facilitate biomedical research. However, present evaluations predominantly focus on text-based inputs. The capability of ChatGPT to interpret alternative inputs, such as scientific figures, a task demanding both skills in imaging pattern recognition and domain-specific knowledge, remains to be evaluated.

In this study, we assessed ChatGPT’s ability to interpret bioinformatics scientific figures using case studies of data analysis frequently used in cancer research. The initial case addressed differential gene expression analysis, highlighting transcriptional alterations in multiple myeloma (MM) when exposed to bone marrow stromal cells (BMSCs)^20^. The subsequent study centered on an integrative approach for drug repositioning in non-small cell lung cancer (NSCLC), leveraging network analysis of the transcription factor ZNF71^21–23^. The third case embarked on clonal evolution underlying the disease progression of MM. The final investigation characterized the epigenetic regulatory landscapes surrounding the *YY1* locus in a human B-lymphoid cancer cell line. Through qualitative and quantitative analyses, we found that the chatbot effectively identified plot types, applied domain knowledge to result in interpretations, and effectively summarized the findings, albeit requiring careful proofreading. Our in-depth assessment revealed that the chatbot is limited in tasks requiring color perception or quantitative analyses, which include counting numbers and inferring positional relationships between visual elements to draw conclusions.

## Results

We designed four case studies in cancer research to assess ChatGPT’s proficiency in deciphering bioinformatics figures (Supplementary Figs. S1–S4). The case background and our interpretation of the figures are listed in Supplementary Methods S1. Prompts used to guide ChatGPT (referred to as GPT-4V hereafter) to interpret the figures are listed in Supplementary Methods S2. GPT-4V’s responses to each case with replicates were recorded, indexed, annotated, and color-coded as true/false at the statement level in Supplementary Notes S1–S4. A summative indexing table was then generated for each case study to document the categorization of statements, their true/false status, and their origins from replicates and figure panels (Supplementary Tables S1–S4; see the “Methods” section for details). Based on the color-coded true/false statements from Supplementary Notes S1–S4, we first conducted a qualitative case-by-case analysis, aiming to identify recurring themes in GPT-4V’s capabilities in reading scientific figures. We then validate the findings via quantitative analyses.

### Qualitative evaluation

#### Case 1—RNA

This case study characterized genes differentially induced by BMSCs in MM cells (see input figure in Supplementary Fig. S1; case background in the “RNA” case study section of Supplementary Methods S1; GPT-4V’s responses with annotations in Supplementary Notes S1). We initially prompted GPT-4V to offer an overview of each panel, suggest enhancements for data presentation, draft a figure legend, and summarize the findings (top three chat histories in Supplementary Notes S1). Across all three evaluations, GPT-4V accurately identified the plot types or offered correct explanations when the plot type was not explicitly stated. Interestingly, all tests failed to interpret color-coding between groups in the volcano plot shown in Supplementary Fig. S1B. It mentioned the two indicated genes, *SOCS3* and *JUNB*, in the same plot but did not delve deeper into their significance. Drafts on summary paragraph presented with no specific error spotted, albeit they could be improved by adding details. Refinements suggested by GPT-4V for data presentation aligned with standard practices.

In the in-depth inquiries, GPT-4V was challenged to estimate specific numbers for indicated genes from the figure and align findings with external knowledge to interpret the figure (bottom three chat histories in Supplementary Notes S1). It correctly estimated the log_2_FC for *SOCS3* but over-estimated it for *JUNB* in the volcano plot from Supplementary Fig. S1B. In the same plot, it consistently failed to identify color for the down-regulated genes. As for domain knowledge, GPT-4V correctly explained SOCS3 as a suppressor of JAK-STAT signaling pathway and JUNB as a proto-oncogene in the context of MM biology. It mentioned the activation of the JAK-STAT signaling pathway by BMSCs and utilized this knowledge to deduce that up-regulated genes were used for the KEGG enrichment analysis shown in Supplementary Figure S1C. The summary paragraphs uniformly detailed the expression up-regulation of *SOCS3* and *JUNB*. However, imperfections from responses to previous inquiries, such as misinterpretations of color-coding, were repeated in the legends and/or summaries.

#### Case 2—ZNF71

This case study focused on a drug-reposition analysis through ZNF71 in NSCLC (see input figure in Supplementary Fig. S2; case background in the “ZNF71” case study section of Supplementary Methods S1; GPT-4V’s responses with annotations in Supplementary Notes S2). In our evaluation, we engaged GPT-4V to provide an overview of the figure and then address specific queries on details related to each panel. The overview accurately captured the plot type from each panel. As for errors, it consistently failed to discern the color representing different patient groups from the K–M plot shown in Supplementary Fig. S2A, though it reliably pinpointed ZNF71 KRAB as an unfavorable prognosis marker. The chatbot estimated an average expression of 4–4.5 for the resistance group marked in Supplementary Fig. S2B, while the reference was 3.3. GPT-4V was further assessed to infer positional relationships among genes in an association network shown in Supplementary Fig. S2C. It successfully identified genes interacting with ZNF71 in all tests but incorrectly spelled the gene name *IKBKB*, with similar incidences observed for *CD27*. The scatter plot in Supplementary Fig. S2D depicted a negative correlation between CD27 expression and EC_50_, which represents the concentration of a drug to kill 50% of the cancer cells. GPT-4V falsely interpreted a lower EC_50_ value to indicate higher drug resistance in replicate three; consequently, it incorrectly associated higher CD27 expression with more resistance to PQ-401 for that replicate.

#### Case 3—Clonal

This case study focused on the analysis of clonal evolution in an MM patient (see input figure in Supplementary Figure S3; case background in the “Clonal” case study section of Supplementary Methods S1; GPT-4V’s responses with annotations in Supplementary Notes S3). When inspecting the overview on figure panels and the summary paragraph, we found that GPT-4V demonstrated a commendable ability to interpret the figure: It accurately identified the correct types of plots, except for the bell plots shown in Supplementary Fig. S3C and D. We further made in-depth inquiries to assess GPT-4V’s quantitative analysis capability, involving tasks like color decoding, sorting clusters by sizes, identifying variant allele frequency (VAF)-changes in clusters, and recognizing leaf nodes from a clonal evolutionary tree. For these tasks, the chatbot encountered difficulties in connecting colors to designated clusters and, at times, referred to non-existing colors. Similarly, it failed to accurately sort clusters by size from Supplementary Fig S3A and often referenced non-existent clusters in Supplementary Fig. S3E and F. Intriguingly, we observed instances where GPT-4V over-interpreted figures. Specifically, it erroneously suggested that node sizes in Supplementary Fig. S3G were proportional to cellular prevalence, despite all nodes being of equal size. The chatbot only sporadically referred to cluster names in the summary paragraphs and could benefit from adding details to improve the content. In conclusion, although GPT-4V showcased a decent interpretation of cancer clonal evolution from the figure, a careful review of the legend and summary is advised, especially when interpreting color-coded elements and when quantitative analysis is required, even at a basic level.

#### Case 4—YY1

This case study examined the epigenetic landscape surrounding the *YY1* locus in a human B-lymphoid cancer line (see input figure in Supplementary Fig. S4; case background in the “YY1” case study section of Supplementary Methods S1; GPT-4V’s responses with annotations in Supplementary Notes S4). In our basal inquiries, we directed GPT-4V to decipher the transcriptional regulation of the *YY1* gene (top three chat histories in Supplementary Notes S4). Without specific guidance, GPT-4V mainly focused on the *YY1* locus. It consistently identified *YY1* as transcriptionally active, basing this on the RNA-Seq signals and associated histone modification patterns. GPT-4V’s interpretation of the promoter-enhancer interactions for *YY1* was generic and lacked details. Notably, GPT-4V offered valuable suggestions to enhance data presentation, such as adopting a colorblind-friendly palette, highlighting the gene of focus (*YY1* in this case), and introducing legends for distinct symbols. The figure legends crafted by the chatbot were concise and captured key elements but ignored the highlighted regulatory domains. While replicates one and two explained the circles shown on the heat map in Supplementary Figure S4, replicate three mistakenly interpreted them as indicating interaction strength rather than hotspots. The summary paragraphs of the findings from the figure, although scientifically not inaccurate, remained at the surface level.

We continued an in-depth evaluation of GPT-4V’s ability to analyze intricate details (bottom three chat histories in Supplementary Notes S4), beginning with a task to identify expressed genes via RNA-Seq signals and associated histone modifications. While the chatbot accurately identified *YY1*, *EVL*, and *WARS* as being expressed, it frequently misinterpreted the expression and histone modification patterns of other genes (Supplementary Table S5). Subsequent tasks involved the interpretation of chromatin domains A1-A3 (active promoters), I1 and I2 (inactive promoters), and E1–E4 (active enhancers). GPT-4V correctly counted the domains in each category and utilized histone modification patterns to recognize the “A” regions as participating in active transcriptional regulation. However, it stumbled in identifying their positional relationships to genes in all replicates. While the “I” regions were generally classified correctly as transcriptionally repressive due to the presence of H3K27me3, replicate two failed to identify this marker, leading to a misleading interpretation. Lastly, for the “E” regions, GPT-4V rightly identified them as enhancers, noting their robust H3K27ac and relatively weak H3K4me3, and deduced their role in transcriptional regulation of target genes via chromatin looping. These outcomes suggest that GPT-4V can leverage external molecular biology knowledge to interpret the transcriptional regulatory role of chromatin domains.

In our next assessment, we aimed to have GPT-4V interpret the chromatin interactions between the *YY1* promoter and three enhancers (denoted by green circles in Supplementary Fig. S4) as well as three promoter regions (denoted by blue circles). Each circle signifies the interaction between two genomic regions, pointed by dashed lines originating from the circle. For instance, chromatin domains “A1” and “A2” are connected to the leftmost blue circle in Supplementary Fig. S4, denoting their physical proximity. GPT-4V could not accurately discern the colors of the two types of circles and reported non-existent purple circles. While manually counting the circles is a straightforward task for human analysts, GPT-4V consistently failed to count the circles in all three replicates. When challenged to identify interacted chromatin regions indicated by the circles, none of the reports from GPT-4V was correct. This underscored GPT-4V’s limitations in not only counting simple visual elements like circles but also deducing relationships between connected visual elements.

Figure legends from the basal assessment consistently overlooked explanations for custom markings on plots, such as rectangles or dashed lines (top three chat histories in Supplementary Notes S4). Summaries lacked sufficient details for concrete conclusions. During the in-depth assessment, GPT-4V incorporated its detailed responses to previous specific questions into the figure legends and result summaries to enrich depth. This approach, however, also led to the inclusion of errors or misleading information from the previous responses, emphasizing the need for rigorous human review to ensure accuracy.

Our qualitative analyses from the four case studies revealed two primary strengths of GPT-4V in figure interpretation. First, it demonstrated competency in identifying and explaining various plot types. Second, it demonstrated competency in leveraging domain knowledge to elucidate or substantiate observations. Regarding limitations, GPT-4V struggled with color perception. Additionally, it faced challenges in discerning the positional relationships between visual elements. Notably, in the “Clonal” and “YY1” case studies, GPT-4V also showed limitations in tasks involving the counting of visual elements. To further measure the significance of these findings, we conducted a comprehensive quantitative analysis in the next section.

### Quantitative evaluation

Six categories emerged from our qualitative assessment to characterize GPT-4V’s responses in scientific figure interpretation: Plot Recognition, Domain Knowledge, Color Perception, Positional Inference, Counts, and Others (see the “Methods” section for definition). The six categories covered 80.3 ± 7.9% of the statements. We devised a summative indexing table for each case study to encapsulate the true/false evaluation of the statements, their categorizations, and the origins of replicates and panels (Supplementary Tables S1–S4).

**Fig. 1:**
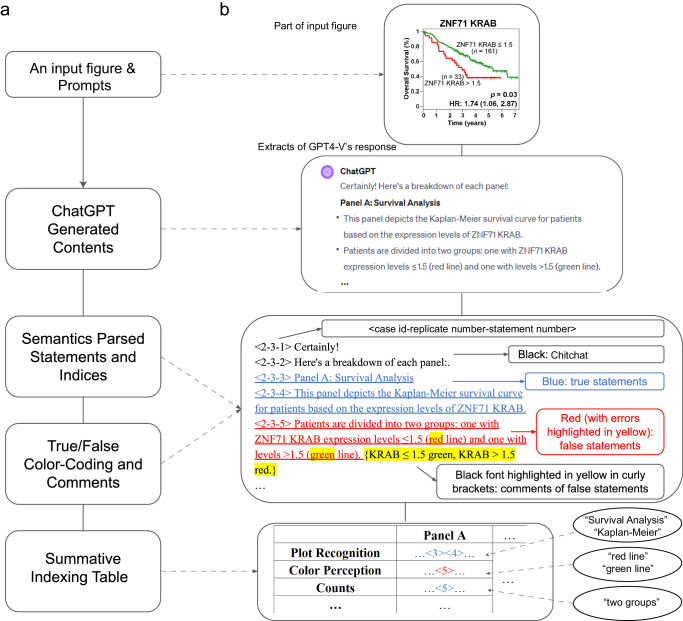
Analytical framework for summarizing GPT-4V’s responses into a summative indexing table for quantitative analysis. **a** Workflow to illustrate the process from the figure and prompt input to GPT-4V, followed by parsing responses into statements and indices, annotating with true/false color codes, culminating in a summative indexing table with categories. **b** Example using GPT-4V’s explanation of plot type in a sub-panel from the “ZNF71” case study to demonstrate statement generation, index creation, true/false annotation, categorization, and allocation in a summative table. Color Coding: Black for non-informative chitchats; blue for correct statements; and red for incorrect statements, with curly-bracketed comments on inaccuracies. Oval text: GPT-4V quotes used for category determination.

As an illustration of this process, Fig. 1a shows an overview, starting from the inputs (figure and prompts) and progressing through parsing GPT-4V’s responses into discrete statements, true/false annotations, categorization, and finally, the compilation of a summative indexing table for downstream comparative analysis. Figure 1b, using extracts from GPT-4V’s explanation of the plot type of a sub-panel from the “ZNF71” case study, demonstrates the workflow from the initial GPT-4V responses through to the indexed and annotated statements and to their allocations in the corresponding summative table. In this example, statements “<2-3-3>” and “<2-3-4>” explained the plot type and were annotated as correct (see the “Methods” section for details on statement indexing). Therefore, the two statement numbers “<3>” and “<4>” were placed in the Plot Recognition category in the summative table and shown in blue. The statement “<2-3-5>” contained correct information on the number of groups such that the statement number (“<5>”) was marked as blue in the Counts category. The statement also contained incorrect information for color coding and thus was marked as red in the Color Perception category.

These summative tables formed the basis for subsequent comparative analyses, which evaluated GPT-4V’s performance within specific categories and across replicates. Note that the basal inquiries were excluded from the analyses, as their corresponding in-depth inquiries elicited more comprehensive responses from GPT-4V.

#### Overall performance

**Fig. 2:**
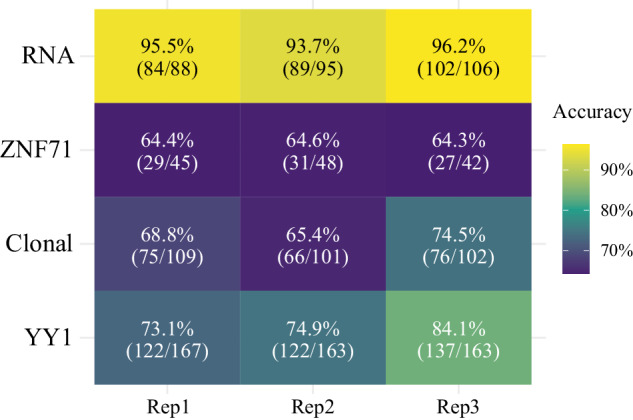
Statement accuracy by replicate and case study. Columns represent each replicate, while rows depict the four case studies: “RNA”, “ZNF71”, “Clonal”, and “YY1”. Accuracy levels indicated by color gradient: higher accuracy in yellow, lower accuracy in deeper shades of purple.

We calculated the percentages of true statements for each case based on the annotations provided in Supplementary Notes S1–S4. As indicated in Fig. 2, the “RNA” case achieved the highest accuracy levels (95.1 ± 1.3%). This may be attributed to the prevalent use of the RNA-Seq technique and the routine nature of the analyses covered in the figure, potentially leading to more effective training of GPT-4V to interpret such figures. Conversely, the overall accuracies from the other three case studies were less impressive, ranging from 64.4 ± 0.1% in the “ZNF71” case to 77.3 ± 5.9% in the “YY1” case (Fig. 2). A noteworthy observation in the “ZNF71” case was the consistent misspelling of “CD27” as “CD271” and “IKBKB” as “IKKBK” (Supplementary Notes S2), which accounted for 40–55% of the false statements. This type of error, only presented in the “ZNF71” case, was a key contributor to its lower accuracy rates: by excluding those statements, the adjusted accuracy increased to 79.2 ± 2.8%.

#### Performance by category

The six categories derived from the qualitative analyses were found in all cases. The summative indexing tables (Supplementary Tables S1–S4) effectively organized statements by category and replicate for each case study. Notably, 23.3% of the statements spanned two categories, and 1.5% intersected three categories. For simplicity, the true/false status of these overlapping statements was independently assessed in each category during their allocations in the summative indexing tables (see Fig. 1b for an example).

**Fig. 3:**
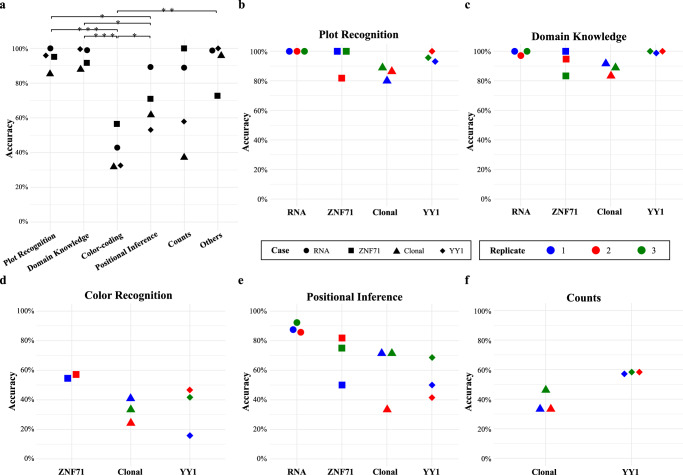
Statement accuracy stratified by category. **a** Accurate statement percentages by category, aggregated by replicates from each of the four case studies: “RNA” (circle), “ZNF71” (square), “Clonal” (triangle), “YY1” (diamond). *P*-values from two-sided *t*-tests indicated as *p < 0.05, **p < 0.01, ***p < 0.001. **b–f** Accurate statement percentages **f**or each replicate across case studies, categorized into plot recognition (**b**), domain knowledge (**c**), color perception (**d**), positional inference (**e**), and counts (**f**). Color coding for replicates: Blue for replicate 1, Red for replicate 2, and Green for replicate 3.

Supplementary Table S6 details the counts of true and total statements for each category and case study combination. This calculation consolidated replicates from each case to ensure a sufficient statement count per category. Statement accuracy for each case study, sorted by category, is visualized in Fig. 3a. These results highlighted GPT-4V’s proficiency in plot type recognition and domain knowledge recall, with accuracies surpassing 85% in all cases (Fig. 3a left two columns). Significantly, these accuracies were markedly higher (*p*-value < 0.05; *t*-test) than those for Color Perception, which consistently showed accuracies below 60% across all cases (Fig. 3a, third column).

Statements in the Positional Inference category describe spatial relationships of visual elements or extract numerical values inferred from coordinate axes. The “RNA” case exhibited a high accuracy of 89.3%. However, its accuracies in the “Clonal” (61.7%) and “YY1” (53.1%) cases were notably lower (Fig. 3a; fourth column). Regarding the Counts category, we observed a bimodal distribution in accuracies: high in the “RNA” (88.9%) and “ZNF71” (100%) cases, while low in the “YY1” (57.9%) and “Clonal” (37.2%) cases (Fig. 3a fifth column). A closer inspection revealed that the counting tasks in the “RNA” and “ZNF71” cases were relatively simple, while the “Clonal” and “YY1” cases represented complex scenarios with multiple groups and/or overlapping with issues in color perception.

Statements under the “Others” category predominantly were suggestions for improving figure presentation or summative sentences of previous statements. In this category, the “RNA”, “Clonal”, and “YY1” cases all demonstrated accuracies as high as 95% or above, while the “ZNF71” case displayed a low accuracy of 72.7% (Fig. 3a; sixth column). This “ZNF71” case had only eleven statements in this category. Moreover, all the inaccurate statements were attributed to the consistent misspelling of “CD27” as “CD271”.

#### Performance by category across replicates

To assess the robustness of performance across replicates, we defined a replicate’s performance in a specific category as unsatisfactory if its accuracy falls below 80%, mirroring a concerning “C” grade in graduate-level evaluation. For each category in every case study, we tabulated the numbers of correct and total statements for each replicate in Supplementary Table S7. To ensure a robust analysis, our focus was on replicates with at least six statements in the relevant category and case studies with at least two such replicates. Figure 3b–f illustrates the accuracies for all case studies sorted by categories, with key observations summarized as follows:

1. Plot Recognition: All replicates showed satisfactory performance for all cases (Fig. 3b).
2. Domain Knowledge: All replicates showed satisfactory performance for all cases (Fig. 3c).
3. Color Perception: All replicates showed unsatisfactory performance for all included cases (Fig. 3d).
4. Positional Inference: Unsatisfactory performance was prevalent across replicates except for those from the “RNA” case (Fig. 3e).
5. Counts: All replicates showed unsatisfactory performance for all included cases (Fig. 3f).

We next explored to what extent GPT-4V may repetitively fail to address a question. To this end, we summarized 52 specific sub-questions by reviewing all incorrect statements (Supplementary Table S8). A response to a sub-question in a replicate was deemed incorrect if it contained one or more inaccurate statements. Our observations revealed the following (Supplementary Table S8): 77.8% of responses to sub-questions in the Color Perception category were consistently incorrect across replicates, followed by Counts (75.0%) and Positional Inference (55%). In contrast, the percentages of consistent, inaccurate responses in the Plot Recognition, Domain Knowledge, and Other categories were lower, at 37.5%, 25.0%, and 20.0%, respectively. This indicates that GPT-4V’s responses in the Color Perception, Counts, and Positional Inference categories, when incorrect, tend to be more persistently incorrect compared to those in the Plot Recognition, Domain Knowledge, and Others categories (*p*-value = 0.01; two-sided *t*-test).

This replicate-based analysis underscored the competency of GPT-4V’s responses in plot recognition and citing domain knowledge. However, it confirmed a significant limitation of GPT-4V in performing tasks for color perception, positional inference, and counts, with consistently poor performance across replicates and cases.

#### Confirmation bias

During the quantitative evaluation of the “YY1” case, we noted a substantial number of instances where GPT-4V applied valid domain knowledge to rationalize flawed observations, a phenomenon known as “confirmation bias”^24^. This occurred in about 5–15% of the statements that cited valid domain knowledge from the in-depth inquiries. Specific instances included statements 22, 25, 29, 49, 53, 56, 86, 218, 220 from replicate one; statements 27, 29, 57, 61, 64, 93, 95, 96, 98, 100, 102, 207, 220, 222 from replicate two; and statements 29, 64, 91, 95 from replicate three (Supplementary Table S4). A notable example was in the interpretation of the “I” regions from replicate two: GPT-4V inaccurately identified them as intronic regions rather than inactive promoters and interpreted them as intronic enhancers (as in the statement “<4-2-95>”), with further explanations about their functions in transcription regulation (“<4-2-96>”), alternative splicing (“<4-2-98>”), and 3D chromatin organization (“<4-2-100>”). This finding underscored the essential role of a human-in-the-loop approach to mitigate potential misinformation from “confirmation bias” and ensure accuracy from GPT-4V’s assistance in figure interpretation.

## Discussion

Data visualization is crucial in conveying results from bioinformatics analyses. LLM chatbots such as ChatGPT have demonstrated an ability to transform natural language prompts into relevant visual representations through coding^25,26^. The newly introduced feature of ChatGPT to take image inputs, namely GPT-4V, offers a promising avenue for identifying patterns within the image, offering interpretations, summarizing findings, and beyond^27^. However, interpreting bioinformatics figures demands specific domain knowledge, an area where the chatbot might not be thoroughly trained. Additionally, chatbots exhibit tendencies toward “hallucinations” when navigating tasks outside their training scope. Considering these factors, a systematic assessment becomes urgent to discern the strengths, weaknesses, and potential pitfalls of utilizing chatbots for interpreting scientific figures.

We carefully designed four use cases to assess various aspects of GPT-4V’s capability in interpreting bioinformatics scientific figures. Our qualitative analysis identified six categories to characterize GPT-4V’s responses in figure interpretation. This categorization laid down a common basis for subsequent quantitative analyses, identifying GPT-4V’s strengths and weaknesses in interpreting scientific figures.

The four case studies encompassed a diverse type of plots, including scatter plots, bar plots, box plots, dot plots, PCA plots, volcano plots, KM survival plots, interaction networks, bell plots, circle-packing plots, tree plots, and multi-track genome browser image. Notably, GPT-4V adeptly recognized these different plot types and elucidated key elements within the plot with an accuracy variation from 85% to 100% across cases. On the other hand, another chatbot, Bard, often struggled to discern the plot type (data not shown). Hence, we focused our assessments on GPT-4V.

Correctly explaining bioinformatics figures requires domain-specific knowledge. Our testing indicated that GPT-4V taps into existing biological knowledge to interpret results, with an accuracy variation from 85% to 100% for citing valid domain knowledge across cases. Even in initial inquiries without detailed instructions in the “YY1” case study, the chatbot referenced the active H3K4me3 and repressive H3K27me3 histone modifications to elucidate the transcriptional status of *YY1*; it further referred to H3K27ac-decorated regions as enhancers and combined with chromatin interaction data to support their transcriptionally regulatory role on *YY1*. In the “RNA” case, when prompted to combine literature for interpretation, the chatbot cited the canonical negative feedback loop between SOCS3 and cytokine signaling to explain *SOCS3*’s transcriptional activation. It then deduced that up-regulated genes were used for pathway enrichment analysis by citing that cytokines activate one of the top hits—the JAK-STAT signaling pathway. However, GPT-4V may need to be explicitly prompted to cross-refer with existing knowledge; otherwise, its feedback remains predominantly centered on the direct content of the illustrations.

The presence of “confirmation bias” in GPT-4V’s responses—where valid domain knowledge is used to justify invalid observations—is of particular concern. Unlike errors in color perception, positional inference, or counting, which can be readily identified from a figure by human eyes, statements from “confirmation bias” are problematic for those without the requisite expertise to detect such biases, making them susceptible to being misled by these plausible responses. This aspect of GPT-4V’s limitation highlights the crucial need for a human-in-the-loop approach to ensure that GPT-4V’s responses are critically evaluated by expert knowledge rather than accepted at face value.

Color differentiation is a fundamental aspect of figure representation. Our analysis revealed that GPT-4V’s weakest performance was in color perception compared to other categories. This trend of poor performance was consistent across replicates, irrespective of the number of colors used—be it two colors in the K–M plot from the “ZNF71” case, three in the volcano plot from the “RNA” case, or four in the scatter plot from the “Clonal” case. Furthermore, the performance did not vary based on the type of colors used. Fortunately, color perception is generally straightforward for humans, making it possible to provide feedback to the chatbot for corrections. However, we found that the effectiveness of the chatbot in correcting these errors heavily relies on the specificity of the feedback, which could be subjective. Consequently, we did not include human feedback in our assessment to avoid artificially inflating the performance metrics.

In bioinformatics figures, manual adjustments are frequently made to enhance the content presentation. This is the case in several plots of our design: the highlighted genes in the volcano plot, the patient group annotations in the K–M plot, and the emphasized chromatin interaction hotspots in the WashU genome browser image. Interpreting these manually edited elements demands quantitative analysis, particularly by assessing their positional relationships to other visual elements. GPT-4V struggled in this challenge as well: It could not accurately determine the coordinates for the indicated genes (*JUNB* and *SOCS3*) in the volcano plot, nor could it correctly associate patient group annotations with their respective color-coded survival curves in the K–M plot. Its attempt to identify genomic regions linked to interaction hotspots was unsuccessful. Our further quantitative analysis reaffirmed GPT-4V’s limited performance in Positional Inference, ranking it as the second most common limitation after color perception. These findings underscored the need for further refinement of GPT-4V in interpreting complex, manually edited elements in bioinformatics figures.

We further evaluated GPT-4V’s proficiency at summarizing illustrations by prompting it to craft figure legends and summative paragraphs. Major issues included inaccuracies in listing replicate numbers, misidentification of colors in the legend, and omissions of plot markers. GPT-4V’s summary paragraphs were lack of details. During the in-depth assessments, while the chatbot did include details, it occasionally reflected errors made in early responses. This was particularly evident in the “YY1” case study: the summary cited marked regulatory regions from the plot but mischaracterized their interacting relationship with the YY1 promoter or provided misleading interpretations for individual regions. Thus, while GPT-4V is equipped to draft figure legends and summaries, rigorous manual proofreading and detailed revision are indispensable to ensure accuracy and prevent the dissemination of misleading information.

The present study has its limitations. Our evaluations were based on four use cases, with further extrapolation to other topics necessitating collaboration with experts in relevant domains. Our qualitative and quantitative evaluations, however, do illuminate consistent themes regarding GPT-4V’s performance in interpreting bioinformatics figures: While the chatbot demonstrated proficiency in explaining various plots and citing domain knowledge, it struggled with color perception and quantitative analysis of visual elements. Additionally, while it can draft figure legends and offer summaries, human proofreading is paramount to mitigate “confirmation bias” and ensure accuracy and depth in interpretations. It’s also crucial to acknowledge that our primary aim was to establish a performance baseline for GPT-4V, focusing on its inherent strengths and limitations without resorting to external enhancements such as providing feedback. Prompt engineering^27–29^ also holds significant promise in enhancing performance. However, most existing prompt engineering techniques are tailored for text inputs. Their effectiveness for image inputs, especially scientific figures, is not yet well-established. We believe that this gap in knowledge presents an exciting opportunity for future research.

In conclusion, our assessments revealed that ChatGPT exhibited significant promise in deciphering bioinformatics scientific figures. Nevertheless, it faces challenges, especially in interpreting colors and conducting quantitative analyses. To harness ChatGPT’s full potential in this direction, human oversight is indispensable for the validation and refinement of its outputs. Our analysis also underscored the need for similar evaluations when extending the image-reading capability of the chatbot to other critical domains such as medical diagnosis. As we progress, infusing chatbots with domain-specific expertise, human feedback, and image-input-specific prompts will be pivotal in enhancing the quality of their responses.

## Methods

### Source of data and procedure of figure generation

Gene expression data used for the “RNA” case study was sourced from our previous work^20^. This case specifically contrasted the RPMI8226, a multiple myeloma (MM) cell line, in trans-well coculture with BMSCs (T) against its monoculture (M). Gene expression values, expressed as Reads Per Kilobase of exon per Million reads mapped (log_2_)^30^, were used to generate a principal component analysis (PCA) plot. Fold change (FC) in expression (T/M; log_2_) and false discovery rate (FDR; ‒log_10_), which measures the significance of differential expression, were utilized to craft a volcano plot. Additionally, a dot or bubble plot illustrating hits in pathway enrichment analysis for up-regulated genes (log_2_FC > 1 and FDR < 0.05) was produced using ShinyGO^31^.

For the “ZNF71” case study, gene expression data with survival information from 194 patients of NSCLC^32^ were used to generate Kaplan–Meier (K–M) curves. We combined gene expression data and PRISM drug screening data of NSCLC cell lines from the DepMap data portal^33^ to relate docetaxel-sensitivity with ZNF71 KRAB expression and to examine the correlation between drug response and CD27 expression. Gene expression of tumors and cell lines were expressed as transcripts per million (TPM). We applied the Boolean implication network algorithm^34^ to construct gene association networks within the context of tumors and normal tissues adjacent to the tumors (NATs) using Xu’s lung adenocarcinoma (LUAD) cohort^35^.

For the “Clonal” case study, somatic mutations in a pair of primary and recurrent tumors from an MM patient (ID: 1201) in the MMRF-CoMMpass study were sourced from the GDC data portal^36^. Clonal and subclonal mutations were identified using the MAGOS method^37^. The relative prevalence of (sub)clones and their evolutionary relationships were inferred using the ClonEvol method^38^.

For the “YY1” case study, a screenshot for gene expression, genomic distributions of histone modifications, and chromatin–chromatin interactions in the genomic region encompassing *YY1* in GM12878 was sourced from the WashU Epigenome Browser^39^. Specifically, the Chromatin immunoprecipitation followed by sequencing (ChIP-seq) data for histone modifications H3K27me3, H3K4me3, and H3K27ac, along with strand-specific RNA sequencing (RNA-Seq) for gene expression, were loaded from the Encyclopedia of DNA Elements (ENCODE) data hub. The Proximity Ligation-Assisted ChIP-Seq (PLAC-seq) data for chromatin–chromatin interactions was loaded from the 4D Nucleome (4DN) Network. Regions denoting active and inactive chromatin domains, as well as key areas of chromatin–chromatin interactions, were annotated manually.

Input figures to GPT-4V from the above four case studies were listed in Supplementary Figs. S1–S4. Legends of the figures were drafted by the authors. Additional case background and our interpretations of the figures were provided in Supplementary Methods S1 as references.

### Prompts for ChatGPT

We instructed GPT-4V to function as a bioinformatics expert for each case study, introducing the research question associated with each figure at a high level to offer context. GPT-4V’s interpretive capabilities may be demonstrated under two assessment models. In the basal model, GPT-4V operated with minimal guidance to provide an overview of a figure, while in the in-depth model, it was guided through a sequence of questions addressing details. The questions were prompted to the chatbot one by one, which yielded more detailed responses compared to prompting all questions at once. At the conclusion of each evaluation, GPT-4V was optionally tasked to draft a figure legend and a summary paragraph for the figure. Prompts used for each case study were detailed in Supplementary Methods S2. We repeated each assessment three times using the web interface of ChatGPT-4 Plus (version dated Sep 25, 2023). All experiments were conducted under the default settings of ChatGPT-4 Plus.

### Summative indexing tables for quantitative assessment

We compiled chat histories from each case study into a unified document (Supplementary Notes S1–S4). This process began with the segmentation of GPT-4V’s responses into discrete statements using Spacy v3.6.1^40^. Subsequently, these statements were indexed in a sequential manner to facilitate easy referencing. The indexing format adopted is <Case-Replicate-Statement>. In this format, “Case” represents the case study number, with 1 for “RNA”, 2 for “ZNF71”, 3 for “Clonal”, and 4 for “YY1”. The term “Replicate” indicates the replicate number, which can be either 1, 2, or 3. “Statement” corresponds to the statement’s sequential position within a specific replicate of a case study. For example, the third statement (3) in the second replicate (2) of the “RNA” case study (1) is indexed as <1-2-3>. Furthermore, a letter “B” is appended to the “Case” slot if the chat history originates from prompts associated with the basal assessment model.

Each statement from a chat history was then assigned a color code based on evaluation outcome: blue signifies true statements, red indicates false statements (with specific errors highlighted in yellow), and black denotes statements that were excluded from the assessment, such as chitchat (small talk or gossip). Additional explanations were provided following the statements classified as false to offer clarity on the nature of their inaccuracies.

To facilitate quantitative assessment, we summarized six categories to characterize statements from GPT-4V:

1. Plot recognition: Statements that identify and/or explain plot types.
2. Domain knowledge: Statements that utilize external knowledge to interpret results.
3. Color perception: Statements that perceive colors of visual elements.
4. Positional inference: Statements that analyze the positional relationships between visual elements.
5. Counts: Statements that count the number of visual elements.
6. Others: Statements that do not fall into the above five categories.

A summative indexing table was then generated for each case study (Supplementary Tables S1–S4). This table documents the categorization of statements, their true/false status, and their origins from replicates and panels. In instances where a statement falls into multiple categories, it is assigned a true/false status for each category independently.

### Reporting summary

Further information on research design is available in the Nature Research Reporting Summary linked to this article.

### Supplementary information

REPORTING SUMMARY

Supplementary Information File

## Data Availability

Prompts and GPT-4V transcripts are in Supplementary Methods S2 and Supplementary Notes S1–S4 of the manuscript.
