# Supplementary material for: Scientific figures interpreted by ChatGPT: strengths in plot recognition and limits in color perception
Source: NPJ Precis Oncol. 2024 Apr 5;8:84. doi: 10.1038/s41698-024-00576-z (PMC10997760; doi:10.1038/s41698-024-00576-z)
Supplement: Supplementary file 2 — Supplementary Information File [file 41698_2024_576_MOESM2_ESM.pdf]

SUPPLEMENTARY FILES

Contents

SUPPLEMENTARY FIGURES 2
Supplementary Figure S1 2
Supplementary Figure S2 3
Supplementary Figure S3 4
Supplementary Figure S4 5

SUPPLEMENTARY TABLES 6
Supplementary Table S1 6
Supplementary Table S2 7
Supplementary Table S3 8
Supplementary Table S4 9
Supplementary Table S5 10
Supplementary Table S6 11
Supplementary Table S7 12
Supplementary Table S8 13

SUPPLEMENTARY METHODS 18
Supplementary Methods S1 18
Supplementary Methods S2 20

SUPPLEMENTARY NOTES 25
Supplementary Notes S1 25
Supplementary Notes S2 54
Supplementary Notes S3 64
Supplementary Notes S4 82

SUPPLEMENTARY FIGURES

Supplementary Figure S1

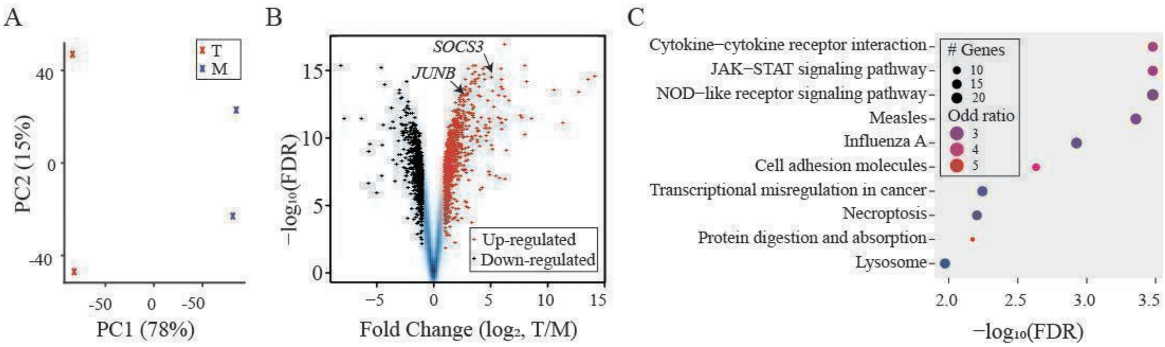

Supplementary Figure S1. Input figure for the "RNA" case study and figure legend drafted by the authors. (A) PCA analysis showing two groups of samples: "T" in red and "M" in blue. "T": MM cells in transwell coculture with BMSCs. "M": MM cells in monoculture. (B) A volcano plot for FCs and FDRs from differentially expressed gene analysis with up-regulated genes in red and down-regulated genes in black. SOCS3 and JUNB indicated by arrowheads. FC: fold change; FDR: false discovery rate. (C) Dot plot summarizing the top ten hits from KEGG pathway enrichment analysis applied to the up-regulated genes.

Supplementary Figure S2

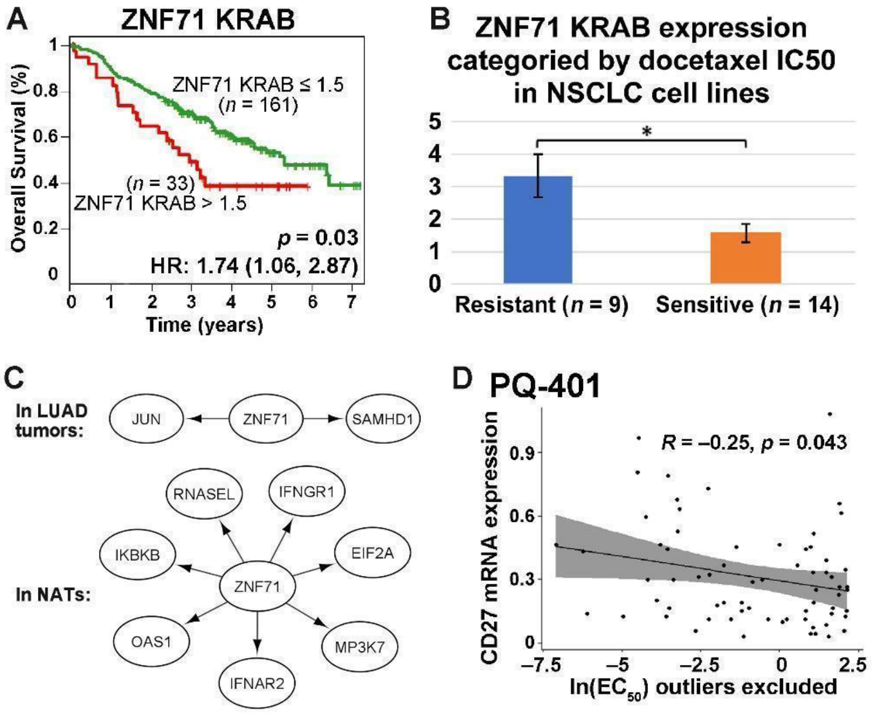

Supplementary Figure S2. Input figure for the "ZNF71" case study and figure legend drafted by the authors.

(A) Kaplan–Meier survival plot for NSCLC patients (GSE81089) sorted by ZNF71 KRAB isoform expression. Red: patients with a higher ZNF71 KRAB expression (TPM > 1.5); Green: others; HR: hazard ratio; *p*: *p*-value. (B) A bar chart for the expression of ZNF71 KRAB isoform among NSCLC cell lines sorted by docetaxel sensitivity. NSCLC: non-small cell lung cancer. IC50: concentration of an inhibitor when the response is decreased by half. (C) Gene association network of ZNF71 and IIIR genes in LUAD tumors and NATs. LUAD: lung adenocarcinoma; NATs: normal tissues adjacent to the tumors. IIIR: intracellular innate immune response. (D) Scatter plot for CD27 mRNA expression level and drug response to PQ-401 (EC_{50}) among NSCLS cell lines. EC_{50}: The concentration of a drug to kill 50% of the cancer cells. R: Pearson Efficiency.

Supplementary Figure S3

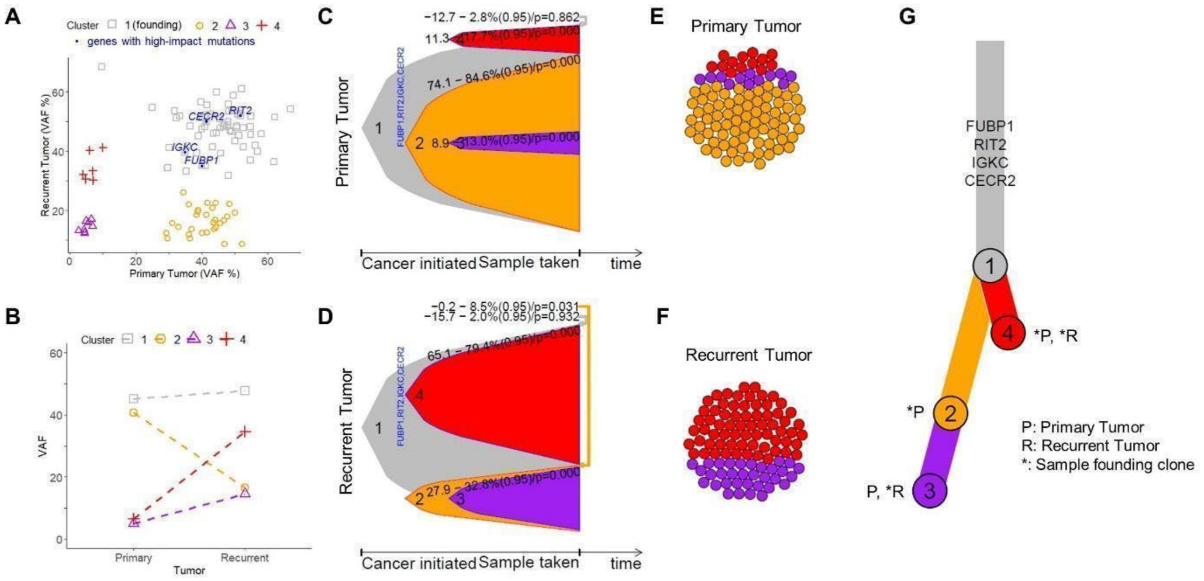

Supplementary Figure S3. Input figure for the "Clonal" case study and figure legend drafted by the authors.

(A) Scatter plot for VAF% contrasting recurrent tumor (y-axis) to primary tumor (x-axis). Each dot is a gene and genes with significant impact are indicated. Primary and recurrent tumors sourced from the same patient. VAF: Variant Allele Frequency. (B) Connected dot plot illustrating the changes of mean VAF of each cluster from the primary tumor to the recurrent. (C) Bell plot illustrating the relative prevalence of each (sub)clone as a function of cancer progression for primary tumor. Each "bell" symbolizes a (sub)clone, with the area of the bell being proportional to cellular prevalence within the tumor. A 95% confidence interval, along with a p-value testing for non-zero prevalence, is displayed for each (sub)clone. (D) Similar to panel C but for the recurrent tumor. (E) Circle packing plots illustrating cellular composition of the primary tumor, exampled with a total of 100 cells. (F) Similar to panel E but for the recurrent tumor. (G) A branch-based tree illustrating the relational and seeding patterns of the (sub)clones between the primary and recurrent samples. Indicated are genes facilitating the growth of the founding clone.

Supplementary Figure S4

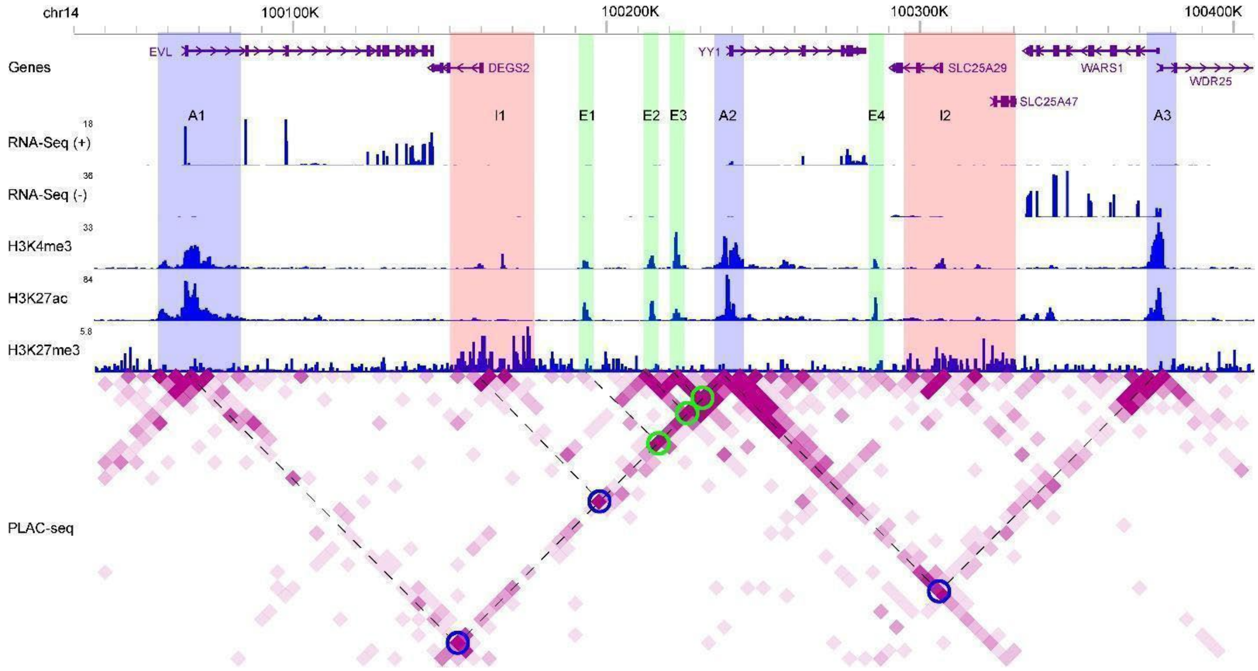

Supplementary Figure S4. Input figure for the "YY1" case study and figure legend drafted by the authors.

WashU epigenome browser tracks illustrating distributions of strand-specific RNA-Seq (RNA sequencing) reads, ChIP-seq (Chromatin immunoprecipitation followed by sequencing) reads for H3K4me3 (trimethylation of histone H3 at lysine 4), H3K27ac (histone H3 lysine 27 acetylation), and H3K27me3 (trimethylation of histone H3 lysine 27), as well as PLAC-seq (Proximity Ligation-Assisted ChIP-Seq) reads for chromatin-chromatin interactions. Blue rectangles (A1 to A3): active promoter regions; Pink rectangles (I1 and I2): inactive promoter regions; Green rectangles (E1 to E4): active enhancer regions; Blue circles: promoter regions (A1, I1, and A3) interacting with YY1; Green circles: enhancer regions (E1 to E3) interacting with YY1. Dashed lines shared with one circle on the heatmap: two interacting genome regions. Degree of purpleness on the heatmap indicting interaction strength.

SUPPLEMENTARY TABLES

Supplementary Table S1

Supplementary Table S1. Summative indexing table for the “RNA” case study.
Colored Background for Replicates: Replicate 1: Yellow, Replicate 2: Green, Replicate 3: Gray. **Acc. %:** Cross-panel accuracy of statements in each category by replicates.
<Indices>: The indices correspond to the statement numbers from the statement indices from **Supplementary Notes S1**; the table summarizes statements from the in-depth assessment. Blue: statement content is correct in the specified category; Red: statement content is incorrect in the specified category.

Category	Panel A	Panel B	Panel C	Acc. %
**Plot Recognition**	<3><4><5><6><8><26><70><100><101><111>	<10><11><12><16><34><77><102><103><104><112>	<17><18><19><20><21><22><55><84><106><107><108><113>	100% (32/32)
	<3><5><6><80><113><129>	<11><13><14><15><85><115><116><130>	<19><21><22><23><24><64><66><89><119><120><121><122><131>	100% (27/27)
	<3><4><5><6><9><92><125><126><137>	<11><13><14><15><16><43><96><128><129>	<21><22><23><24><25><26><27><28><72><73><75><76><105><133><134>	100% (33/33)
**Positional Inference**	<9>	<36><38><40><112>	<23><53><54>	87.5% (7/8)
	<10>	<39><41><43><130>	<60><62>	85.74% (6/7)
	<10><137><138>	<39><41><44><52><59><62>	<66><67><69><70>	92.31% (12/13)
**Color Perception**	<100>	<13><14>	NA	66.67% (2/3)
	<9><113>	<17><117>	NA	0% (0/4)
	<7><8><127>	<17><18><19><130>	NA	57.14% (4/7)
**Counts**	<7><109>	NA	NA	50% (1/2)
	<8><10><129>	<18>	NA	100% (4/4)
	<10>	<20><100>	NA	100% (3/3)
**Domain Knowledge**	<25><27><28><29><30><31><32>	<43><44><45><47><48><49><50>	<23><58><59><60><62><63><64><65><66><113>	100% (24/24)
	<30><32><34><35><36><114><129>	<45><46><47><49><52><53><54><55><56>	<26><27><65><69><70><72><73><74><75><76><77><78><120><121><124><125><126><132>	97.06% (33/34)
	<9><30><32><33><34><35><36><37>	<46><47><49><50><52><53><54><56><57><59><61><62>	<21><24><78><79><80><82><83><85><86><87><88><89><132><139><140><141><142>	100% (37/37)
**All Others**	<15><24><26><51><71><72><74><76><79><81><83><85><86><88><90><92><93><95><96><97><99><105><110><114>			95.83% (23/24)
	<26><27><28><29><44><57><81><83><84><86><88><90><92><94><95><96><98><100><102><105><107><108><109><112><114><118><124><125><126><127><128><132><131><133>			100% (34/34)
	<29><46><60><63><93><95><97><98><102><104><107><108><110><113><115><117><119><120><123><131><135><136>			100% (22/22)

Supplementary Table S2

Supplementary Table S2. Summative indexing table for the "ZNF71" case study

Colored Background for Replicates: Replicate 1: Yellow, Replicate 2: Green, Replicate 3: Gray. **Acc. %:** Cross-panel accuracy of statements in each category by replicates. **<Indices>**: The indices correspond to the statement numbers from the statement indices from **Supplementary Notes S2**. Blue: statement content is correct in the specified category; Red: statement content is incorrect in the specified category.

Category	Panel A	Panel B	Panel C	Panel D	Acc. %
**Plot Recognition**	<3><4><24>	<9><10><30><31><32>	<14><15><37>	<18><19><21><41>	100% (15/15)
	<3><4><31>	<13><36>	<21><24><41>	<25><26><27>	81.82% (9/11)
	<3><4><6>	<10><11><12>	<15><16><17><19><42>	<20><21><23><24>	100% (15/15)
**Positional Inference**	<27>	<33><34><35>	<16><17><38><39>	NA	50% (4/8)
	<10><32><33>	<19><37><38><39>	<22><23><42><43>	NA	81.82% (9/11)
	<7><28><29>	<13><35><36><37><38>	<17><18><40><41>	NA	75% (9/12)
**Color Perception**	<5><6><25><26><27>	<11><12><31><32><33><34>	NA	NA	54.55% (6/11)
	<6><8><32>	<15><17><37><38>	NA	NA	57.14% (4/7)
	<5><7><29>	<12><35>	NA	NA	60% (3/5)
**Counts**	<5>	<11>	NA	NA	100% (2/2)
	<5><32>	<14>	NA	NA	100% (3/3)
	<5><8>	<12>	NA	NA	100% (3/3)
**Domain Knowledge**	<7><8><28><29>	<13><15>	<21><38><39><40>	<20><22><43><45><46><47><48>	100% (17/17)
	<10><11><12><33><34><35>	<20><40>	<21><43>	<28><29><45><46><47><48><49><50>	94.74% (18/19)
	<7><8><9><26><30><31><32>	<10><11><14><33><37><38>	<17><18><40><41><42>	<24><44><45><46><47><48>	83.33% (20/24)
**All Others**	<23><36><42>				66.67% (2/3)
	<7><9><16><18><30><44>				83.33% (5/6)
	<22><25>				50% (1/2)

Supplementary Table S3

Supplementary Table S3. Summative indexing table for the “Clonal” case study

Colored Background for Replicates: Replicate 1: Yellow, Replicate 2: Green, Replicate 3: Gray. **Acc. %:** Cross-panel accuracy of statements in each category by replicates. **<Indices>**: The indices correspond to the statement numbers from the statement indices from **Supplementary Notes S3**. Blue: statement content is correct in the specified category; Red: statement content is incorrect in the specified category.

Category	Panel A	Panel B	Panel C&D	Panel E&F	Panel G	Acc. %
**Plot Recognition**	<3><4><5><6><109><110><125>	<8><10><11><12><112>	<13><14><15><16><48><114><115><127>	<19><20><21><22><116><117><128>	<23><25><26><27><29><67><71><73><118><119><121><122><129>	80% (32/40)
	<3><4><5><6><7><107><108><109><127>	<8><10><11><12><110><111><112><127>	<14><15><16><17><52><113><114><115><128>	<20><21><22><23><56><92><119><120><121><129>	<25><26><27><28><69><122><123><130>	86.36% (38/44)
	<3><4><5><6><97><107><123>	<7><8><9><41><97><109>	<11><12><13><14><15><17><18><19><46><111><112><113><114>	<20><22><23><25><27><28><51><115><116><117><126>	<29><30><31><32><66><91><118><127>	88.89% (40/45)
**Positional Inference**	NA	<42><43><44><45><46><47><126>	<49><50><51><52><53><54><127>	NA	<69><70><74><75><76><77><120>	71.43% (15/21)
	NA	<46><47><49><50>	<53><54><55>	NA	<71><73><74><75><76>	33.33% (4/12)
	NA	<41><43><44><45><124>	<47><48><49><50><125>	NA	<68><71><72><119>	71.43% (10/14)
**Color Perception**	<32><34><35><36><37><111>	<42><44><113>	<49><51>	<56><57><58><59><60><61><62><63>	<69><74><84>	40.91% (9/22)
	<32><35><38><39><40><42><44><124>	<49><50> <51><124>	<17><53><54><124>	<58><59><60><61><63><64><65><66><67><68><124>	<71><73><74><75><123><124>	24.24% (8/33)
	<34><35><36><37><38><108><121>	<43><44><110><121>	<47><48><49><50><121>	<53><54><56><58><60><61><63><64><121>	<68><71><72><119><121>	33.33% (10/30)
**Counts**	<34><35><36><37>	NA	NA	<56><57><58><59><60><61><62><63><65><66><128>	NA	33.33% (5/15)
	<38><39><40><43><44>	NA	NA	<58><59><60><61><63><64><65><66><67><68>	NA	33.33% (5/15)
	<35><36><37><38>	NA	NA	<53><54><56><58><60><61><63><64><126>	NA	46.15% (6/13)
**Domain Knowledge**	<7><38><109>	<40>	<17>	<22>	<28><30><67><71><123><129>	91.67% (11/12)
	<7><109><125>	<125>	<15><18><117>	NA	<25><29><30><69><125>	83.33% (10/12)
	<4><123>	<10>	<16>	<65>	<66><69><73><127>	88.89% (8/9)
**All Others**	<31><33><55><68><72><79><82><83><86><87><89><90><92><93><95><96><98><99><101><103><105><106><107><124>					91.67% (22/24)
	<24><37><52><56><80><82><83><85><86><87><89><90><93><96><98><99><101><103><104><105><116><126><131>					100% (23/23)
	<33><40><42><70><75><77><78><80><81><83><84><86><87><89><90><92><95><101><103><104><105><106><120><122>					95.83% (23/24)

Supplementary Table S4

Supplementary Table S4. Summative indexing table for the "YY1" case study

Colored Background for Replicates: Replicate 1: Yellow, Replicate 2: Green, Replicate 3: Gray. **Acc. %:** Cross-panel accuracy of statements in each category by replicates. **<Indices>**: The indices correspond to the statement numbers from the statement indices from **Supplementary Notes S4**; the table summarizes statements from the in-depth assessment. Blue: statement content is correct in the specified category; Red: statement content is incorrect in the specified category. Shaded: Domain knowledge, which by itself is correct, is used to interpret a false claim ("confirmation bias").

Category	List of sentences sorted by category for each replicate	Acc. %
**Plot Recognition**	<2><5><6><8><10><11><12><121><123><124><127><128><129><130><135><198><199><200><201><202><204><205><206><207><208><209><211><212><216>	93.1% (27/29)
	<2><5><7><9><12><13><14><20><21><22><24><122><126><127><128><133><193><194><195><203><204>	100% (21/21)
	<2><6><9><10><12><14><15><16><20><23><24><25><26><124><126><127><128><130><131><196><197><198><209>	95.65% (22/23)
**Positional Inference**	<3><11><12><21><22><23><25><27><29><31><47><48><49><50><51><52><53><54><56><57><58><60><62><63><77><78><79><81><82><83><85><86><87><91><95><96><100><107><109><110><132><134><135><137><140><143><149><151><153><155><156><157><213><217><218><219><220><221>	50% (29/58)
	<3><15><16><27><29><31><33><38><55><56><57><59><61><64><65><66><76><77><79><80><81><89><91><92><93><95><102><105><107><108><110><118><130><132><147><149><151><152><153><154><157><159><160><161><206><208><211><212><218><219><220><221><222>	41.51% (22/53)
	<4><15><16><17><29><30><31><32><38><54><55><57><59><60><61><62><64><65><80><81><82><85><86><87><91><93><95><100><101><102><111><113><114><132><135><137><139><151><152><153><154><155><156><203><204><205><207><214><215><216><217>	68.63% (35/51)
**Color Perception**	<139><141><142><143><144><145><146><149><151><153><155><158><159><160><207><208><209><213><214>	15.79% (3/19)
	<136><137><138><139><140><141><144><145><152><165><206><207><208><210><212>	46.67% (7/15)
	<143><144><145><146><147><148><157><158><159><160><206><208>	41.67% (5/12)
**Counts**	<67><69><71><72><73><74><140><141><143><144><145><146><219><221>	57.14% (8/14)
	<68><69><70><71><72><73><79><137><139><140><141><145>	58.33% (7/12)
	<69><71><73><74><75><76><85><144><146><147><148><157>	58.33% (7/12)
**Domain Knowledge**	<7><9><17><18><19><21><22><23><25><27><29><32><33><34><38><39><40><41><42><43><44><45><47><48><49><53><56><61><77><78><79><85><86><87><88><89><90><92><95><96><98><99><100><101><102><103><104><108><110><112><113><114><115><116><117><118><119><120><125><133><134><135><137><157><168><198><199><200><201><202><204><205><206><207><208><209><217><218><219><220><221><222>	98.78% (81/82)
	<7><9><10><12><13><14><17><20><21><22><23><27><29><31><37><41><42><43><44><45><46><47><48><49><50><51><52><55><56><57><59><61><63><64><76><77><79><80><81><83><84><85><86><91><92><93><95><96><98><100><102><105><107><109><110><112><113><115><116><117><118><119><123><124><131><132><134><193><194><195><196><197><198><199><200><201><202><206><207><208><213><218><219><220><221><222>	100% (86/86)
	<9><12><14><15><16><20><23><24><25><26><27><29><30><32><34><35><36><37><38><40><41><42><43><44><45><46><47><48><49><50><51><54><55><57><59><62><64><85><86><87><91><93><95><96><100><101><102><104><105><106><111><112><113><114><116><117><118><119><120><122><124><136><138><139><140><197><198><199><200><201><213><214><215><216><217><218>	100% (76/76)
**All Others**	<13><35><66><68><70><136><165><166><167><168><170><171><173><175><177><178><180><181><184><186><187><190><192><193><194><203><215><216>	100% (28/28)
	<6><8><11><18><32><35><36><39><101><146><163><164><167><168><170><171><173><174><176><177><180><181><182><184><185><186><190><215><217><223>	100% (30/30)
	<18><21><28><53><56><58><63><68><70><72><79><84><89><103><107><109><115><121><162><165><167><168><170><171><172><174><176><178><180><183><184><186><187><191><195><211>	100% (36/36)

Supplementary Table S5

Supplementary Table S5. Summary of GPT-4V's identification of expressed genes and the associated histone modifications. "+": expressed or presence of the indicated histone marker; "-": not expressed or absence of the indicated histone marker; "na": not explicitly identified by ChatGPT; "Manual": manual annotation; "Eval 1/2/3": the three times of assessments; Red rectangle: GPT-4V's identification conflicting with manual annotation.

		EVL	*DEGS2*	*YY1*	*SLC25A29*	*SLC25A47*	*WARS1*	*WDR25*
RNA-Seq signal	Manual	+	-	+	-	-	+	-
	Eval 1	+	+	+	+	+	+	-
	Eval 2	+	+	+	+	+	+	+
	Eval 3	+	-	+	-	-	+	-
H3K4me3	Manual	+	+	+	+	-	+	+
	Eval 1	+	+	+	+	+	+	-
	Eval 2	+	+	+	na	na	+	na
	Eval 3	+	na	+	na	na	+	na
H3K27ac	Manual	+	-	+	-	-	+	+
	Eval 1	+	+	+	+	+	+	-
	Eval 2	+	+	+	+	+	+	+
	Eval 3	+	na	+	na	na	+	+
H3K27me3	Manual	-	+	-	+	+	-	-
	Eval 1	-	-	-	-	-	na	+
	Eval 2	na	na	na	na	na	na	na
	Eval 3	-	+	na	+	+	na	+

Supplementary Table S6

Supplementary Table S6. Counts of accurate and total statements by category, aggregated across all replicates for each case study.
* Numbers in parentheses indicate accuracy percentage, highlighted in red for values less than 80%.

Category	RNA	ZNF71	Clonal	YY1
**Plot Recognition**	92/92 (100.0%)	39/41 (95.1%)	110/129 (85.3%)	70/73 (95.9%)
**Domain Knowledge**	94/95 (99.0%)	55/60 (91.7%)	29/33 (87.9%)	243/244 (99.6%)
**Color Perception**	6/14 (42.9%) *	13/23 (56.5%)	27/85 (31.8%)	15/46 (32.6%)
**Positional Inference**	25/28 (89.3%)	22/31 (71.0%)	29/47 (61.7%)	86/162 (53.1%)
**Counts**	8/9 (88.9%)	8/8 (100.0%)	16/43 (37.2%)	22/38 (57.9%)
**All Others**	80/81 (98.8%)	8/11 (72.7%)	68/71 (95.8%)	94/94 (100.0%)

Supplementary Table S7

Supplementary Table S7. Counts of accurate and total statements by category on replicate basis for each case study.
*"NA" denotes instances where accuracy was not calculated due to a total statement count of less than six. **Numbers in parentheses indicate accuracy percentage, highlighted in red for values less than 80%.

Category		RNA	ZNF71	Clonal	YY1
**Plot Recognition**	Rep1	32/32 (100%)	15/15 (100%)	32/40 (80%)	27/29 (93.1%)
	Rep2	27/27 (100%)	9/11 (81.8%)	38/44 (86.4%)	21/21 (100%)
	Rep3	33/33 (100%)	15/15 (100%)	40/45 (88.9%)	22/23 (95.7%)
**Domain Knowledge**	Rep1	24/24 (100%)	17/17 (100%)	11/12 (91.7%)	81/82 (98.8%)
	Rep2	33/34 (97.1%)	18/19 (94.7%)	10/12 (83.3%)	86/86 (100%)
	Rep3	37/37 (100%)	20/24 (83.3%)	8/9 (88.9%)	76/76 (100%)
**Color Perception**	Rep1	2/3 (NA) *	6/11 (54.6%) **	9/22 (40.9%)	3/19 (15.8%)
	Rep2	0/4 (NA)	4/7 (57.1%)	8/33 (24.2%)	7/15 (46.7%)
	Rep3	4/7 (57.1%)	3/5 (NA)	10/30 (33.3%)	5/12 (41.7%)
**Positional Inference**	Rep1	7/8 (87.5%)	4/8 (50.0%)	15/21 (71.4%)	29/58 (50%)
	Rep2	6/7 (85.7%)	9/11 (81.8%)	4/12 (33.3%)	22/53 (41.5%)
	Rep3	12/13 (92.3%)	9/12 (75%)	10/14 (71.4%)	35/51 (68.6%)
**Counts**	Rep1	1/2 (NA)	2/2 (NA)	5/15 (33.3%)	8/14 (57.1%)
	Rep2	4/4 (NA)	3/3 (NA)	5/15 (33.3%)	7/12 (58.3%)
	Rep3	3/3 (NA)	3/3 (NA)	6/13 (46.2%)	7/12 (58.3%)
**All Others**	Rep1	23/24 (95.8%)	2/3 (NA)	22/24 (91.7%)	28/28 (100%)
	Rep2	34/34 (100%)	5/6 (83.3%)	23/23 (100%)	30/30 (100%)
	Rep3	22/22 (100%)	1/2 (NA)	23/24 (95.8%)	36/36 (100%)

Supplementary Table S8

Supplementary Table S8. Sub-questions with responses containing inaccurate statement(s) from at least one replicate.
Red: inaccurate statement; Blue: accurate statement; NA: no statement found to address the question. The response is incorrect to a sub-question from a replicate if it contain any incorrect statements. The response is consistently incorrect if available responses from all replicates are incorrect.

					Statement Indices			Consistently Incorrect	
Case	Category	Panel	Sub-questions addressed by statements	Ground Truth	Replicate 1	Replicate 2	Replicate 3	YES/ NO	YES %
Clonal	Color Perception	A	Determine the color of clusters for A	Cluster 1 is grey, cluster 2 is yellow, cluster 3 is purple, cluster 4 is red	<32><34><35><36><37><111>	<32><35><39><40><44><124>	<34><36><37><38><108><121>	YES	
Clonal	Color Perception	B	Determine the color of clusters for B	Cluster 1 is grey, cluster 2 is yellow, cluster 3 is purple, cluster 4 is red	<42><44><113>	<49><50><51><124>	<43><44><110><121>	YES	
Clonal	Color Perception	C&D	Determine the color of clusters for C&D	Cluster 1 is grey, cluster 2 is yellow, cluster 3 is purple, cluster 4 is red	<49><51>	<17><53><54><124>	<47><48><49><50><121>	NO	
Clonal	Color Perception	E&F	Determine the color of clusters for E&F	Cluster 1 is grey, cluster 2 is yellow, cluster 3 is purple, cluster 4 is red	<57><58><59><60><61><62><63>	<58><59><60><61><63><64><65><66><67><68><124>	<54><56><58><60><61><63><64><121>	YES	
Clonal	Color Perception	G	Determine the color of clusters for G	Cluster 1 is grey, cluster 2 is yellow, cluster 3 is purple, cluster 4 is red	<69><74><84>	<71><73><74><75><123><124>	<68><71><72><119><121>	YES	77.8%
RNA	Color Perception	A	Determine colors for the T/M groups	Blue for "T" and red for "T"	NA	<9> <113>	<7><8><127>	NO	
RNA	Color Perception	B	Determine color for DE genes	Red for "Up", Black for "Down", and blue for others	<13><14>	<17><117>	<17><18><19><130>	YES	
YY1	Color Perception	NA	Determine the color of the two groups of circles	The two groups of circles are blue and green respectively.	<139><141><142><143><144><145><146><149><151><153><155><158><159><160><213><214>	<136><137><138><139><140><141><144><145><152><165><210><212>	<143><144><145><146><147><148><157><158><159><160><206><208>	YES	
ZNF71	Color Perception	A	Determine color for patients group	KRAB $\leq$ 1.5 green, KRAB > 1.5 red.	<5><6><25><26><27>	<6><8><32>	<5><7><29>	YES	

Clonal	Counts	A	Sort the cluster by size in A	Cluster size from large to small should be : cluster 1, cluster2, cluster3, cluster 4	<34><35><36><37>	<38><39><40><43><44>	<35><36><37><38>	YES	
Clonal	Counts	E&F	Determine the size of clusters for E&F	There are 3 clusters in panel E, from large to small: cluster 2, cluster 4, cluster3. There are 2 clusters in panels F, from large to small: cluster 4, cluster 3	<56><57><58><59><60><61><62><63><65><66><128>	<58><59><60><61><63><64><65><66><67><68>	<53><54><56><58><60><61><63><64><126>	YES	**75.0%**
RNA	Counts	A	Determine the number of replicates	Replicate # = 2	<109>	NA	NA	NA	
YY1	Counts	NA	Determine the number of circles for blue and green groups	There are 3 blue and 3 green circles	<140><141><143><144><145><146>	<137><139><140><141><145>	<144><146><147><148><157>	YES	
Clonal	Positional Inference	B	Describe the change of cluster 3.	Cluster 3 shows a clear upward trajectory, starting from low.	<44>	<50>	<43>	NO	
Clonal	Positional Inference	B	Determine method used to determine an increase in VAF from primary to recurrent	Simply compare the value on y-axis between primary and recurrent	NA	<46><47>	<41>	YES	
Clonal	Positional Inference	C&D	Determine the major cluster at the time of sample taken	Cluster 2 is the major cluster in panel C, and cluster 4 is the major cluster in panel D	<49><50><51><52><53><54><127>	<53><54><55>	<47><48><49><50><125>	NO	**55.0%**
Clonal	Positional Inference	G	Identify the leaves on the tree structure	Only cluster 3 and 4 are at the leaves	<74><75><77><120 >	<73>74><75><76 >	<71><72><119>	YES	
RNA	Positional Inference	B	Determine the FC of JUNB	FC = ~3.5	<38>	<41>	<41>	YES	
YY1	Positional Inference	NA	Determine gene orders	Left to right: EVL DEGS2 YY1 SLC25A29 SLC25A47 WARS1 WDR25	<3>	<3>	<4>	NO	
YY1	Positional Inference	NA	Cite examples to explain arrow heads	YY1: "+" strand; DEGS2, EVL: "-" strand	<11><12>	<15><16>	<17>	NO	

YY1	Positional Inference	NA	Determine expressed genes from strand-specific RNA-Seq track	EVL, YY1, WARS1 are expressed	<21><22><23><25><27><29><31>	<27><29><31><33><38>	<29><30><31><32><38>	YES
YY1	Positional Inference	NA	Determine the genes expression base on the three histone modifications	See **Supplementary Table 5**	<47><48><49><50><51><52><53><54><56><57><58><60><62><63>	<55><56><57><59><61><64><65><66>	<54><55><57><59><61><62><64><65>	YES
YY1	Positional Inference	NA	Determine the positional relationship of genomic region "A"s with genes	All the "A" regions are overlapping with promoters of active genes	<77><78><79>	<76><77>	<80><81><82>	YES
YY1	Positional Inference	NA	Determine histone modification patterns for genomic regions "A"s	All "A" regions are H3K4me3+, H3K27ac+, and H3K27me3-.	<81><82><83>	<79><80><81>	<85><86><87>	NO
YY1	Positional Inference	NA	Determine potential function in transcription regulation of genes for genomic regions "A"s	All "A" regions are active promoters for expression activation.	<85><86><87><91>	NA	<91><93><95>	YES
YY1	Positional Inference	NA	Determine the positional relationship of genomic region "I"s with genes	All the "I" regions are overlapping with promoters of inactive genes	NA	<89>	NA	NO
YY1	Positional Inference	NA	Determine histone modification patterns for genomic regions "I"s	All "A" regions are weak in H3K4me3, H3K27ac-, and H3K27me3+.	<95><96>	<91><92><93>	<100><101><102>	YES
YY1	Positional Inference	NA	Determine potential function in transcription regulation of genes for genomic regions "I"s	All "I" regions are inactive promoters for expression repression.	<100>	<95><102>	NA	YES
YY1	Positional Inference	NA	Determine histone modification patterns for genomic regions "E"s	All "E" regions are H3K27+, week H3K4me3, and H3K27me3-	<107><109><110>	<107><108><110>	<111><113><114>	NO
YY1	Positional Inference	NA	Determine the interacting genomic regions indicated by blue and green circles.	The interaction, from left to right: Blue circles: A1::A2, I1::A2, A2::A3. Green circles: E1::A2, E2::A2, E3::A2.	<140><143><149><151><153><155><156><157><213><218><220>	<147><149><151><152><153><154><157><159><160><161><212><220><222>	<151><152><153><154><155><156><207>	YES

ZNF71	Positional Inference	A	Determine the survival prognosis by ZNF71 KRAB	Patients with a ZNF71 KRAB expression ≤ 1.5 have a better survival rate	<27>	<10><32><33>	<7><28><29>	NO	
ZNF71	Positional Inference	B	Determine the average expression of ZNF71 KRAB for different groups	The average expression for the resistant group is around 3. The average expression for the sensitive group is around 1.5.	<33><34><35>	<37><38><39>	<36><38>	YES	
ZNF71	Positional Inference	C	Determine the genes interacted with ZNF71 in LUAD tumors	In LUAD tumors, only JUNB and SAMHD1 are shown interacting with ZNF71. No more.	<16>	<22>	<17>	NO	
Clonal	Domain Knowledge	C&D	Explain the p-value in C&D	p-value testing for non-zero prevalence	<17>	<18><117>	<16>	YES	
RNA	Domain Knowledge	A	Explanation of PC1 and PC2	PC1 and PC2 mean % of variance explained by the first two components	<25><27><28><29><30><31><32>	<30><32><34><35><36><114><129>	<30><32><33><34><35><36><37>	NO	
YY1	Domain Knowledge	NA	Strand-specificity of RNA-Seq	Depending on protocol of library preparation, not all RNA-Seq data is strand-specific.	<18>	NA	NA	NO	**25.0%**
ZNF71	Domain Knowledge	D	Determining the relationship between changes in CD27 expression and trends in PQ-401 sensitivity	As the expression of CD27 mRNA increases, the ln(EC50) value of PQ-401 decreases, suggesting an increased sensitivity to PQ-401.	<45><46><47>	<45><46><47><48><49>	<45><46><47><48>	NO	
Clonal	Plot recognition	B	Determine labels of x and y axis	X-axis means tumor groups and y-axis means VAF values	NA	<11>	<9><41>	YES	**37.5%**
Clonal	Plot recognition	C&D	Determine/explain the plot type	Bell plot or simplified fish plot.	<13><114>	<14>	<11><17>	YES	

Clonal	Plot recognition	C&D	Explain the color areas	There are four colored areas to explain: orange, red, purple and gray.	NA	<17>	NA	NO	
Clonal	Plot recognition	C&D	Illustrate the way to determine the major cluster at a time point	The width covered by the area on the y-axis determine the size of a cluster	<48>	<52>	<46>	YES	
Clonal	Plot recognition	E&F	Determine/explain the plot type	There are circles not hexagons in the plot.	<116><117><128>	<119><120><121>	<115><116><117>	NO	
Clonal	Plot recognition	G	Determine/explain the plot type	Sizes are all equal and do not present prevalence.	<26><27>	<25><26>	<30>	NO	
YY1	Plot recognition	NA	Explain how the circles and dash lines indicate interacted genomic regions	Circle indicates interaction of genomic regions from the x-axis pointed by dash lines	<124><127>	<122><126>	<126><127><128><130>	NO	
ZNF71	Plot recognition	C	Determine the plot type	Gene Interaction Diagram	<14><15><37>	<21><41>	<15><16><42>	NO	
Clonal	Others	A&B	Provide suggestion to improve data presentation of A &B	Panel B dose not have densely packed points.	<96>	NA	NA	NO	
Clonal	Others	A&B	Provide suggestion to improve data presentation of A&B	Axes in Panels A and B already labeled clearly.	NA	NA	<84>	NO	
Clonal	Others	G	Provide suggestion to improve data presentation of G	The meaning of '*P' and '*R' has already clarified in the legend	<98>	NA	NA	NO	**20.0%**
RNA	Others	C	Comments to write the full name of pathway	Full name of pathy already show on the plot	<85>	NA	NA	NO	
ZNF71	Others	D	Determine gene shown on the Y-axis	CD27	<23>	<30>	<25>	YES	

SUPPLEMENTARY METHODS

Supplementary Methods S1

Supplementary Methods S1: Case background and authors' interpretations of the figures.

The "RNA" case study

Multiple myeloma (MM) is a hematologic malignancy predominantly proliferating within the bone marrow (BM). The BM stromal cells are known to support tumor growth and promote drug resistance [1]. RNA-Seq gene expression data from our early work [2] compares MM cells in trans-well coculture with BM stromal cells to those in monoculture. Results from PCA analysis in **Supplementary Figure S1** echoed the findings from our earlier work [2], showing substantial transcriptomic changes in MM cells induced by BM stromal cells (**Supplementary Figure S1A**). The upregulation in the expression of SOCS3 and JUNB acts as established positive controls drawn from the literature (**Supplementary Figure S1B**). KEGG pathway enrichment analysis pinpointed the JAK-STAT signaling pathway among the top hits for upregulated genes (**Supplementary Figure S1C**). This pathway is typically activated by IL-6 secreted from the stromal cells when cocultured with MM cells [3]. The observation supported that BM stromal cells induce substantial gene expression changes in MM cells, including the expression upregulation of key signaling pathways such as JAK-STAT and relevant regulatory genes such as SOCS3 and JUNB [2].

The "ZNF71" case study

Zinc finger protein 71 (ZNF71) is a pivotal member of a seven-gene assay we previously developed to predict chemotherapy efficacy for NSCLC [4]. As part of the expansive KRAB zinc finger transcription factors family, termed KRAB-ZNFs, ZNF71 possesses the distinctive KRAB domain and functions as a transcriptional repressor. **Supplementary Figure S2** summarizes key findings from our previous for this TF in NSCLC: 1) ZNF71 KRAB's expression correlates with a diminished overall survival rate in NSCLC patients [5] (**Supplementary Figure S2A**); 2) Docetaxel-resistant NSCLC cell lines exhibit elevated ZNF71 KRAB expression levels in comparison to their chemotherapy-responsive counterparts [5] (**Supplementary Figure S2B**); 3) ZNF71 is centered in a comprehensive association network with genes crucial for the intracellular innate immune response (IIIR) and suppresses their expression [6] (**Supplementary Figure S2C**); and 4) Our gene association networks identified PQ-401 as a drugs repositioning candidate for NSCLC treatment: Its Ln(EC50) in NSCLC cell lines negatively correlates with CD27 mRNA expression (**Supplementary Figure S2D**), suggesting potential immune-enhancing effects induced by the drug in NSCLC therapy [6].

The "Clonal" case study

A tumor is composed of clonal and subclonal cell populations. Understanding the evolutionary relationships among these cell populations is crucial for deciphering tumor development and progression. Different (sub)clones acquire unique somatic mutations that often exhibit varied variant allele frequencies (VAFs), which can be used to infer their cellular prevalence within a tumor. The "clonal evolution" case study illustrated somatic mutations in a pair of primary and recurrent MM tumor samples. Our observations from **Supplementary Figure S3** included: 1) The scatterplot contrasting the VAFs of somatic mutations between the primary and recurrent MM tumors shows four distinct clusters, whereas high-impact mutations predominantly residing in the founding clone, denoted as cluster 1 (**Supplementary Figure S3A**); 2) The connected line plot illustrates the mean VAF of clusters 3 and 4 increases in the recurrent tumors as compared to the primary tumor, while the mean VAF of cluster 2 decreases and cluster 1 remains largely unchanged (**Supplementary Figure S3B**); 3) The bell plots, also known as simplified fish plots, provide a schematic representation of the tumor's evolutionary dynamics over time – new subclones emerge and substitute the founding clone and old subclones (**Supplementary Figures S3C&S3D**); 4) Spheres of cells illustrate cellular composition of the tumors at the time of sample taken – the founding clone disappears from both the primary and recurrent tumors and the dominant subclone in the primary tumor represented by cluster 2 disappears from the recurrent tumor (**Supplementary Figures S3E&S3F**); and 5) A branch-based tree illustrating the relational and seeding patterns shows the founding clone evolves into two separate subclones (cluster 2 and 4) and cluster 2 further gives rise to cluster 3 (**Supplementary Figure S3G**). These observations suggest that high-impact functional mutations facilitated the growth of the founding clone. During the development of the primary tumor, emergent mutations gave rise to a subclone (cluster 2), which eventually surpassed the founding clone in cellular dominance. Subsequent mutations led to the evolution of two additional (sub)clones (clusters 3 and 4 albeit with low abundance), originating from the founding and dominant (sub)clones, respectively. Upon the tumor's recurrence, subclone 4 underwent expansion, becoming the predominant population, while subclone 2 experienced a reduction in prevalence.

The "YY1" case study

Epigenetic regulation of gene expression operates through mechanisms such as post-translational modifications of histone proteins, DNA methylation, and chromatin-chromatin interactions. Active transcriptional promoters are typically marked by H3K4me3, while transcriptionally silent promoters are characterized by H3K27me3 [7]. Active enhancer regions, on the other hand, are often marked by H3K27ac [8]. The 3D structure of chromatin also plays a crucial role in transcriptional regulation through promoter-promoter and promoter-enhancer interactions [9]. In this case study, we explored the epigenetic landscapes surrounding the YY1 locus in GM12878, a human B-Lymphoid cancer cell line prioritized by ENCODE for comprehensive data generation. Our observations of **Supplementary Figure S4** are: 1) The YY1 locus is transcriptionally active, evident from its promoter regions enriched with H3K4me3 and H3K27ac but devoid of H3K27me3 (as indicated in the "A2" region of **Supplementary Figure S4**); 2) Adjacent genes, namely DEGS2, SLC25A29, and SLC25A47, appear transcriptionally inactive, marked by promoters enriched with H3K27me3 (I1 and I2 regions). Conversely, EVL and WARS1 genes display transcriptional activity, resembling a histone modification pattern of YY1 (A1 and A3 regions); 3) Four presumptive enhancer regions characterized by H3K27ac are found: three between DEGS2 and YY1 (E1, E2, and E3 regions) and one immediately downstream of YY1 (E4 region); 4) PLAC-seq 3D chromatin interaction data signifies YY1 promoter interactions with the three upstream enhancers (indicated as green circles on the heatmap in **Supplementary Figure S4**). Additionally, the YY1 promoter appears to interact with the promoters of EVL (left circle), DEGS2 (middle circle), and WARS1 (right circle). The observations identified YY1 as a center element of a local chromatin-chromatin interaction hub, which included three upstream enhancers, three promoters of nearby genes with different transcriptional status, and the promoter of YY1 itself.

References

1 Chen, W. C., Hu, G. & Hazlehurst, L. A. Contribution of the bone marrow stromal cells in mediating drug resistance in hematopoietic tumors. *Curr Opin Pharmacol* **54**, 36-43 (2020). https://doi.org:10.1016/j.coph.2020.08.006

2 Dziadowicz, S. *et al.* Bone Marrow Stroma-induced Transcriptome and Regulome Signatures of Multiple Myeloma. *Cancers* **14**, 927 (2022).

3 Hideshima, T. & Anderson, K. C. Signaling Pathway Mediating Myeloma Cell Growth and Survival. *Cancers* **13**, 216 (2021). https://doi.org:10.3390/cancers13020216

4 Guo, N. L. *et al.* A Predictive 7-Gene Assay and Prognostic Protein Biomarkers for Non-small Cell Lung Cancer. *EBioMedicine* **32**, 102-110 (2018). https://doi.org:10.1016/j.ebiom.2018.05.025

5 Ye, Q. *et al.* Molecular Analysis of ZNF71 KRAB in Non-Small-Cell Lung Cancer. *Int J Mol Sci* **22** (2021). https://doi.org:10.3390/ijms22073752

6 Ye, Q. *et al.* Multi-Omics Immune Interaction Networks in Lung Cancer Tumorigenesis, Proliferation, and Survival. *Int J Mol Sci* **23** (2022). https://doi.org:10.3390/ijms232314978

7 Barski, A. *et al.* High-resolution profiling of histone methylations in the human genome. *Cell* **129**, 823-837 (2007). https://doi.org:10.1016/j.cell.2007.05.009

8 Creyghton, M. P. *et al.* Histone H3K27ac separates active from poised enhancers and predicts developmental state. *Proc Natl Acad Sci U S A* **107**, 21931-21936 (2010). https://doi.org:10.1073/pnas.1016071107

9 Hu, G. *et al.* Transformation of Accessible Chromatin and 3D Nucleome Underlies Lineage Commitment of Early T Cells. *Immunity* **48**, 227-242 e228 (2018). https://doi.org:10.1016/j.immuni.2018.01.013

Supplementary Methods S2

Supplementary Methods S2: Prompts used to guide GPT-4V to interpret bioinformatics figures.

The "RNA" case study

Input figure

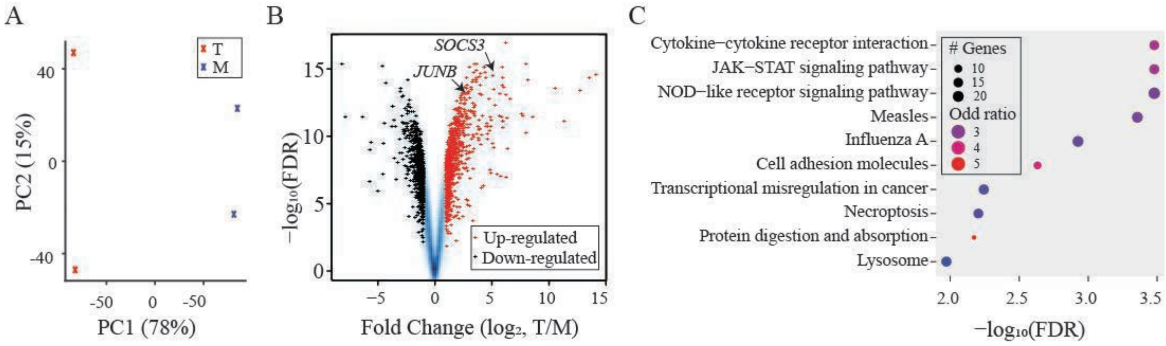

Prompts for the initial assessment:

Acting as a bioinformatics professional proficient in RNA-Seq data analysis, you are interpreting a figure to better appreciate transcription response of multiple myeloma cells (MMs) to bone marrow stromal cells (BMSCs). You also have extensive laboratory research experience in bone marrow tumor microenvironment of MM. In this task, expression profile of MM cell line RMPI8226 in trans-well coculture (T) with a BMSC cell line was compared to in monoculture (M). The attached figure illustrates the differential expression between the two conditions. Please do the following:

1. Provide an overview on each panel.
2. Are there any specific suggestions to improve the data presentation of the figure?
3. Draft a title and figure legend. Make sure to include details.
4. Draft a paragraph to describe the findings from the figure. This is one paragraph for result part of a manuscript. 150 words or less.

Prompts for the in-depth assessment:

Acting as a bioinformatics professional proficient in RNA-Seq data analysis, you are interpreting a figure to better appreciate transcription response of multiple myeloma cells (MMs) to bone marrow stromal cells (BMSCs). You also have extensive laboratory research experience in bone marrow tumor microenvironment of MM. In this task, expression profile of MM cell line RMPI8226 in trans-well coculture (T) with a BMSC cell line was compared to in monoculture (M). The attached figure illustrates the differential expression between the two conditions. Please do the following:

1. Provide an overview on each panel.
2. For panel A, what do the numbers in parentheses mean?
3. For panel B, the positions of SOCS3 and JUNB are indicated by arrow heads. Estimate the values from the x-axis for the two genes. Are the two genes up-regulated or down-regulated? Based on your findings in the literature, why are the two genes chosen and are these results expected?
4. For panel C, pick the top two pathways and explain the criteria. It is not indicated on the plot whether upregulated or downregulated genes were used for the pathway enrichment analysis. However, by correlating the findings to existing knowledge in the literature, which group of genes were likely used?
5. Are there any specific suggestions to improve the data presentation of the figure?
6. Draft a title and figure legend. Make sure to include details.
7. Draft a paragraph to describe the findings from the figure. This is one paragraph for result part of a manuscript. 150 words or less.

The “ZNF71” case study

Input figure

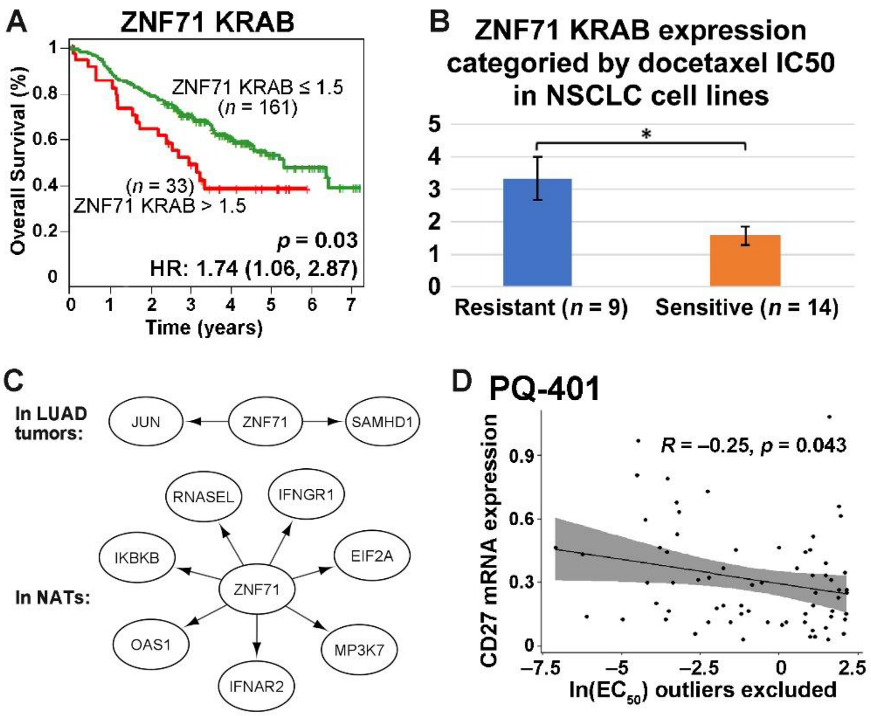

Prompts for an in-depth assessment:

Acting as a bioinformatics professional. Please do the following:

1. Provide an overview on each panel.
2. Panel A: Is ZNF71 KRAB favorable or unfavorable? Why?
3. Panel B: Is ZNF71 KRAB resistant or sensitive to docetaxel? Why and be quantitative?
4. Panel C: Which genes are associated with ZNF71 in LUAD tumors? Which genes are associated with ZNF71 in non-cancerous adjacent tissues?
5. Panel D: What is the R value in Panel C? What is the indication on the expression of CD27 and PQ-401 resistance?

The “Clonal” case study

Input figure

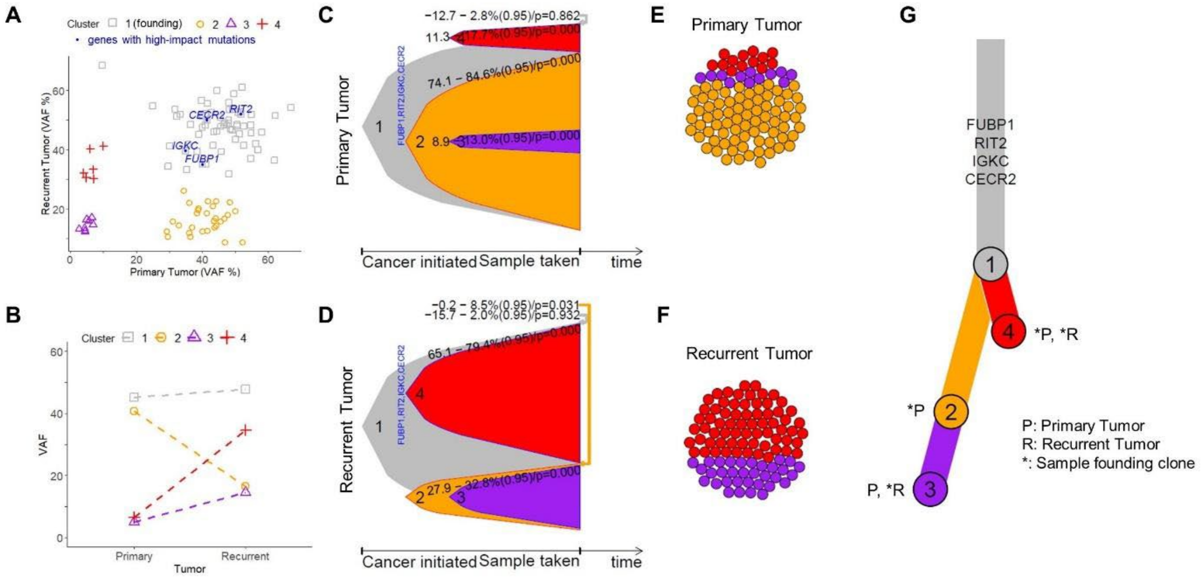

Prompts for an in-depth assessment:

Acting as a bioinformatics professional proficient in cancer genomics data analysis, you will help interpret a figure to better appreciate clonal evolution in multiple myeloma (MM). The attached figure illustrates the results of analyzing somatic mutations detected in a pair of primary and recurrent tumor samples from a patient diagnosed with MM. Please do the following:

1. Provide an overview on each panel. Include the type of plot in the description, e.g., scatter plot, bar plot, etc.
2. Panel A. Identify the number of clusters and their colors. Sort the clusters by size.
3. Panel B. Identify clusters showing an increase in VAF from primary to recurrent.
4. Panels C and D. Identify the major cluster at the time of sample taken for each panel.
5. Panels E and F. Sort the clusters by size for each panel.
6. Panel G: Identify cluster at the root and clusters are the leaves.
7. Are there any specific suggestions to improve the data presentation of the figure?
8. Draft a title and figure legend. Make sure to include details.
9. Draft a paragraph to describe the findings from the figure. This is one paragraph for result part of a manuscript. 150 words or less.

The “YY1” case study

Input figure

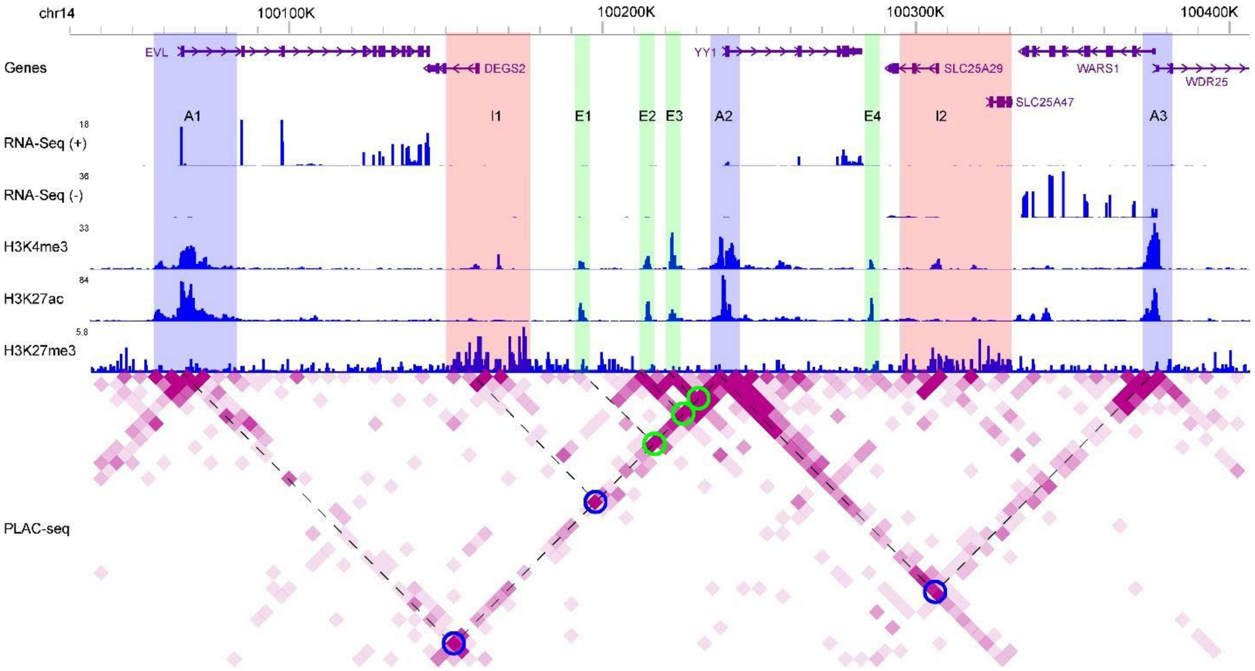

Prompts for the initial assessment:

Acting as a bioinformatics professional proficient in RNA-Seq, ChIP-seq, and 3D chromatin analysis, you will help interpret a figure to appreciate transcription regulation at chromatin level for YY1 in GM12878. The attached figure is a screen shot from WashU genome browser, encompassing gene annotation, expression data, ChIP-Seq data and chromatin-chromatin interaction data, for a genomic region enclosing YY1. The aim is to infer transcriptional regulatory mechanisms for YY1 from the epigenomic data. Please do the following.

1. Go over the tracks and explain specific findings addressing the aim.
2. Are there any specific suggestions to improve the data presentation of the figure?
3. Draft a title and figure legend. Make sure to include details.
4. Draft a paragraph to describe the findings from the figure. This is one paragraph for result part of a manuscript. 150 words or less.

Prompts for the in-depth assessment:

Acting as a bioinformatics professional proficient in RNA-Seq, ChIP-seq, and 3D chromatin analysis, you will help interpret a figure to appreciate transcription regulation at chromatin level for YY1 in GM12878. The attached figure is a screen shot from WashU genome browser, encompassing gene annotation, expression data, ChIP-Seq data and chromatin-chromatin interaction data, for a genomic region enclosing YY1. The aim is to infer transcriptional regulatory mechanisms for YY1 from the epigenomic data. You will be guided with questions to identify findings addressing the aim. Please do the following.

1. Go over the RefSeq gene track and spell out the genes shown on the plot. Explain the presentation of gene structure. For examples what does the arrowhead mean.
2. Check the expression data. What do “(+)” and “(-)” mean? Which genes are expressed and why?
3. Check the ChIP-seq data. Explain the association of H3K4me3, H3K27ac, and H3K27me3 with gene expression. Based on the three histone modifications, which genes are likely expressed, and which are not likely expressed?
4. Three groups of genomic regions are highlighted: “A”s in light blue, “I”s in pink, and “E”s in green. Identify the number of regions for each group.

5. For genomic regions in “A”s, what are their shared features in terms of positional relationship with genes and histone modification patterns? What is their potential function in transcription regulation?
6. How about genomic regions in “I”s? What is their potential function in transcription regulation?
7. How about genomic regions in “E”s? What is their potential function in transcription regulation?
8. On the heatmap for chromatin-chromatin interactions, there are several circles each connected with dashed lines. Explain what the circle means and how about the dashed lines connected to the circle. What are the common features of the circles in terms of indicated genomic region(s)?
9. There are two group of circles by colors. Identify the number of circles for each group.
10. Focus the circles in blue, identify their indicated interacting genomic regions.
11. Focus the circles in green, identify their indicated interacting genomic regions.
12. Are there any specific suggestions to improve the data presentation of the figure?
13. Draft a title and a legend for this figure based on our previous discussion. Make sure to include details.
14. Draft a paragraph to describe the findings from our previous discussion on transcription regulation of YY1. This is one paragraph for result part of a manuscript. 150 words or less.

SUPPLEMENTARY NOTES

Supplementary Notes S1

Supplementary Notes S1: Parsed, indexed, and annotated statements for the “RNA” case study.

Instruction

Indexing principle: The indexing format adopted is <Case-Replicate-Statement>. In this format, “Case” represents the case study number, with 1 for “RNA”, 2 for “ZNF71”, 3 for “Clonal”, and 4 for “YY1”. The term “Replicate” indicates the replicate number, which can be either 1, 2, or 3. “Statement” corresponds to the statement’s sequential position within a specific replicate of a case study. For example, the third statement (3) in the second replicate (2) of the “RNA” case study (1) is indexed as <1-2-3>. Furthermore, a letter “B” is appended to the “Case” slot if the chat history originates from prompts associated with the basal assessment model.

Color-coding: True statements in **blue**, false ones in **red** (with errors highlighted in **yellow**), and chitchats (non-informative statements) in **black**, which are excluded from evaluation. An {explanation} highlighted in yellow in a curly bracket would append to the end of the false statement.

Overall performance

For the calculation of overall performance, a statement is marked as true if the contents for all its indicated categories are correct.

Replicate	Number of accurate statements	Number of evaluated statements	Accuracy
RNA_basal_rep1	57	63	90.48%
RNA_basal_rep2	64	66	96.97%
RNA_basal_rep3	66	69	95.65%
RNA_ indepth_rep1	84	88	95.45%
RNA_ indepth_rep2	89	95	93.68%
RNA_ indepth_rep3	102	106	96.23%

The “RNA” case study (basal assessment 1)

Q

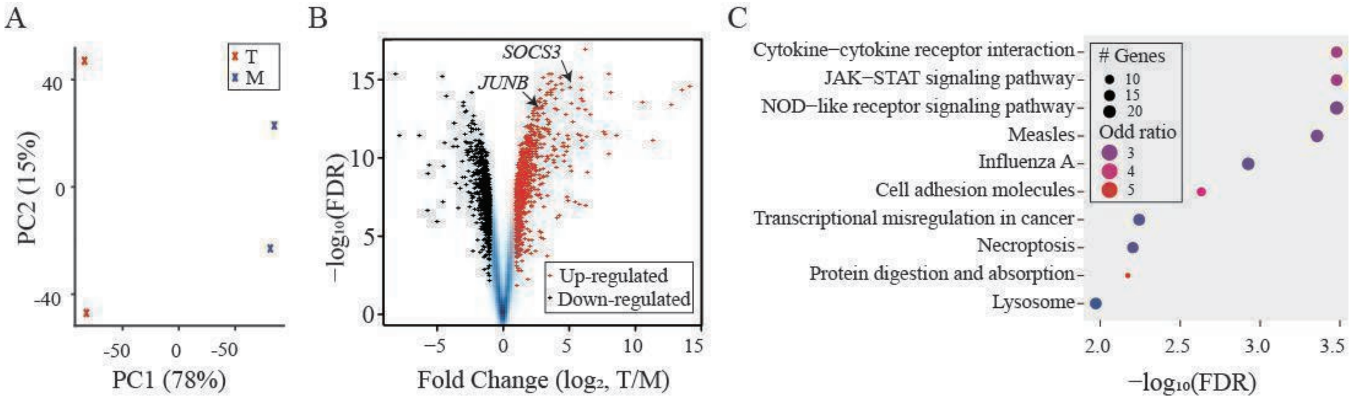

Acting as a bioinformatics professional proficient in RNA-Seq data analysis, you are interpreting a figure to better appreciate the transcription response of multiple myeloma cells (MMs) to bone marrow stromal cells (BMSCs). You also have extensive laboratory research experience in the bone marrow tumor microenvironment of MM. In this task, the expression profile of MM cell line RMPI8226 in trans-well coculture (T) with a BMSC cell line was compared to monoculture (M). The attached figure illustrates the differential expression between the two conditions. Please do the following: 1. Provide an overview of each panel.

<1B-1-1> Certainly!
<1B-1-2> The figure you provided consists of three panels (A, B, and C), each showcasing different aspects of the differential gene expression analysis between the RMPI8226 MM cell line in trans-well coculture (T) with a BMSC cell line compared to monoculture (M). Here's a breakdown of each panel:.
<1B-1-3> 1. Panel A Principal Component Analysis (PCA) Plot: Overview:
<1B-1-4> PCA is a dimensionality reduction technique that captures the variance in the dataset using fewer principal components.
<1B-1-5> It's often used in genomics to visualize the overall variation in the expression data.
<1B-1-6> Interpretation: The X-axis represents the first principal component (PC1), accounting for 78% of the variance, while the Y-axis shows the second principal component (PC2), capturing 15% of the variance.
<1B-1-7> The plot depicts two distinct clusters, one for the T (trans-well coculture) and one for the M (monoculture) condition.
<1B-1-8> The separation indicates that there are significant differences in gene expression between these two conditions.
<1B-1-9> 2. Panel B Volcano Plot:
<1B-1-10> Overview:
<1B-1-11> Volcano plots are used to visualize differential expression between two conditions.
<1B-1-12> The X-axis typically represents the fold change (log2 scale) between the two conditions, while the Y-axis shows the statistical significance of this change (-log10 of the False Discovery Rate or FDR).
<1B-1-13> Interpretation: The red points represent genes that are up-regulated in the T condition compared to M, while the blue points indicate genes that are down-regulated. {Black not blue are for down-regulated genes.}
<1B-1-14> The further a point is from the center along the X-axis, the higher its fold change.
<1B-1-15> The higher a point is on the Y-axis, the more statistically significant its differential expression.
<1B-1-16> Specific genes of interest, such as SOCS3 and JUNB, are labeled, implying their importance or relevance to the study.
<1B-1-17> 3.
<1B-1-18> Panel C Pathway Enrichment Analysis:

<1B-1-19> Overview:
<1B-1-20> Pathway enrichment analysis identifies biological pathways that are overrepresented in a given set of differentially expressed genes.
<1B-1-21> Interpretation: The Y-axis lists different biological pathways, while the X-axis provides the negative log10 of the FDR, indicating the significance of enrichment for each pathway.
<1B-1-22> The size of each dot represents the number of genes in the dataset associated with the respective pathway, and the color indicates the odd ratio, which is the ratio of observed to expected genes in the pathway.
<1B-1-23> Pathways to the right, like "Cytokine-cytokine receptor interaction" and "JAK-STAT signaling pathway," are significantly enriched among differentially expressed genes, suggesting that these pathways might play a crucial role in the response of MM cells to BMSCs.
<1B-1-24> In summary, this figure provides a comprehensive overview of the transcriptional changes occurring in the MM cell line RMPI8226 when cocultured with BMSCs.
<1B-1-25> The data highlights significant differences in gene expression between coculture and monoculture conditions and points to specific biological pathways that might be critical for the interaction between MM cells and BMSCs.

Q

2. Are there any specific suggestions to improve the data presentation of the figure?

<1B-1-26> Certainly!
<1B-1-27> Here are some suggestions to improve the data presentation of the figure:.
<1B-1-28> 1. Panel A PCA Plot: Color Contrast:
<1B-1-29> While the blue and red colors are distinguishable, it could be helpful to choose colors that are colorblind-friendly to ensure accessibility for all readers.
<1B-1-30> Labels:
<1B-1-31> Consider adding a legend to clarify which color corresponds to which condition (T or M). {Legend (color and condition) already shown on the plot.}
<1B-1-32> Point Size:
<1B-1-33> If multiple replicates were used for each condition, consider increasing the point size or using different shapes for better visibility.
<1B-1-34> 2. Panel B Volcano Plot: Threshold Lines:
<1B-1-35> Add horizontal lines to indicate commonly used significance thresholds (e.g., FDR <1B-1- 0.05).
<1B-1-36> This will help readers quickly identify which genes surpass the significance threshold.
<1B-1-37> More Gene Labels:
<1B-1-38> Labeling only two genes might not be enough to convey the most significant findings.
<1B-1-39> Consider labeling a few more of the most up or down-regulated genes, especially those furthest from the center.
<1B-1-40> Zoomed-In Sections:
<1B-1-41> If there are regions with a dense clustering of points, consider adding an inset or a "zoomed-in" section to provide more clarity.
<1B-1-42> 3. Panel C Pathway Enrichment Analysis:
<1B-1-43> Color Legend:
<1B-1-44> Add a color scale legend for the odd ratios, helping readers understand the meaning behind the different colors. {Color scale legend for odd ratio already shown.}
<1B-1-45> Size Legend:
<1B-1-46> Similarly, a legend indicating the scale for the number of genes represented by the dot sizes would be beneficial. {Legend for number of genes already shown.}

<1B-1-47> More Descriptive Y-Axis Labels:
<1B-1-48> Some pathway names, like "Transcriptional misregulation in cancer," are quite long and can be truncated or abbreviated for clarity, with full names provided in a footnote or supplementary material.
<1B-1-49> Highlighting Key Pathways:
<1B-1-50> If some pathways are of particular importance or relevance to the study, consider highlighting them in a different color or with an outline to draw the reader's attention.
<1B-1-51> General Suggestions: Figure Title:
<1B-1-52> A descriptive title at the top of the entire figure can help set the context for readers.
<1B-1-53> Uniformity:
<1B-1-54> Ensure that font size, type, and style are uniform across all panels for consistency.
<1B-1-55> Figure Size and Resolution:
<1B-1-56> Ensure that the figure is high-resolution, especially if it's intended for print publication.
<1B-1-57> This ensures that all labels, points, and lines are clear and readable.
<1B-1-58> Supplementary Material: Consider providing supplementary tables or lists that offer detailed information on the top differentially expressed genes or pathways, especially if space constraints limit the number of genes or pathways that can be displayed in the figure.
<1B-1-59> Improving the presentation in these ways can make the figure more informative, accessible, and reader-friendly.

Q

3. Draft a title and figure legend. Make sure to include details.

<1B-1-60> Title: "Differential Gene Expression and Pathway Enrichment Analysis of RMPI8226 MM Cells in Trans-well Coculture with BMSCs Compared to Monoculture".
<1B-1-61> Figure Legend:.
<1B-1-62> Figure X.
<1B-1-63> Comprehensive analysis of transcriptional changes in RMPI8226 MM cells when cocultured with BMSCs.
<1B-1-64> (A) Principal Component Analysis (PCA) of gene expression profiles.
<1B-1-65> PC1 and PC2 account for 78% and 15% of the data variance, respectively.
<1B-1-66> Red crosses represent samples from the trans-well coculture condition (T) and blue crosses represent monoculture condition (M).
<1B-1-67> (B) Volcano plot showcasing the differential gene expression between the two conditions.
<1B-1-68> The X-axis represents log2 fold change (T vs. M), and the Y-axis displays -log10(FDR).
<1B-1-69> Red dots indicate up-regulated genes, while blue dots indicate down-regulated genes in the coculture condition. {Black means down-regulated. Not blue.}
<1B-1-70> Selected genes of particular interest, such as SOCS3 and JUNB, are labeled.
<1B-1-71> (C) Pathway enrichment analysis of differentially expressed genes.
<1B-1-72> Pathways are displayed on the Y-axis, and the significance of enrichment is shown as -log10(FDR) on the X-axis.
<1B-1-73> The size of each dot represents the number of genes associated with that pathway, while the color indicates the odd ratio (with the legend provided).
<1B-1-74> Note: The datasets were generated from three independent biological replicates for each condition. {Duplicates not triplicates.}
<1B-1-75> This title and legend provide a concise yet detailed description of the figure, ensuring readers can interpret the data without referring to the main text frequently.

Q

4. Draft a paragraph to describe the findings from the figure. This is one paragraph for the result part of a manuscript. 150 words or less.

<1B-1-76> In our comprehensive transcriptional assessment of RMPI8226 MM cells, discernible variations were observed when cocultured with BMSCs compared to monocultured conditions.
<1B-1-77> The PCA plot delineated a clear separation between the two conditions, with the first two principal components capturing a substantial 93% of the total variance (Panel A).
<1B-1-78> Notably, the volcano plot revealed a multitude of genes displaying marked differential expression.
<1B-1-79> Genes such as SOCS3 and JUNB emerged as notably upregulated in the coculture condition (Panel B).
<1B-1-80> Further pathway enrichment analysis pinpointed several pathways being significantly enriched.
<1B-1-81> These encompassed the "Cytokine-cytokine receptor interaction" and the "JAK-STAT signaling pathway," suggesting a potential pivotal role in mediating the interactions between MM cells and BMSCs in the bone marrow tumor microenvironment (Panel C).
<1B-1-82> Collectively, our findings underscore the profound influence of BMSCs on the transcriptional landscape of RMPI8226 MM cells.

The “RNA” case study (basal assessment 2)

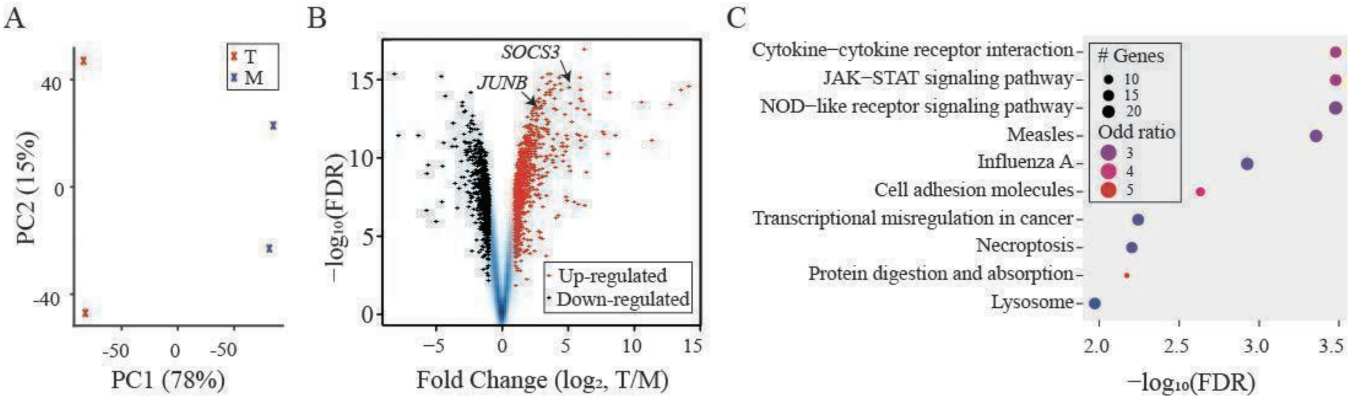

Acting as a bioinformatics professional proficient in RNA-Seq data analysis, you are interpreting a figure to better appreciate the transcription response of multiple myeloma cells (MMs) to bone marrow stromal cells (BMSCs). You also have extensive laboratory research experience in the bone marrow tumor microenvironment of MM. In this task, the expression profile of MM cell line RMPI8226 in trans-well coculture (T) with a BMSC cell line was compared to monoculture (M). The attached figure illustrates the differential expression between the two conditions. Please do the following: 1. Provide an overview of each panel.

<1B-2-1> Of course, based on the provided figure and your description, here's an interpretation of each panel:.
<1B-2-2> Panel A: Principal Component Analysis (PCA) Plot
<1B-2-3> This plot provides a visualization of the overall variance in the gene expression data between the two conditions: monoculture (M) and trans-well coculture (T) of MM cells with BMSCs.
<1B-2-4> The x-axis (PC1) explains 78% of the variance, while the y-axis (PC2) explains 15%.
<1B-2-5> Each point on the plot represents a sample, and the spatial distance between points indicates the degree of similarity in gene expression patterns.
<1B-2-6> There's a clear separation between the two conditions, with the T (in red) and M (in blue) clusters being distinct.
<1B-2-7> This indicates significant differences in gene expression between the two conditions.
<1B-2-8> Panel B: Volcano Plot
<1B-2-9> This plot visualizes the differential expression of genes between the T and M conditions.
<1B-2-10> The x-axis shows the fold change in gene expression (log2 scale), with genes to the right being up-regulated in the T condition and those to the left being down-regulated.
<1B-2-11> The y-axis displays the statistical significance of these changes in terms of -log10(FDR), with higher values indicating higher significance.
<1B-2-12> Genes that are highly up-regulated and statistically significant are in the upper right corner (in red), and those that are highly down-regulated and statistically significant are in the upper left corner (in blue). {Down-regulated genes are in black.}
<1B-2-13> Two genes, "SOCS3" and "JUNB", are specifically labeled, suggesting they might be of particular interest or have shown significant differential expression between the two conditions.
<1B-2-14> Panel C: Pathway Enrichment Analysis
<1B-2-15> This panel provides insights into the biological pathways that are enriched among the differentially expressed genes.
<1B-2-16> The x-axis indicates the significance of the enrichment in terms of -log10(FDR).

<1B-2-17> Each dot represents a specific biological pathway, and the size of the dot represents the number of genes associated with that pathway.
<1B-2-18> The color of each dot represents the odd ratio, indicating the strength of the association between the differentially expressed genes and the given pathway.
<1B-2-19> Pathways such as "Cytokine-cytokine receptor interaction," "JAK-STAT signaling pathway," and "NOD-like receptor signaling pathway" appear to be highly significant and may play critical roles in the response of MM cells to BMSCs.
<1B-2-20> Overall Interpretation: The provided figure paints a comprehensive picture of the transcriptional changes that occur in MM cells when cocultured with BMSCs.
<1B-2-21> The clear separation in the PCA plot emphasizes the substantial difference in gene expression profiles between the two conditions.
<1B-2-22> The volcano plot pinpoints specific genes that are highly up-regulated or down-regulated.
<1B-2-23> Lastly, the pathway enrichment analysis provides a biological context, suggesting that certain signaling pathways and processes might be pivotal in mediating the interactions between MM cells and BMSCs in the bone marrow tumor microenvironment.

Q

2. Are there any specific suggestions to improve the data presentation of the figure?

<1B-2-24> Certainly!
<1B-2-25> While the figure is informative, there are always potential improvements that can enhance clarity and ease of interpretation.
<1B-2-26> Here are some suggestions to improve the data presentation:.
<1B-2-27> 1. Panel A (PCA Plot): Legend and Labels:
<1B-2-28> Ensure the legend is clear and easy to distinguish between the T and M conditions.
<1B-2-29> If not already done, consider using distinct colors or shapes for each condition.
<1B-2-30> Percent Variance:
<1B-2-31> For clarity, explicitly label the percent variance explained by each principal component directly on the axes (e.g., "PC1 (78% variance)").
<1B-2-32> Zooming:
<1B-2-33> If there are more samples, consider zooming into regions where data points overlap to provide better clarity.
<1B-2-34> 2. Panel B (Volcano Plot): Threshold Lines:
<1B-2-35> Add horizontal lines to indicate commonly used FDR thresholds (e.g., FDR = 0.05) and vertical lines for fold change thresholds (e.g., 2-fold).
<1B-2-36> This will help readers quickly identify genes of interest.
<1B-2-37> Highlight Key Genes:
<1B-2-38> While "SOCS3" and "JUNB" are labeled, consider highlighting other genes that might be of biological significance or interest.
<1B-2-39> Color Gradient:
<1B-2-40> Instead of just red and blue, use a gradient to represent the degree of up-regulation or down-regulation, which can provide more nuance to the visualization.
<1B-2-41> 3. Panel C (Pathway Enrichment Analysis): Y-axis Labeling:
<1B-2-42> Explicitly label the y-axis with the pathway names for better readability. {This already shown on the plot.}
<1B-2-43> If space is limited, rotating the text or using abbreviations can help.
<1B-2-44> Interactive Features:

<1B-2-45> If presenting digitally, consider using an interactive plot where hovering over a dot provides more details about the pathway (e.g., gene names, specific p-values).
<1B-2-46> Order of Pathways:
<1B-2-47> Rearrange the pathways in decreasing order of significance, with the most significant pathways at the top.
<1B-2-48> 4.
<1B-2-49> General: Consistent Color Scheme:
<1B-2-50> Ensure that the color schemes used in different panels are consistent.
<1B-2-51> For instance, if red represents the T condition in Panel A, it should represent the same in Panels B and C.
<1B-2-52> Title and Descriptive Captions:
<1B-2-53> Provide a clear and concise title for the figure, and consider adding descriptive captions below each panel to guide the reader.
<1B-2-54> Font Size and Style:
<1B-2-55> Ensure that all text is legible, especially when the figure is resized.
<1B-2-56> Use consistent font styles and sizes across panels.
<1B-2-57> High-Resolution Export:
<1B-2-58> If the figure is intended for publication, ensure it is exported in high resolution with lossless compression.
<1B-2-59> 5.
<1B-2-60> Additional Data:
<1B-2-61> If there's supplementary data or detailed results for some genes/pathways, consider providing an appendix or a link to a supplementary file.
<1B-2-62> This ensures that readers interested in delving deeper have access to detailed information.
<1B-2-63> Improving the presentation not only makes the data more aesthetically pleasing but also aids in conveying the findings more effectively to the audience.

Q

3. Draft a title and figure legend. Make sure to include details.

<1B-2-64> Title: "Differential Gene Expression and Pathway Enrichment Analysis in MM Cells Co-cultured with BMSCs".
<1B-2-65> Figure Legend:.
<1B-2-66> Figure 1: Transcriptional changes observed in MM cell line RMPI8226 when co-cultured with a BMSC cell line compared to monoculture conditions.
<1B-2-67> (A) Principal Component Analysis (PCA) plot showcasing variance in gene expression between MM cells in monoculture (M, shown in blue) and trans-well co-culture with BMSCs (T, shown in red).
<1B-2-68> PC1 accounts for 78% of the variance, while PC2 accounts for 15%.
<1B-2-69> (B) Volcano plot illustrating the differential expression of genes between the two conditions.
<1B-2-70> The x-axis depicts fold change in gene expression (log2 scale), with genes on the right being up-regulated and those on the left being down-regulated in the T condition relative to M.
<1B-2-71> The y-axis presents the statistical significance of these changes as -log10(FDR).
<1B-2-72> Genes "SOCS3" and "JUNB" are labeled, indicating their noteworthy differential expression.
<1B-2-73> (C) Pathway Enrichment Analysis indicating significant biological pathways associated with the differentially expressed genes.
<1B-2-74> Each dot represents a pathway, with the x-axis denoting the significance of enrichment as -log10(FDR).
<1B-2-75> Dot size corresponds to the number of genes in each pathway, while the color gradient signifies the odd ratio, reflecting the strength of the association.

<1B-2-76> Note: The data provides insights into the molecular mechanisms underlying MM cell interactions with the bone marrow stromal environment, highlighting the importance of specific genes and signaling pathways in this context.

Q

4. Draft a paragraph to describe the findings from the figure. This is one paragraph for the result part of a manuscript. 150 words or less.

<1B-2-77> In our examination of the transcriptional response of the MM cell line RMPI8226 to BMSC coculture, distinct transcriptional shifts were evident.
<1B-2-78> The Principal Component Analysis (PCA) in Figure 1A revealed a pronounced separation between monocultured MM cells and those co-cultured with BMSCs, with PC1 accounting for a striking 78% of the variance.
<1B-2-79> Delving into individual gene expression differences, the volcano plot (Figure 1B) spotlighted several genes with significant differential expression, notably the up-regulation of "SOCS3" and "JUNB".
<1B-2-80> Furthermore, pathway enrichment analysis shed light on potential biological implications of these transcriptional changes.
<1B-2-81> As shown in Figure 1C, pathways related to "Cytokine-cytokine receptor interaction", "JAK-STAT signaling", and "NOD-like receptor signaling" were among the most significantly enriched, underscoring their potential roles in mediating MM cell interactions within the bone marrow stromal environment.
<1B-2-82> These findings provide a foundational understanding of the molecular dialogue between MM cells and their surrounding niche.

The “RNA” case study (basal assessment 3)

Q

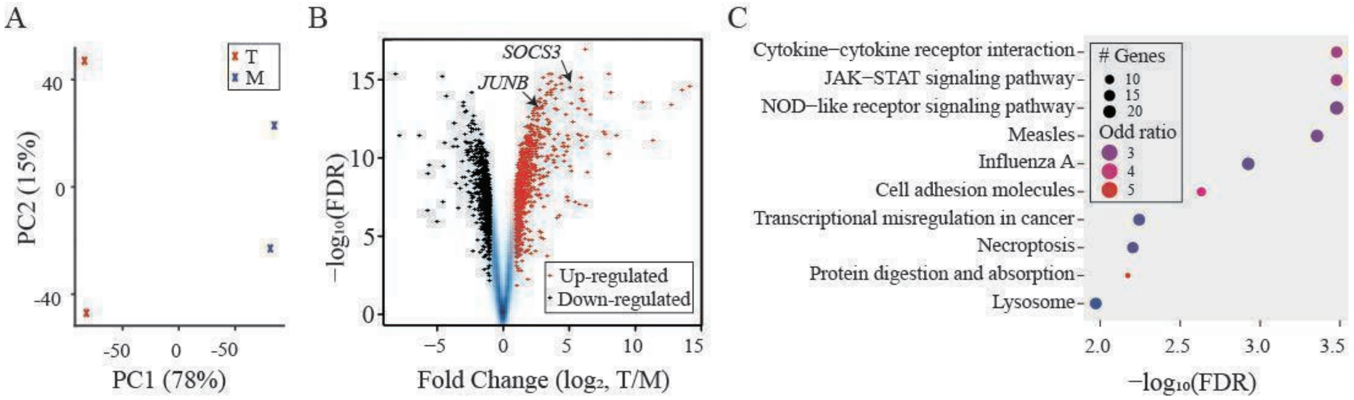

Acting as a bioinformatics professional proficient in RNA-Seq data analysis, you are interpreting a figure to better appreciate the transcription response of multiple myeloma cells (MMs) to bone marrow stromal cells (BMSCs). You also have extensive laboratory research experience in the bone marrow tumor microenvironment of MM. In this task, the expression profile of MM cell line RMPI8226 in trans-well coculture (T) with a BMSC cell line was compared to monoculture (M). The attached figure illustrates the differential expression between the two conditions. Please do the following: 1. Provide an overview of each panel.

<1B-3-1> Sure, based on the provided figure, here's an overview of each panel:.

<1B-3-2> Panel A: Principal Component Analysis (PCA) Plot

<1B-3-3> This plot represents a PCA analysis of the expression profiles from the MM cell line RPMI8226 when co-cultured with a BMSC cell line (T) versus when it's monocultured (M).

<1B-3-4> PCA is a dimensionality reduction method used to visualize the variance in large datasets.

<1B-3-5> Here, it helps us observe the overall transcriptional differences between the two conditions.

<1B-3-6> The X-axis represents the first principal component (PC1) which captures 78% of the total variance, while the Y-axis represents the second principal component (PC2) capturing 15% of the variance.

<1B-3-7> The blue and red crosses represent the transcriptional profiles of the monoculture (M) and trans-well co-culture (T) respectively.

<1B-3-8> The clear separation between them suggests significant transcriptional differences between the two conditions.

<1B-3-9> Panel B: Volcano Plot

<1B-3-10> This plot showcases the differential expression of genes between the MM cell line RPMI8226 in trans-well coculture (T) with a BMSC cell line compared to its monoculture (M).

<1B-3-11> The X-axis represents the fold change (in log2 scale) of gene expression between the two conditions.

<1B-3-12> Positive values indicate up-regulation in the co-culture, while negative values indicate down-regulation.

<1B-3-13> The Y-axis represents the statistical significance of this change in a -log10 scale.

<1B-3-14> Higher values mean more significant differences.

<1B-3-15> Genes with higher statistical significance and higher fold change are located in the top right (up-regulated) and top left (down-regulated) corners.

<1B-2-38> Two genes, SOCS3 and JUNB, are explicitly labeled, suggesting they might be of particular interest.

<1B-3-17> The red dots represent up-regulated genes, and the blue dots represent down-regulated genes. {Black dots other than blue dots represents down-regulated genes.}

<1B-3-18> .

<1B-3-19> Panel C: Pathway Enrichment Analysis

<1B-3-20> This plot displays the results of a pathway enrichment analysis, which determines which biological pathways are significantly represented among the differentially expressed genes.
<1B-3-21> The Y-axis lists different pathways, while the X-axis shows the significance (-log10 of FDR, or False Discovery Rate) of each pathway's enrichment.
<1B-3-22> The size of each dot represents the number of genes in the pathway, and the color represents the odd ratio, which indicates the likelihood that the genes from the dataset are part of a given pathway compared to random chance.
<1B-3-23> A higher odd ratio suggests a stronger association.
<1B-3-24> Several pathways are highlighted, including cytokine-cytokine receptor interaction, JAK-STAT signaling, and others.
<1B-3-25> These pathways might play a crucial role in the interaction between MM cells and BMSCs in the bone marrow tumor microenvironment.
<1B-3-26> In summary, this figure provides a comprehensive overview of the transcriptional changes in MM cells when co-cultured with BMSCs, highlighting specific genes of interest and potential biological pathways influenced by this interaction.

Q

2. Are there any specific suggestions to improve the data presentation of the figure?

<1B-3-27> Certainly!
<1B-3-28> Here are some suggestions to improve the data presentation of the figure:.
<1B-3-29> Panel A: Principal Component Analysis (PCA) Plot 1.
<1B-3-30> Label clarity: Add clear labels or legends for the data points representing monoculture (M) and trans-well coculture (T) to help viewers quickly understand the plot.
<1B-3-31> 2. Proportion of variance: Consider displaying the percentage of variance explained by other principal components (e.g., PC3, PC4) in a small inset or table, especially if they contribute significantly to the dataset's variance.
<1B-3-32> 3. Gridlines: Adding subtle gridlines can help in estimating the coordinates of the points more precisely.
<1B-3-33> Panel B: Volcano Plot 1.
<1B-3-34> Significance threshold: Add horizontal lines indicating common significance thresholds (e.g., p-value of 0.05) to quickly identify genes that surpass this threshold.
<1B-3-35> 2. Fold-change threshold:
<1B-3-36> Add vertical lines to mark common fold change thresholds (e.g., 2-fold or log2(2)) to easily identify highly upregulated or downregulated genes.
<1B-3-37> 3. Highlight key genes:
<1B-3-38> Besides SOCS3 and JUNB, consider highlighting or labeling other genes that might be of biological significance or those with extreme values of fold change and significance.
<1B-3-39> 4. Color choice:
<1B-3-40> Use a gradient color scale or another scheme to indicate the magnitude of fold change and significance, which can provide additional visual cues.
<1B-3-41> Panel C: Pathway Enrichment Analysis 1.
<1B-3-42> Interactive capabilities:
<1B-3-43> If presented online, consider adding interactive capabilities to the plot.
<1B-3-44> Hovering over a point could display the specific genes involved in that pathway or show additional information about the pathway.
<1B-3-45> 2. Scale clarity:
<1B-3-46> For the color scale representing the odd ratio, consider adding a legend or scale bar for clarity. {There is already scale to differentiate the color.}

<1B-3-47> 3. Sort pathways:
<1B-3-48> Sort pathways based on their significance or another meaningful metric to help readers quickly identify the most relevant pathways. {Sorted by FDR already}
<1B-3-49> 4. Pathway details:
<1B-3-50> For pathways of particular interest, consider providing a small inset or additional figure detailing the specific genes from the dataset involved in that pathway.
<1B-3-51> General suggestions: 1. Consistent color schemes: Ensure that colors used are consistent across all panels if they represent the same data points or conditions.
<1B-3-52> 2. Resolution and clarity: Ensure that the figure is of high resolution, especially if it's for print.
<1B-3-53> All text, labels, and data points should be clear and legible.
<1B-3-54> 3. Figure caption:
<1B-3-55> Provide a comprehensive figure caption that briefly describes each panel and any experimental or analytical details that might be essential for interpretation.
<1B-3-56> 4. Size and scale:
<1B-3-57> Depending on where this figure will be presented (e.g., journal article, presentation, poster), adjust the size and scale to ensure it's easily interpretable without overwhelming the viewer.
<1B-3-58> These enhancements can make the figure more informative and user-friendly, aiding in clearer data interpretation.

Q

3. Draft a title and figure legend. Make sure to include details.

<1B-3-59> Title: "Differential Gene Expression and Pathway Enrichment Analysis of RPMI8226 Multiple Myeloma Cells Co-cultured with BMSCs".
<1B-3-60> Figure Legend:.
<1B-3-61> Figure X: Transcriptional profiling and pathway analysis of RPMI8226 multiple myeloma cells in response to co-culture with bone marrow stromal cells.
<1B-3-62> (A) Principal Component Analysis (PCA)
<1B-3-63> Plot: Transcriptional variance is depicted between the RPMI8226 MM cells in monoculture (M, blue crosses) and in trans-well coculture with BMSCs (T, red crosses).
<1B-3-64> The X-axis displays the first principal component (PC1), accounting for 78% of the total variance, while the Y-axis displays the second principal component (PC2), capturing 15% of the variance.
<1B-3-65> (B) Volcano Plot:
<1B-3-66> Differential gene expression between the two conditions is illustrated.
<1B-3-67> The X-axis indicates the fold change in gene expression (log2 scale), with positive values showing up-regulation in the co-culture and negative values indicating down-regulation.
<1B-3-68> The Y-axis represents the statistical significance of these changes in a -log10 scale.
<1B-3-69> Genes with higher statistical significance and fold change are seen in the top corners.
<1B-3-70> Specific genes of potential significance, such as SOCS3 and JUNB, are labeled.
<1B-3-71> (C) Pathway Enrichment Analysis:
<1B-3-72> Significant biological pathways influenced by the differential gene expression are shown.
<1B-3-73> Pathways are listed on the Y-axis, with their enrichment significance represented on the X-axis (-log10 of FDR).
<1B-3-74> The size of each dot corresponds to the number of genes involved in that pathway, and the color indicates the odds ratio, with a darker hue suggesting a stronger association between the dataset genes and the pathway.
<1B-3-75> Note: FDR stands for False Discovery Rate, a method used to correct for multiple testing.

<1B-3-76> The odds ratio quantifies the likelihood that the observed association between the dataset genes and a pathway is not due to random chance.

4. Draft a paragraph to describe the findings from the figure. This is one paragraph for the result part of a manuscript. 150 words or less.

<1B-3-77> In our exploration of the transcriptional response of RPMI8226 multiple myeloma cells (MMs) to bone marrow stromal cells (BMSCs), notable alterations in gene expression were observed (Figure X).
<1B-3-78> A distinct separation in the Principal Component Analysis (PCA) plot highlighted the profound transcriptional differences between MM cells co-cultured with BMSCs and those in monoculture, with the first two principal components accounting for 93% of the total variance.
<1B-3-79> Our volcano plot pinpointed genes like SOCS3 and JUNB as notably upregulated.
<1B-3-80> Pathway enrichment analysis further elucidated the biological implications of these transcriptional changes.
<1B-3-81> Key pathways, such as cytokine-cytokine receptor interaction, JAK-STAT signaling, and NOD-like receptor signaling, were found to be significantly enriched.
<1B-3-82> These findings underscore the pivotal role of the bone marrow microenvironment in influencing the gene expression landscape of MM cells, providing insights into potential therapeutic targets and the intricate interplay between tumor cells and their surroundings.

The “RNA” case study (in-depth assessment 1)

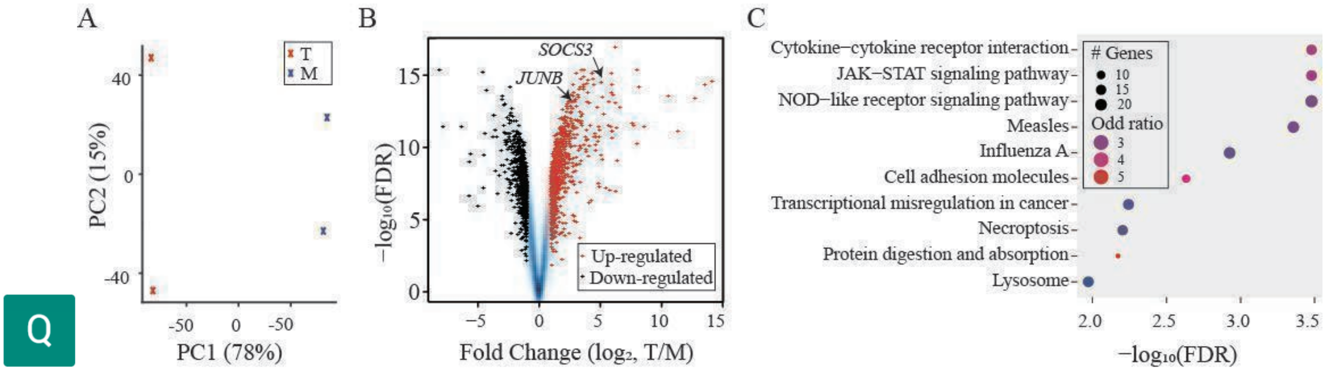

Q

Acting as a bioinformatics professional proficient in RNA-Seq data analysis, you are interpreting a figure to better appreciate the transcription response of multiple myeloma cells (MMs) to bone marrow stromal cells (BMSCs). You also have extensive laboratory research experience in the bone marrow tumor microenvironment of MM. In this task, the expression profile of MM cell line RMPI8226 in trans-well coculture (T) with a BMSC cell line was compared to monoculture (M). The attached figure illustrates the differential expression between the two conditions. Please do the following:

1. Provide an overview of each panel.

<1-1-1> Certainly!
<1-1-2> Based on the given figure, I will provide an overview of each panel:.
<1-1-3> Panel A: Principal Component Analysis (PCA)
<1-1-4> This panel showcases a PCA plot.
<1-1-5> PCA is a dimensionality reduction technique commonly used in bioinformatics to visualize the overall variance in large datasets.
<1-1-6> The X-axis represents Principal Component 1 (PC1) which captures 78% of the variance, while the Y-axis represents Principal Component 2 (PC2) which captures 15% of the variance.
<1-1-7> There are two sets of data points: marked with "T" (representing trans-well coculture) and "M" (representing monoculture).
<1-1-8> The distribution of these points shows how these two samples cluster or differ from each other based on the two most influential principal components.
<1-1-9> The distance between the T and M clusters suggests a significant variation in gene expression profiles between the coculture and monoculture conditions.
<1-1-10> Panel B: Volcano Plot
<1-1-11> A volcano plot is used to display the results of differential expression analyses.
<1-1-12> The X-axis shows the fold change (log2 transformed) between the two conditions, while the Y-axis displays the statistical significance (-log10 of the FDR, or false discovery rate).
<1-1-13> The red points represent genes that are up-regulated in the trans-well coculture condition (T) compared to the monoculture (M).
<1-1-14> The blue points indicate down-regulated genes. {Black not blue for down-regulated genes.}
<1-1-15> A few of the most significantly differentially expressed genes are labeled, including SOCS3 and JUNB.
<1-1-16> Genes located farther to the right and at the top are the most significantly up-regulated, while those to the left and at the top are the most significantly down-regulated.
<1-1-17> Panel C: Pathway Enrichment Analysis
<1-1-18> This panel appears to show results from a pathway enrichment analysis, which identifies biological pathways that are significantly impacted based on the differentially expressed genes.
<1-1-19> The X-axis displays the -log10 of the FDR, indicating the significance of the enrichment.
<1-1-20> The higher the value, the more significant the pathway enrichment.
<1-1-21> The size of the dots corresponds to the number of genes from the dataset that fall into each pathway, as shown in

the legend (Genes).
<1-1-22> The color of the dots represents the odd ratio, which is a measure of the likelihood that the observed enrichment for a particular pathway is not by chance.
<1-1-23> Several pathways, like "Cytokine-cytokine receptor interaction" and "JAK-STAT signaling pathway," appear to be highly enriched, suggesting they may play crucial roles in the transcriptional response of MMs to BMSCs.
<1-1-24> In summary, this figure provides a comprehensive overview of the transcriptional changes in multiple myeloma cells when co-cultured with bone marrow stromal cells, highlighting key genes and pathways affected.

2. For panel A, what do the numbers in parentheses mean?

<1-1-25> In Panel A, the numbers in parentheses next to PC1 (78%) and PC2 (15%) represent the percentage of variance explained by each principal component.
<1-1-26> In the context of Principal Component Analysis (PCA):.
<1-1-27> PC1 (78%): This means that the first principal component (PC1) accounts for 78% of the total variance in the dataset.
<1-1-28> It captures the primary direction in which the data varies.
<1-1-29> PC2 (15%): The second principal component (PC2) accounts for 15% of the total variance.
<1-1-30> This is the second most significant direction of variance in the data, and it is orthogonal (perpendicular) to PC1.
<1-1-31> Together, these two components (PC1 and PC2) capture a cumulative 93% of the total variance in the data, allowing for a 2D visualization that retains a significant portion of the information from the original dataset.
<1-1-32> The percentages help researchers quickly gauge how well the PCA plot represents the overall data.

3. For panel B, the positions of SOCS3 and JUNB are indicated by arrowheads. Estimate the values from the x-axis for the two genes. Are the two genes up-regulated or down-regulated? Based on your findings in the literature, why are the two genes chosen and are these results expected?

<1-1-33> Panel B Interpretation:.
<1-1-34> SOCS3 and JUNB positions: Based on the provided volcano plot:.
<1-1-35> SOCS3:
<1-1-36> The value on the x-axis for SOCS3 seems to be around 5, indicating a positive log2 fold change.
<1-1-37> JUNB:
<1-1-38> The value on the x-axis for JUNB seems to be around 6 or slightly higher, again indicating a positive log2 fold change. {Fold change incorrect. Should be around 3.}
<1-1-39> Up-regulation or Down-regulation:
<1-1-40> Both SOCS3 and JUNB are located on the right side of the volcano plot (with positive fold change values), indicating they are up-regulated in the trans-well coculture condition (T) relative to the monoculture condition (M).
<1-1-41> Literature-Based Findings:.
<1-1-42> SOCS3:
<1-1-43> The Suppressor of Cytokine Signaling 3 (SOCS3) gene is often involved in negative regulation of cytokine signaling pathways.
<1-1-44> Its up-regulation suggests a potential negative feedback response to cytokine signaling, which might be activated due to the interaction between MM cells and BMSCs.
<1-1-45> In the context of multiple myeloma, increased SOCS3 expression has been reported in response to various stimuli and might be related to disease progression or drug resistance, although the specific context can vary.
<1-1-46> JUNB:
<1-1-47> JUNB is a member of the AP-1 transcription factor family and plays roles in cell proliferation, differentiation, and apoptosis.

<1-1-48> Changes in JUNB expression have implications for tumor cell growth and survival.
<1-1-49> In the context of MM, the up-regulation of JUNB might reflect changes in the tumor microenvironment, cell stress, or interactions with surrounding stromal cells.
<1-1-50> Given the roles of these genes in cell signaling, immune response modulation, and tumor progression, it makes sense that they might be highlighted in a study investigating the transcriptional response of MM cells to BMSCs.
<1-1-51> However, to definitively say whether these results are expected, one would need to consult specific literature on MM, the tumor microenvironment of MM, and the interactions between MM cells and BMSCs.

Q

4. For panel C, pick the top two pathways and explain the criteria. It is not indicated on the plot whether upregulated or downregulated genes were used for the pathway enrichment analysis. However, by correlating the findings to existing knowledge in the literature, which group of genes were likely used?

<1-1-52> Panel C Interpretation:. Top Two Pathways and Criteria:
<1-1-53> The top two pathways, based on the X-axis (which represents -log10 of the FDR, indicating the significance of enrichment), appear to be:. 1. Cytokine-cytokine receptor interaction 2. JAK-STAT signaling pathway.
<1-1-54> The criteria for selecting these pathways as significant seem to be based on the -log10(FDR) values; the farther the pathway is to the right on the X-axis, the more significant the enrichment.
<1-1-55> The size of the dots represents the number of genes from the dataset that are involved in each pathway, while the color indicates the odd ratio, showing the likelihood that the observed enrichment is not by chance.
<1-1-56> Correlating Findings to Literature and Inferring Upregulated vs. Downregulated:.
<1-1-57> Cytokine-cytokine receptor interaction:
<1-1-58> This pathway is central to cell communication, especially in the immune system.
<1-1-59> Given that bone marrow stromal cells (BMSCs) and multiple myeloma cells (MMs) engage in extensive cross-talk through cytokines and their receptors, it's conceivable that genes involved in this pathway could be affected when MM cells are co-cultured with BMSCs.
<1-1-60> Cytokines can promote tumor cell growth, drug resistance, and immune evasion in MM.
<1-1-61> JAK-STAT signaling pathway:
<1-1-62> This is a major intracellular signaling pathway activated by cytokines.
<1-1-63> Activation of the JAK-STAT pathway can lead to cell proliferation, survival, and differentiation.
<1-1-64> In MM, aberrant activation of the JAK-STAT pathway has been linked to disease progression, and it's a potential therapeutic target.
<1-1-65> Given that both pathways are typically activated in response to cytokine signaling and that cytokines are often upregulated during cell-cell interactions (like the interaction between MM cells and BMSCs), it's likely that the upregulated genes were used for the pathway enrichment analysis.
<1-1-66> This inference is further supported by the pathways' roles in promoting tumor growth and survival, which would be consistent with upregulated gene expression in the co-culture setting.

Q

5. Are there any specific suggestions to improve the data presentation of the figure?

<1-1-67> Certainly!
<1-1-68> While the figure provides valuable insights, there are always potential improvements that can enhance clarity and interpretation.
<1-1-69> Here are some suggestions for improving the data presentation of the figure:.
<1-1-70> Panel A (PCA Plot): 1. Legends and Labels:
<1-1-71> The legend differentiating "T" from "M" can be made more prominent.
<1-1-72> Additionally, clearer labels indicating what "T" and "M" represent might help readers who aren't familiar with the experimental setup.

<1-1-73> 2. Variability:
<1-1-74> While PC1 and PC2 are shown, adding a brief note or inset about the contributions of subsequent principal components (like PC3, PC4, etc.) might provide context about the data's complexity.
<1-1-75> 3. Points' Design:
<1-1-76> Consider using different shapes or slightly larger markers for the data points to make them more distinguishable.
<1-1-77> Panel B (Volcano Plot): 1.
<1-1-78> Threshold Lines:
<1-1-79> Adding horizontal lines to indicate significance thresholds (e.g., a specific FDR value) and vertical lines for fold change cutoffs (e.g., log2 fold change > 2 or < -2) can help readers quickly identify genes of interest.
<1-1-80> 2. Zoomed Inset:
<1-1-81> For densely populated areas, consider including a zoomed-in inset to show more details or highlight additional key genes.
<1-1-82> 3. Interactive Features:
<1-1-83> If presented digitally, interactive features allowing users to hover over points for gene names or exact values can be invaluable.
<1-1-84> Panel C (Pathway Enrichment Analysis): 1. Clearer Labels:
<1-1-85> Space permitting, the entire pathway name could be written out, or font size increased for better legibility. {Pathway name has been written out in the plot}
<1-1-86> 2. Color Gradient: Use a continuous color gradient for the odd ratio to provide more nuance between pathways.
<1-1-87> 3. Additional Information:
<1-1-88> If the pathway analysis tool used allows, pathway impact scores or other measures could be shown to provide more depth about the significance of each pathway.
<1-1-89> 4. Bar Plot Option:
<1-1-90> Sometimes, a horizontal bar plot with pathways on the Y-axis and -log10(FDR) on the X-axis can be an alternative and clearer representation, especially when dealing with a large number of pathways.
<1-1-91> General Suggestions: 1. Consistent Font Size:
<1-1-92> Ensure that font sizes are consistent across all panels for labels, legends, and axis titles.
<1-1-93> 2. Colorblind Friendly: Use colorblind-friendly palettes to ensure accessibility for all readers.
<1-1-94> 3. Unified Legend:
<1-1-95> If certain symbols, colors, or annotations are used across multiple panels, consider a unified legend for the entire figure.
<1-1-96> Finally, a brief figure caption or description providing context about the experiment and what each panel represents is crucial for readers who might not have the background or are skimming through the document.

Q

6. Draft a title and figure legend. Make sure to include details.

<1-1-97> Title: "Differential Gene Expression and Pathway Enrichment in RMPI8226 Multiple Myeloma Cells Co-cultured with Bone Marrow Stromal Cells".
<1-1-98> Figure Legend:.
<1-1-99> Figure X: Transcriptional response of the RMPI8226 multiple myeloma cell line during trans-well coculture with a bone marrow stromal cell (BMSC) line compared to monoculture conditions.
<1-1-100> A. Principal Component Analysis (PCA): The plot visualizes the variance in gene expression profiles between monoculture (M, blue crosses) and trans-well coculture (T, red stars).
<1-1-101> PC1 and PC2 represent the first and second principal components, accounting for 78% and 15% of the total variance, respectively.
<1-1-102> B. Volcano Plot: Displays the differential gene expression between the two conditions.
<1-1-103> The X-axis shows log2 fold change, with positive values indicating upregulation in the coculture condition.
<1-1-104> The Y-axis represents the -log10 of the false discovery rate (FDR), indicating statistical significance.
<1-1-105> Key genes, including SOCS3 and JUNB, are highlighted and labeled.
<1-1-106> C. Pathway Enrichment Analysis: Shows significantly impacted biological pathways based on differentially expressed genes.

<1-1-107> Pathways are plotted against the -log10(FDR) for significance.
<1-1-108> Dot size corresponds to the number of genes in the dataset associated with each pathway, and color indicates the odd ratio, denoting the likelihood of observed enrichment occurring by chance.
<1-1-109> All experiments were conducted in triplicate, and data are representative of three independent experiments. {Duplicates not triplicates}

Q

7. Draft a paragraph to describe the findings from the figure. This is one paragraph for the result part of a manuscript. 150 words or less.

<1-1-110> In the examination of the transcriptional changes of RMPI8226 multiple myeloma cells (MMs) when co-cultured with bone marrow stromal cells (BMSCs), distinct variations were observed.
<1-1-111> The PCA plot revealed substantial divergence between monoculture and co-culture conditions, with PC1 and PC2 capturing 78% and 15% of the variance, respectively (Panel A).
<1-1-112> Notably, SOCS3 and JUNB were significantly up-regulated in the co-culture environment, as evidenced by the volcano plot (Panel B).
<1-1-113> Further pathway enrichment analysis pinpointed the "Cytokine-cytokine receptor interaction" and "JAK-STAT signaling pathway" as the most affected pathways, suggesting an intensified cytokine signaling and response in MMs during interaction with BMSCs (Panel C).
<1-1-114> Collectively, these findings highlight the pronounced transcriptional reshaping in MMs influenced by the bone marrow microenvironment, underscoring the significance of BMSC-MM interplay in myeloma progression.

The “RNA” case study (in-depth assessment 2)

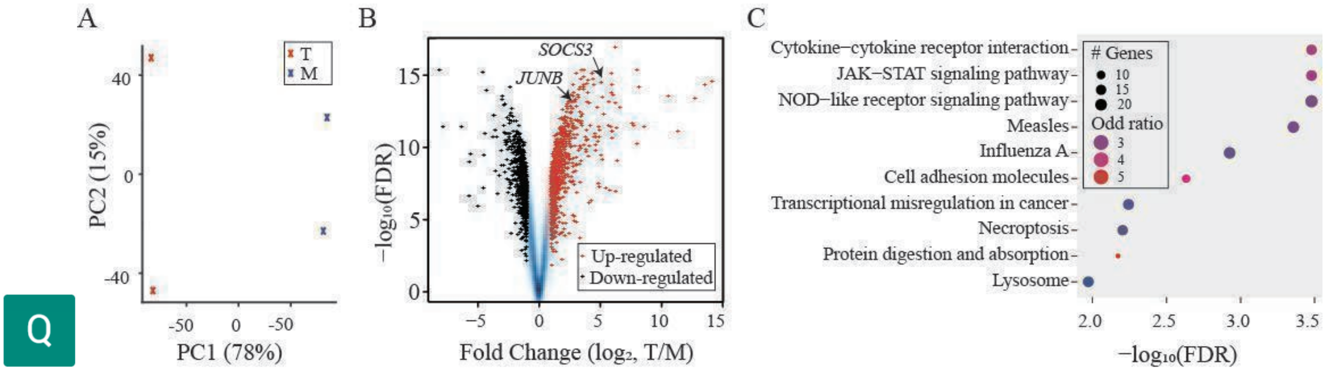

Q

Acting as a bioinformatics professional proficient in RNA-Seq data analysis, you are interpreting a figure to better appreciate the transcription response of multiple myeloma cells (MMs) to bone marrow stromal cells (BMSCs). You also have extensive laboratory research experience in the bone marrow tumor microenvironment of MM. In this task, the expression profile of MM cell line RMPI8226 in trans-well coculture (T) with a BMSC cell line was compared to monoculture (M). The attached figure illustrates the differential expression between the two conditions. Please do the following:

1. Provide an overview of each panel.

<1-2-1> Certainly!
<1-2-2> Let's go through each panel:.
<1-2-3> Panel A: Title: Principal Component Analysis (PCA) of Transcriptional Profiles.
<1-2-4> Description:
<1-2-5> This panel showcases the PCA plot, which is a common method used to reduce dimensionality and visualize the variation in large datasets.
<1-2-6> The x-axis represents the first principal component (PC1) accounting for 78% of the variance, and the y-axis represents the second principal component (PC2) accounting for 15% of the variance.
<1-2-7> Observation:
<1-2-8> Two distinct clusters are evident.
<1-2-9> The blue cluster represents MM cells in trans-well coculture (T) with BMSCs, while the red cluster represents MM cells in monoculture (M). {Red is for T and blue is for M}
<1-2-10> The clear separation indicates significant transcriptional differences between the two conditions.
<1-2-11> Panel B: Title: Volcano Plot of Differentially Expressed Genes.
<1-2-12> Description:
<1-2-13> The volcano plot visually displays differential gene expression between the two conditions.
<1-2-14> The x-axis represents the fold change in expression (log2 scale of T/M), where positive values indicate up-regulation in coculture and negative values indicate down-regulation.
<1-2-15> The y-axis is the negative logarithm (base 10) of the false discovery rate (FDR), which corrects for multiple testing; higher values mean more statistical significance.
<1-2-16> Observation:
<1-2-17> The red points represent genes that are up-regulated in coculture, while the blue points represent down-regulated genes. {Black not blue for down-regulated genes.}
<1-2-18> Two genes, SOCS3 and JUNB, are labeled, suggesting they are of particular interest, possibly due to their high fold-change and/or statistical significance.
<1-2-19> Panel C: Title: Pathway Enrichment Analysis.
<1-2-20> Description:
<1-2-21> This panel shows the results of a pathway enrichment analysis.
<1-2-22> The x-axis represents the negative logarithm (base 10) of the FDR, indicating the significance of enrichment.
<1-2-23> The y-axis lists various biological pathways.

<1-2-24> The size of the dot corresponds to the number of genes in the dataset associated with that pathway, and the color indicates the odd ratio, which signifies the likelihood of observing the given number of genes in that pathway by chance.
<1-2-25> Observation:
<1-2-26> Several pathways are enriched in the dataset, suggesting their potential relevance to the response of MM cells to BMSCs.
<1-2-27> For instance, "Cytokine-cytokine receptor interaction" and "JAK-STAT signaling pathway" appear significant, indicating a possible involvement of these pathways in the interaction between MM cells and BMSCs.
<1-2-28> In summary, the figure provides comprehensive insights into the transcriptional response of MM cells when cocultured with BMSCs.
<1-2-29> The distinct clustering in PCA, differentially expressed genes in the volcano plot, and the enriched pathways collectively highlight the profound influence of BMSCs on the MM cell line's gene expression.

Q

2. For panel A, what do the numbers in parentheses mean?

<1-2-30> In Panel A, the numbers in parentheses next to PC1 and PC2 denote the proportion of the total variance in the dataset that is captured by each respective principal component.
<1-2-31> Specifically:.
<1-2-32> PC1 (78%): This means that the first principal component (PC1) captures 78% of the total variance in the dataset.
<1-2-33> PC2 (15%):
<1-2-34> Similarly, the second principal component (PC2) captures 15% of the total variance.
<1-2-35> In other words, the two dimensions shown in the PCA plot (PC1 and PC2) together account for 93% (78% + 15%) of the total variability in the gene expression data.
<1-2-36> The higher the percentage, the more variation that particular principal component captures, and thus it's more informative for differentiating between different conditions or groups in the dataset.

Q

3. For panel B, the positions of SOCS3 and JUNB are indicated by arrowheads. Estimate the values from the x-axis for the two genes. Are the two genes up-regulated or down-regulated? Based on your findings in the literature, why are the two genes chosen and are these results expected?

<1-2-37> Panel B Analysis:.
<1-2-38> Estimating Values: SOCS3:
<1-2-39> This gene appears to have a fold change value (from the x-axis) of slightly above 5.
<1-2-40> JUNB:
<1-2-41> This gene's fold change value seems to be around 6 or slightly above. {JUNB FC is 3 not 6}
<1-2-42> Up-regulation or Down-regulation:
<1-2-43> Both SOCS3 and JUNB are on the right side of the 0 value on the x-axis, which means both genes have a positive fold change.
<1-2-44> Hence, both SOCS3 and JUNB are up-regulated in the trans-well coculture (T) compared to the monoculture (M).
<1-2-45> Literature-based Insights: SOCS3 (Suppressor of Cytokine Signaling 3): Role:
<1-2-46> SOCS3 is known to play a crucial role in the negative regulation of cytokine signaling.
<1-2-47> It's part of a classical feedback mechanism to prevent excessive activation of JAK-STAT signaling, which can be initiated by various cytokines.
<1-2-48> Expected Result:
<1-2-49> Given that the interaction between MM cells and BMSCs may stimulate cytokine production or response, an up-regulation of SOCS3 could be anticipated as a regulatory response to keep the cytokine signaling in check.

<1-2-50> JUNB:
<1-2-51> Role:
<1-2-52> JUNB is a member of the AP-1 transcription factor complex and is involved in various cellular processes, including cell growth, differentiation, and apoptosis.
<1-2-53> Its role in the bone marrow environment has been associated with cellular responses to external stimuli, including cytokines.
<1-2-54> Expected Result: The up-regulation of JUNB in MM cells when in coculture with BMSCs might be reflective of altered cellular states or responses to the signaling molecules secreted by BMSCs.
<1-2-55> Conclusion: It's likely that both SOCS3 and JUNB were highlighted due to their known roles in cytokine signaling and cellular responses in the bone marrow environment, respectively.
<1-2-56> The up-regulation of these genes suggests a pronounced influence of BMSCs on the MM cell line's transcriptional landscape, which could be in line with existing literature on the role of the bone marrow microenvironment in MM progression.
<1-2-57> However, to get a full picture, one would need to delve deeper into specific research articles and studies focused on these genes in the context of MM and BMSC interactions.

Q

4. For panel C, pick the top two pathways and explain the criteria. It is not indicated on the plot whether upregulated or downregulated genes were used for the pathway enrichment analysis. However, by correlating the findings to existing knowledge in the literature, which group of genes were likely used?

<1-2-58> Panel C Analysis:.
<1-2-59> Top Two Pathways: 1. Cytokine-cytokine receptor interaction:
<1-2-60> This pathway has the most significant enrichment, as indicated by the farthest value on the x-axis.
<1-2-61> 2. JAK-STAT signaling pathway:
<1-2-62> This is the second most enriched pathway, given its position on the x-axis.
<1-2-63> Criteria for Selection:
<1-2-64> The significance of enrichment for each pathway is represented by the negative logarithm (base 10) of the FDR on the x-axis.
<1-2-65> A pathway with a higher value on this axis indicates stronger evidence against the null hypothesis (i.e., that the observed gene set overlap occurred by chance), meaning the pathway is more significantly enriched in the dataset.
<1-2-66> The size of the dot corresponds to the number of genes in the dataset associated with that pathway, while the color indicates the odds ratio.
<1-2-67> Inference from Existing Knowledge:.
<1-2-68> Cytokine-cytokine receptor interaction:
<1-2-69> This pathway is central to cell signaling, especially in the context of immune responses and inflammation.
<1-2-70> Given the bone marrow microenvironment's rich cytokine milieu and the interaction between MM cells and BMSCs, one would expect significant changes in genes associated with cytokine signaling.
<1-2-71> JAK-STAT signaling pathway:
<1-2-72> This pathway is directly linked with cytokine signaling.
<1-2-73> When cytokines bind to their receptors, they often activate the JAK-STAT signaling cascade, leading to the transcriptional activation of target genes.
<1-2-74> Dysregulation of JAK-STAT signaling is implicated in various diseases, including cancers.
<1-2-75> Given that both of these pathways play crucial roles in cell signaling, and considering the transcriptional differences seen in Panel B, it's likely that the up-regulated genes were used for the pathway enrichment analysis.
<1-2-76> This assumption is further supported by the fact that interactions with BMSCs would typically stimulate MM cells, potentially leading to the up-regulation of genes associated with signaling pathways like those mentioned.
<1-2-77> Conclusion: The top two pathways from Panel C are central to cytokine signaling and cellular responses.
<1-2-78> Correlating with existing knowledge, the significant enrichment of these pathways suggests that up-regulated genes in MM cells (when cocultured with BMSCs) were likely used for the pathway enrichment analysis, emphasizing the activated signaling and interactive responses of MM cells in the presence of BMSCs.

5. Are there any specific suggestions to improve the data presentation of the figure?

<1-2-79> Certainly, while the figure provides a wealth of information, there are some improvements that could be made to enhance clarity and comprehensiveness:.
<1-2-80> 1. Panel A (PCA Plot): Legends: Introducing a legend box might make it clearer.
<1-2-81> While 'T' and 'M' are annotated on the plot, a legend box would give a clear demarcation between coculture and monoculture samples.
<1-2-82> Percent Variance:
<1-2-83> It could be helpful to add a third principal component (PC3) if there's any additional meaningful variance.
<1-2-84> This might provide further insights into the dataset's structure.
<1-2-85> 2. Panel B (Volcano Plot): Threshold Lines:
<1-2-86> Adding horizontal lines to indicate the significance threshold (e.g., FDR < 0.05) and vertical lines for fold change cutoffs (e.g., log2 fold change > 2 or < -2) would provide a clearer visual cue for determining significant up and down-regulated genes.
<1-2-87> More Labels:
<1-2-88> While SOCS3 and JUNB are highlighted, it might be informative to label other extreme outliers, which could represent genes of interest.
<1-2-89> 3. Panel C (Pathway Enrichment Analysis): Color Scale:
<1-2-90> The color scale indicating odd ratios could benefit from a legend or color bar to allow for quantitative interpretation.
<1-2-91> Directionality:
<1-2-92> To provide clarity on whether the enriched pathways are a result of up-regulated or down-regulated genes, a separate color scheme or symbol (e.g., upward and downward triangles) could be utilized.
<1-2-93> Full Pathway Names:
<1-2-94> Ensure that pathway names aren't cut off or overlapped.
<1-2-95> This might involve adjusting text size, plot dimensions, or text positioning.
<1-2-96> 4. General Suggestions: Font Consistency: Ensure consistent font sizes and types across all panels.
<1-2-97> Uniform Color Palette:
<1-2-98> Adopt a consistent color scheme across panels for 'T' and 'M' conditions to avoid confusion.
<1-2-99> Figure Title:
<1-2-100> A title for the entire figure would offer immediate context to readers.
<1-2-101> Methodological Details:
<1-2-102> Beneath the figure, a brief note on the methods (e.g., statistical tests used, software/tools, sample size) could offer readers a quick understanding of the analysis performed.
<1-2-103> 5.
<1-2-104> Additional Plots: Heatmap:
<1-2-105> If space allows, a heatmap of the top differentially expressed genes could provide a deeper view into the expression patterns between the two conditions.
<1-2-106> Histogram or Box Plot:
<1-2-107> A representation of the overall distribution of fold changes or p-values might help readers gauge the overall trends in the data.
<1-2-108> These suggestions aim to offer a more comprehensive, clearer, and user-friendly presentation of the data while maintaining the scientific integrity of the findings.

6. Draft a title and figure legend. Make sure to include details.

<1-2-109> Title: Transcriptional Response of Multiple Myeloma Cells to Bone Marrow Stromal Cell Coculture: Differential

Gene Expression and Pathway Enrichment Analysis.
<1-2-110> Figure Legend:.
<1-2-111> Figure X.
<1-2-112> Differential gene expression and associated biological pathways in RMPI8226 MM cells under trans-well coculture with BMSCs.
<1-2-113> A. Principal Component Analysis (PCA): Plot represents the transcriptional variability between MM cells in monoculture (M, in red) and trans-well coculture with BMSCs (T, in blue). {M in blue, T in red. Not the opposite.}
<1-2-114> PC1 accounts for 78% of the total variance, while PC2 accounts for 15%.
<1-2-115> B. Volcano Plot of Gene Expression:
<1-2-116> Differential expression of genes is plotted with fold change (log2, T/M) on the x-axis and statistical significance (-log10 FDR) on the y-axis.
<1-2-117> Genes up-regulated in coculture are shown in red, while down-regulated genes are in blue. {Should be black}
<1-2-118> Notable genes, SOCS3 and JUNB, are highlighted and labeled.
<1-2-119> C. Pathway Enrichment Analysis:
<1-2-120> Biological pathways enriched in the differentially expressed gene set.
<1-2-121> The x-axis indicates the statistical significance of enrichment (-log10 FDR), while the y-axis lists various enriched biological pathways.
<1-2-122> Dot size corresponds to the number of associated genes in the dataset, and color indicates the odds ratio, with warmer colors suggesting higher likelihoods of the observed gene set overlap.
<1-2-123> Methods:
<1-2-124> Gene expression profiles from RMPI8226 MM cells, either in monoculture or coculture with BMSCs, were obtained using RNA-Seq.
<1-2-125> Differential expression was determined using [specific software/tool], with an FDR-adjusted p-value < 0.05 considered statistically significant.
<1-2-126> Pathway enrichment was conducted using [specific tool/software], focusing on the [up-regulated/down-regulated] gene set.
<1-2-127> Note: Exact methods and software/tools should be filled in based on the actual methodologies used in the study.

Q

7. Draft a paragraph to describe the findings from the figure. This is one paragraph for the result part of a manuscript. 150 words or less.

<1-2-128> In our examination of the transcriptional interplay between RMPI8226 MM cells and BMSCs, clear distinctions in gene expression profiles were observed (Figure X).
<1-2-129> Principal Component Analysis revealed two discrete clusters, highlighting pronounced transcriptional variations between monoculture and trans-well coculture conditions, with the latter capturing a combined variance of 93% (PC1: 78%, PC2: 15%). {The explanation of coculture condition capturing a combined variance of 93% makes no sense. It is PC1 explained 78% variance and PC1 explained 15%.}
<1-2-130> A subsequent volcano plot elucidated differential gene expression patterns, notably showcasing the up-regulation of genes SOCS3 and JUNB in coculture.
<1-2-131> Delving deeper, pathway enrichment analysis underscored the pertinence of "Cytokine-cytokine receptor interaction" and "JAK-STAT signaling pathway", both of which exhibited significant enrichment.
<1-2-132> These pathways, pivotal in cytokine signaling and cellular interactions, suggest a heightened responsiveness of MM cells to the stimulatory environment provided by BMSCs.
<1-2-133> Collectively, our findings illuminate the intricate transcriptional alterations in MM cells modulated by the presence of BMSCs, spotlighting potential molecular players and pathways in this dynamic interaction.

The “RNA” case study (in-depth assessment 3)

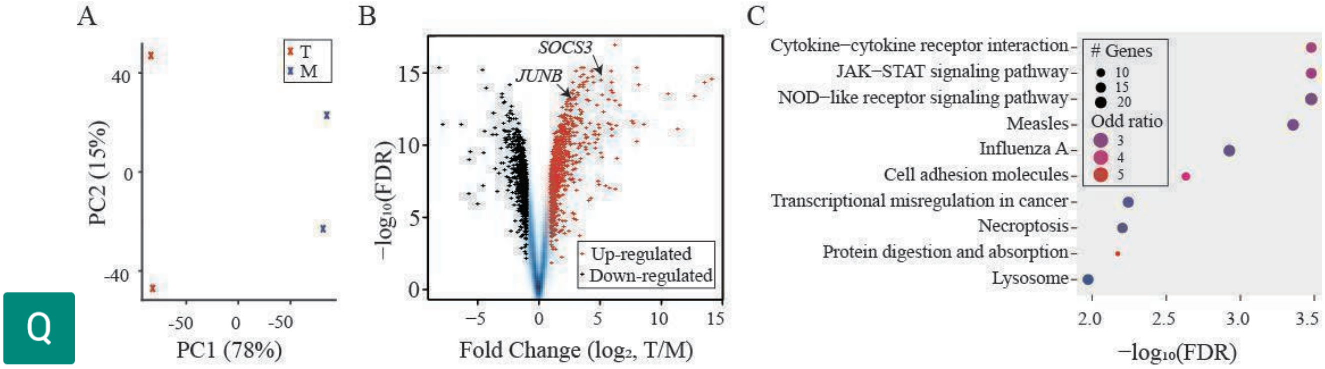

Acting as a bioinformatics professional proficient in RNA-Seq data analysis, you are interpreting a figure to better appreciate the transcription response of multiple myeloma cells (MMs) to bone marrow stromal cells (BMSCs). You also have extensive laboratory research experience in the bone marrow tumor microenvironment of MM. In this task, the expression profile of MM cell line RMPI8226 in trans-well coculture (T) with a BMSC cell line was compared to monoculture (M). The attached figure illustrates the differential expression between the two conditions. Please do the following:

1. Provide an overview of each panel.

<1-3-1> Of course, let's break down the attached figure panel by panel.
<1-3-2> Panel A:
<1-3-3> This panel depicts a principal component analysis (PCA) plot.
<1-3-4> PCA is a dimension-reduction tool that is commonly used in RNA-Seq analysis to visualize the overall variance in gene expression data.
<1-3-5> X-axis (PC1, 78%): Represents the first principal component which captures the highest variance (78%) in the dataset.
<1-3-6> Y-axis (PC2, 15%): Represents the second principal component which captures the second highest variance (15%) in the dataset.
<1-3-7> Blue Crosses (M): Represent the monoculture condition (M) of the RMPI8226 MM cell line.
<1-3-8> Red Stars (T): Represent the trans-well coculture condition (T) with the BMSC cell line.
<1-3-9> The distance between the points provides an indication of the difference in gene expression profiles.
<1-3-10> Here, the two conditions (M and T) form distinct clusters, suggesting a significant difference in their gene expression profiles.
<1-3-11> Panel B: This is a volcano plot, a commonly used visualization in differential gene expression analysis.
<1-3-12> X-
<1-3-13> axis (Fold Change (log2, T/M)): Represents the log2 transformed fold change in gene expression between the trans-well coculture (T) and monoculture (M) conditions.
<1-3-14> A value >0 indicates up-regulation in the T condition, while a value <0 indicates down-regulation.
<1-3-15> Y-axis (-log10(FDR)): Represents the -log10 transformed False Discovery Rate (FDR).
<1-3-16> Higher values indicate greater significance in differential expression.
<1-3-17> Blue Dots: Represent down-regulated genes in the T condition as compared to M. {Black but not blue for down-regulated genes}
<1-3-18> Red Dots: Represent up-regulated genes in the T condition as compared to M.
<1-3-19> Black Dots: Represent genes that are not significantly differentially expressed. {Blue but not black for no change genes}
<1-3-20> Highlighted Genes (e.g., SOCS3, JUNB): These are specific genes that have been labeled, probably because they have a known biological significance or are of particular interest in the study.
<1-3-21> Panel C: This panel represents a pathway enrichment analysis, which identifies biological pathways that are over-represented in the set of differentially expressed genes.

<1-3-22> X-axis (-log10(FDR)): Represents the -log10 transformed False Discovery Rate (FDR) for the enrichment of each pathway.
<1-3-23> More significant pathways (lower FDR values) are further to the right.
<1-3-24> Y-axis (Pathways): Lists various biological pathways.
<1-3-25> Dot Size (Genes): The size of the dots indicates the number of genes from the differential expression analysis that fall into each pathway.
<1-3-26> Bigger dots mean more genes.
<1-3-27> Dot Color (Odd ratio): The color of the dots represents the odds ratio, a measure of the strength of association between the presence of differential expression and each pathway.
<1-3-28> Higher odds ratios are represented with deeper colors, indicating that the pathway is more likely to be affected by the changes in gene expression between T and M conditions.
<1-3-29> From the overview, it's evident that interaction with BMSCs in the bone marrow microenvironment leads to notable changes in gene expression in MM cells, affecting various pathways that might be critical in MM pathology or progression.

Q

2. For panel A, what do the numbers in parentheses mean?

<1-3-30> In Panel A, the numbers in parentheses next to PC1 and PC2 represent the percentage of variance explained by the respective principal components.
<1-3-31> PC1 (78%):
<1-3-32> The first principal component (PC1) captures 78% of the total variance in the dataset.
<1-3-33> This means that PC1 alone accounts for 78% of the observed differences in gene expression between the samples.
<1-3-34> PC2 (15%): The second principal component (PC2) captures 15% of the total variance in the dataset.
<1-3-35> Together, PC1 and PC2 account for 93% (78% + 15%) of the total variability in the data.
<1-3-36> These percentages help researchers understand how well the reduced dimensionality (in this case, two dimensions) represents the original high-dimensional data (i.e., expression levels of thousands of genes).
<1-3-37> In this case, a large portion of the variability in the data is captured by just these two components, making the PCA plot a meaningful visualization of the differences between the samples.

Q

3. For panel B, the positions of SOCS3 and JUNB are indicated by arrowheads. Estimate the values from the x-axis for the two genes. Are the two genes up-regulated or down-regulated? Based on your findings in the literature, why are the two genes chosen and are these results expected?

<1-3-38> Panel B Analysis:. 1. Estimation of Values for SOCS3 and JUNB:
<1-3-39> SOCS3: Appears to be located at approximately a value of 5 or slightly more on the x-axis.
<1-3-40> JUNB:
<1-3-41> Seems to be situated around the value of 7 or slightly above on the x-axis. {3 other than 7 for JUNB}
<1-3-42> 2. Up-regulated or Down-regulated:
<1-3-43> Since the x-axis represents the log2-transformed fold change in gene expression between the trans-well coculture (T) and monoculture (M) conditions, values greater than 0 indicate up-regulation in the T condition.
<1-3-44> Given that both SOCS3 and JUNB have positive values on the x-axis, we can conclude that both genes are up-regulated in the coculture condition (T) compared to the monoculture (M).
<1-3-45> Literature Insights on SOCS3 and JUNB:.
<1-3-46> SOCS3 (Suppressor of Cytokine Signaling 3): Function:
<1-3-47> It is known to be a negative regulator of cytokine signaling and can inhibit the JAK-STAT signaling pathway, which is critical in cytokine-mediated cellular responses.
<1-3-48> Relevance to MM:

<1-3-49> The bone marrow microenvironment in MM is rich in cytokines, which play a role in promoting MM cell growth, survival, and drug resistance.
<1-3-50> An up-regulation of SOCS3 might be an attempt of MM cells to regulate or counteract excessive cytokine signaling.
<1-3-51> Expected Results:
<1-3-52> The up-regulation of SOCS3 in the presence of BMSCs might be anticipated given the cytokine-rich environment and the role of SOCS3 in negative feedback regulation of cytokine responses.
<1-3-53> JUNB (Jun B proto-oncogene): Function:
<1-3-54> JUNB is a component of the AP-1 transcription factor complex and is involved in a variety of cellular processes, including proliferation, differentiation, and apoptosis.
<1-3-55> Relevance to MM:
<1-3-56> Changes in JUNB expression can influence the behavior of cancer cells, including MM.
<1-3-57> In some contexts, JUNB acts as a tumor suppressor, while in others, it may have oncogenic roles.
<1-3-58> Expected Results:
<1-3-59> Given the multifaceted role of JUNB in cancer biology, its up-regulation in the presence of BMSCs suggests that BMSCs might be influencing MM cell behavior, possibly driving proliferation or altering differentiation status.
<1-3-60> The exact role would need to be validated with further experiments, but its selection indicates its potential importance in the MM and BMSC interaction.
<1-3-61> In conclusion, both genes, SOCS3 and JUNB, have been chosen for their known roles in cellular signaling and cancer biology.
<1-3-62> Their up-regulation in the coculture condition suggests they might be key players in the interaction between MM cells and BMSCs.
<1-3-63> The observed results are consistent with what might be expected given the roles of these genes in similar cellular contexts.

Q

4. For panel C, pick the top two pathways and explain the criteria. It is not indicated on the plot whether upregulated or downregulated genes were used for the pathway enrichment analysis. However, by correlating the findings to existing knowledge in the literature, which group of genes were likely used?

<1-3-64> Panel C Analysis:.
<1-3-65> Top Two Pathways: 1. Cytokine-Cytokine Receptor Interaction:
<1-3-66> This pathway is the top-most one on the y-axis.
<1-3-67> Its significance is given by its position on the x-axis, which has a very low FDR (as indicated by the -log10(FDR) value being farthest to the right).
<1-3-68> 2. JAK-STAT Signaling Pathway:
<1-3-69> This is the second pathway listed.
<1-3-70> Like the first pathway, it also shows a significant enrichment, as indicated by its position on the x-axis, though slightly less significant than the cytokine-cytokine receptor interaction.
<1-3-71> Criteria for Selecting the Top Two Pathways: 1. Significance:
<1-3-72> Based on the -log10(FDR) value on the x-axis, which measures the statistical significance of the enrichment of differentially expressed genes in a particular pathway.
<1-3-73> The farther to the right a dot is, the more significant the pathway enrichment.
<1-3-74> 2. Number of Genes:
<1-3-75> The size of the dot represents the number of differentially expressed genes that are associated with that pathway.
<1-3-76> Larger dots indicate more genes, meaning that pathway is highly represented in the list of differentially expressed genes.
<1-3-77> Correlation with Existing Knowledge:.
<1-3-78> Cytokine-Cytokine Receptor Interaction and JAK-STAT Signaling Pathway are closely linked.
<1-3-79> The JAK-STAT pathway is activated by cytokines and is essential for transmitting information from chemical signals outside the cell to the cell nucleus.
<1-3-80> Given that both pathways are significant, this suggests a high level of activity related to cytokine signaling in the

coculture of MM cells with BMSCs.
<1-3-81> Given the nature of these pathways: 1. Cytokine-Cytokine Receptor Interaction:
<1-3-82> It deals with interactions between different cytokines and their receptors.
<1-3-83> Cytokines are signaling molecules that play a significant role in the bone marrow microenvironment, influencing cell growth, differentiation, and immune responses.
<1-3-84> 2. JAK-STAT Signaling Pathway:
<1-3-85> A central pathway mediating the effects of cytokines.
<1-3-86> In the context of MM, the JAK-STAT pathway has been implicated in MM cell proliferation, survival, and resistance to apoptosis.
<1-3-87> Considering the emphasis of these pathways and their role in cell signaling, growth, and survival, it is likely that the up-regulated genes were used for the pathway enrichment analysis.
<1-3-88> This is because the interaction of MM cells with BMSCs would be expected to stimulate or activate signaling pathways that promote MM cell survival and growth in the bone marrow microenvironment.
<1-3-89> Thus, up-regulated genes in the coculture condition (as opposed to down-regulated ones) would be more indicative of the pathways that are being actively engaged in response to BMSC interaction.

Q

5. Are there any specific suggestions to improve the data presentation of the figure?

<1-3-90> Certainly, the presentation of scientific data is crucial for clear interpretation and communication.
<1-3-91> Here are some suggestions to improve the data presentation of the figure:.
<1-3-92> 1. Panel A (PCA Plot): Legend Expansion:
<1-3-93> The symbols for T and M might be obvious to those familiar with the experiment, but it would be beneficial to have a more detailed legend indicating what "T" and "M" stand for.
<1-3-94> Axes Labels:
<1-3-95> It might be helpful to provide more descriptive labels for the axes, such as "Principal Component 1 (78% Variance Explained)" instead of just "PC1 (78%)".
<1-3-96> 2. Panel B (Volcano Plot): Threshold Lines:
<1-3-97> Add horizontal dashed lines to indicate the significance threshold (commonly an FDR or p-value threshold).
<1-3-98> Similarly, vertical dashed lines can be added to indicate fold-change thresholds that were used to determine significance.
<1-3-99> Color Gradient or Size Gradient for Significance:
<1-3-100> Instead of just three colors, a gradient could represent the range of significance, giving a better idea of the genes that are just on the cusp of significance versus those that are highly significant.
<1-3-101> More Highlighted Genes:
<1-3-102> If there are other genes of importance or relevance to the study, they could also be labeled, not just SOCS3 and JUNB.
<1-3-103> 3.
<1-3-104> Panel C (Pathway Enrichment Analysis): Bar Plot Option:
<1-3-105> Instead of or in addition to the dot plot, a horizontal bar plot could be used to represent the -log10(FDR) values, making it easier to visually compare the significance of different pathways.
<1-3-106> Direction of Regulation:
<1-3-107> It might be helpful to use different symbols or colors for pathways enriched in up-regulated versus down-regulated genes.
<1-3-108> This would give the reader a quick understanding of whether the pathway is activated or inhibited in the coculture condition.
<1-3-109> Full Descriptions:
<1-3-110> Ensure that all pathway names are fully visible and not cut off or overlapped.
<1-3-111> 4.
<1-3-112> General Suggestions: Consistent Color Palette:
<1-3-113> Ensure that colors representing the same conditions (T and M or up-regulated and down-regulated) are consistent across all panels.

<1-3-114> Figure Title:
<1-3-115> Adding a concise title to the entire figure can provide immediate context to the viewer.
<1-3-116> Font Size & Clarity:
<1-3-117> Ensure that all labels, legends, and annotations are of a readable size and clear font.
<1-3-118> Methods Reference:
<1-3-119> If space allows, a brief note or reference pointing to the methods section for more details on the data processing and analysis can be helpful.
<1-3-120> Remember, the goal is to make the figure as informative and clear as possible, minimizing any potential confusion for readers who may not be intimately familiar with the experimental details or data analysis methods.

Q

6. Draft a title and figure legend. Make sure to include details.

<1-3-121> Certainly!
<1-3-122> Here's a draft title and figure legend for the provided figure:.
<1-3-123> Title: Differential Gene Expression and Pathway Enrichment Analysis of Multiple Myeloma Cells Co-cultured with Bone Marrow Stromal Cells.
<1-3-124> Figure Legend:.
<1-3-125> Panel A: Principal Component Analysis (PCA) of gene expression profiles.
<1-3-126> The plot depicts the first two principal components, PC1 and PC2, capturing 78% and 15% of the variance, respectively.
<1-3-127> Red stars represent MM cell line RMPI8226 in trans ell coculture with BMSCs (T), while blue crosses depict the monoculture condition (M).
<1-3-128> Panel B: Volcano plot illustrating the differential gene expression between the coculture (T) and monoculture (M) conditions.
<1-3-129> The x-axis shows the log2-transformed fold change (T/M), while the y-axis presents the -log10 transformed False Discovery Rate (FDR).
<1-3-130> Red dots denote up-regulated genes, blue dots signify down-regulated genes, and black dots represent genes with no significant differential expression. {Black for down and Blue for no change. Not the opposite.}
<1-3-131> Arrowheads highlight two genes of interest, SOCS3 and JUNB.
<1-3-132> Panel C: Pathway enrichment analysis of differentially expressed genes.
<1-3-133> Pathways are listed on the y-axis, and their enrichment significance is represented on the x-axis as -log10(FDR).
<1-3-134> Dot size corresponds to the number of genes from the differential expression analysis associated with each pathway, while dot color indicates the odds ratio of the association.
<1-3-135> This title and legend aim to provide a clear and concise summary of the figure while detailing the methods and findings.
<1-3-136> It can be adjusted based on any additional specifics or nuances of the study.

Q

7. Draft a paragraph to describe the findings from the figure. This is one paragraph for the result part of a manuscript. 150 words or less.

<1-3-137> In our investigation of the transcriptional response of multiple myeloma (MM) cells to bone marrow stromal cells (BMSCs), distinct gene expression profiles were observed between MM cells cocultured with BMSCs and those in monoculture, as evidenced by the pronounced separation in the PCA plot (Panel A).
<1-3-138> Notably, genes such as SOCS3 and JUNB were significantly up-regulated in the coculture condition (Panel B).
<1-3-139> Pathway enrichment analysis further illuminated the biological implications of these transcriptional changes (Panel C).
<1-3-140> The most pronounced pathways were the "Cytokine-cytokine Receptor Interaction" and the "JAK TAT Signaling

Pathway," both pivotal in mediating cellular responses in the tumor microenvironment.
<1-3-141> The prominence of these pathways, especially in the context of cytokine signaling, underlines the influential role of the BMSCs in shaping the MM cellular response and possibly driving disease progression or therapeutic resistance.
<1-3-142> This interplay between MM cells and their bone marrow milieu is crucial for understanding disease dynamics and potential therapeutic targets.

Supplementary Notes S2

Supplementary Notes S2: Parsed, indexed, and annotated statements for the "ZNF71" case study.

Instruction

Indexing principle: The indexing format adopted is <Case-Replicate-Statement>. In this format, "Case" represents the case study number, with 1 for "RNA", 2 for "ZNF71", 3 for "Clonal", and 4 for "YY1". The term "Replicate" indicates the replicate number, which can be either 1, 2, or 3. "Statement" corresponds to the statement's sequential position within a specific replicate of a case study. For example, the third statement (3) in the second replicate (2) of the "RNA" case study (1) is indexed as <1-2-3>. Furthermore, a letter "B" is appended to the "Case" slot if the chat history originates from prompts associated with the basal assessment model.

Color-coding: True statements in **blue**, false ones in **red** (with errors highlighted in **yellow**), and chitchats (non-informative statements) in **black**, which are excluded from evaluation. An {explanation} highlighted in yellow in a curly bracket would append to the end of the false statement.

* indicate statements where the only mistake is from mis-spelling of gene name, CD27 as CD271 abd IKBKB as IKKBK.

Overall performance

For the calculation of overall performance, a statement is marked as true if the contents for all its indicated categories are correct.

Replicate	Number of true statements	Number of evaluated statements	Accuracy
ZNF71_rep1	29	45	64.44%
ZNF71_rep2	31	48	64.58%
ZNF71_rep3	27	42	64.29%

The “ZNF71” case study (in-depth assessment 1)

Q

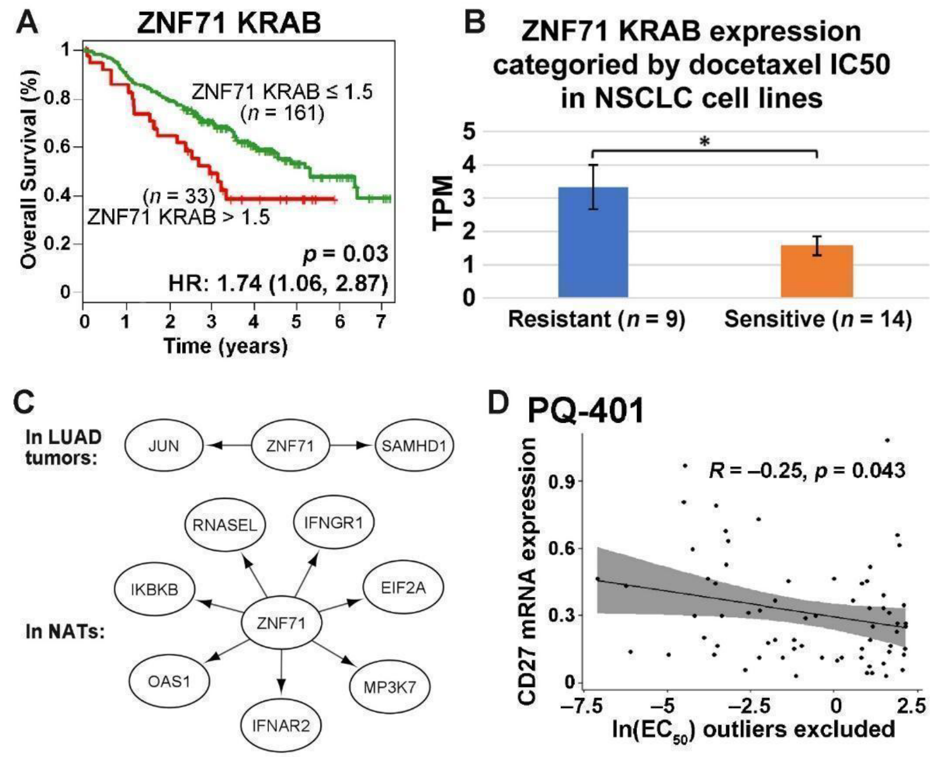

Acting as a bioinformatics professional. Please provide an overview on each panel.

<2-1-1> Certainly!

<2-1-2> Let's break down the information presented in each panel:.

<2-1-3> Panel A: Kaplan-Meier Survival Curve

<2-1-4> This graph represents the overall survival rates of patients over a period of time based on the expression of the ZNF71 KRAB gene.

<2-1-5> Two groups are presented: Patients with ZNF71 KRAB expression ≤ 1.5 (depicted in red) with a total of 161 patients. {KRAB ≤ 1.5 green.}

<2-1-6> Patients with ZNF71 KRAB expression > 1.5 (depicted in green) with a total of 33 patients. {KRAB > 1.5 red.}

<2-1-7> The Hazard Ratio (HR) is 1.74 with a 95% confidence interval of 1.06 to 2.87, which suggests that there might be a statistically significant difference in survival between the two groups.

<2-1-8> The p-value is 0.03, which indicates that the difference in survival between the two groups is statistically significant at a 0.05 significance level.

<2-1-9> Panel B: Bar Chart of ZNF71 KRAB Expression by Docetaxel Sensitivity

<2-1-10> This graph shows the ZNF71 KRAB gene expression (in TPM Transcripts Per Million) in NSCLC (Non-Small Cell Lung Cancer) cell lines categorized by their sensitivity to the drug docetaxel.

<2-1-11> Two groups are presented: Resistant cell lines (blue bar) with a total of 9 samples.

<2-1-12> Sensitive cell lines (orange bar) with a total of 14 samples.

<2-1-13> There's a statistically significant difference (indicated by the asterisk) between the two groups, suggesting that ZNF71 KRAB expression might be related to docetaxel sensitivity.

<2-1-14> Panel C: Gene Interaction Diagram

<2-1-15> This depicts the interactions of the ZNF71 gene with other genes in LUAD (Lung Adenocarcinoma) tumors and in NATs (presumably Normal Adjacent Tissues).

<2-1-16> In LUAD tumors, ZNF71 interacts with genes like JUN, SAMHD1, etc. {Only JUNB and SAMHD1 are shown interacting with ZNF71. There is no base for “etc”.}
<2-1-17> In NATs, ZNF71 interacts with different genes such as RNASEL, IFNGR1, and others.
*<2-1-18> Panel D: Scatter Plot of PQ-401 vs. CD271 mRNA Expression {CD27, not CD271. The identification on plot type (“scatter plot”) is correct.}
*<2-1-19> The graph represents the relationship between the natural logarithm (ln) of the effective concentration at 50% (EC50) for PQ-401 (excluding outliers) and the mRNA expression levels of CD271. {CD27, not CD271. The interpretation on “scatter plot” is correct.}
<2-1-20> The correlation coefficient, R, is -0.25, which suggests a weak negative correlation between the two variables.
<2-1-21> The shaded region indicates the confidence interval for the regression line.
<2-1-22> The p-value of 0.043 indicates that the correlation is statistically significant at the 0.05 level.
*<2-1-23> In summary, these panels seem to provide evidence on the significance of ZNF71 KRAB in relation to patient survival, drug sensitivity in NSCLC cell lines, gene interactions in different tissue types, and its potential relation with CD271 expression in the context of PQ-401. {CD27 not CD271. Other than the mis-spelling, the statement is correct.}

In Panel A: Is ZNF71 KRAB favorable or unfavorable? Why?

<2-1-24> In Panel A, the Kaplan-Meier Survival Curve compares the overall survival rates of patients based on the expression of the ZNF71 KRAB gene.
<2-1-25> From the graph: The red curve represents patients with ZNF71 KRAB expression ≤ 1.5. {KRAB ≤ 1.5 green.}
<2-1-26> The green curve represents patients with ZNF71 KRAB expression > 1.5. {KRAB > 1.5 red.}
<2-1-27> By examining the survival curves: Patients with a ZNF71 KRAB expression ≤ 1.5 (red curve) have a better survival rate over time than those with ZNF71 KRAB expression > 1.5 (green curve). {KRAB ≤ 1.5 green, KRAB > 1.5 red.}
<2-1-28> Given this information, ZNF71 KRAB expression at levels greater than 1.5 appears to be unfavorable for patient survival.
<2-1-29> This is because patients with higher expression of this gene have a lower survival rate over time compared to those with lower expression.

In Panel B: Is ZNF71 KRAB resistant or sensitive to docetaxel? Why and be quantitative?

<2-1-30> In Panel B, the bar chart displays the expression levels of ZNF71 KRAB in NSCLC (Non-Small Cell Lung Cancer) cell lines based on their sensitivity to docetaxel.
<2-1-31> From the graph: The blue bar represents cell lines that are resistant to docetaxel.
<2-1-32> The orange bar represents cell lines that are sensitive to docetaxel.
<2-1-33> Quantitatively: Resistant cell lines (blue bar) show a higher ZNF71 KRAB expression, roughly around 4 TPM (Transcripts Per Million). {The average expression in TPM for the resistant group is around 3.}
<2-1-34> Sensitive cell lines (orange bar) have a lower ZNF71 KRAB expression, approximately near 2 TPM. {The average expression in TPM for the sensitive group is around 1.5.}
<2-1-35> Given this quantitative data, cell lines with higher expression levels of ZNF71 KRAB (around 4 TPM) are resistant to docetaxel, while those with lower expression levels (around 2 TPM) are more sensitive to the drug. {The

average expression in TPM for the resistant group is around 3. The average expression in TPM for the sensitive group is around 1.5.}
<2-1-36> Thus, ZNF71 KRAB can be considered as associated with resistance to docetaxel in these NSCLC cell lines.

Q

In Panel C: Which genes are associated with ZNF71 in LUAD tumors? Which genes are associated with ZNF71 in non-cancerous adjacent tissues?

<2-1-37> In Panel C, the gene interaction diagram illustrates the associations of the ZNF71 gene with other genes in different tissue types.
<2-1-38> For LUAD (Lung Adenocarcinoma) tumors: ZNF71 is associated with the following genes: JUN SAMHD1.
*<2-1-39> For NATs (presumably Normal Adjacent Tissues): ZNF71 is associated with the following genes: RNASEL IFNGR1 IKKBK EIF2A OAS1 MP3K7 IFNAR2. {IKBKB, not IKKBK. Other than the mis-spelling. The identification of connections (positional relatiohsip) of the genes with ZNF71 is correct.}
<2-1-40> These associations indicate that the ZNF71 gene might interact differently in tumorous vs. non-tumorous tissues, suggesting potential roles in tumor biology and possibly in normal cellular functions.

Q

In Panel D: What is the R value in Panel D? What is the indication on the expression of CD27 and PQ-401 resistance?

*<2-1-41> In Panel D, the scatter plot represents the relationship between the natural logarithm (ln) of the effective concentration at 50% (EC50) for PQ-401 and the mRNA expression levels of CD271. {CD27, not CD271. The interpretation on the plot type itself is correct.}
<2-1-42> The given R value in Panel D is: R = -0.25.
<2-1-43> This R value indicates a weak negative correlation between the two variables.
<2-1-44> In the context of this scatter plot:.
*<2-1-45> A negative R value means that as the expression of CD271 increases, the ln(EC50) for PQ-401 decreases (or vice versa). {CD27, not CD271. The interpretation on R itself is correct.}
<2-1-46> Since EC50 represents the concentration of the drug required to achieve 50% of its maximum effect, a lower ln(EC50) suggests increased sensitivity to the drug (i.e., less drug is needed to achieve the desired effect), while a higher ln(EC50) suggests resistance.
*<2-1-47> Given the negative correlation: Higher expression levels of CD271 are associated with increased sensitivity to PQ-401, whereas lower expression levels of CD271 might be linked to resistance to PQ-401. {CD27, not CD271. The connection of CD27 expression with sensitivity (domain knowledge) itself is correct.}
<2-1-48> However, it's essential to note that the correlation is weak, so while there is a trend, it might not be a very strong predictor on its own.
<2-1-49> Further studies would be beneficial to confirm this association.

The "ZNF71" case study (in-depth assessment 2)

Q

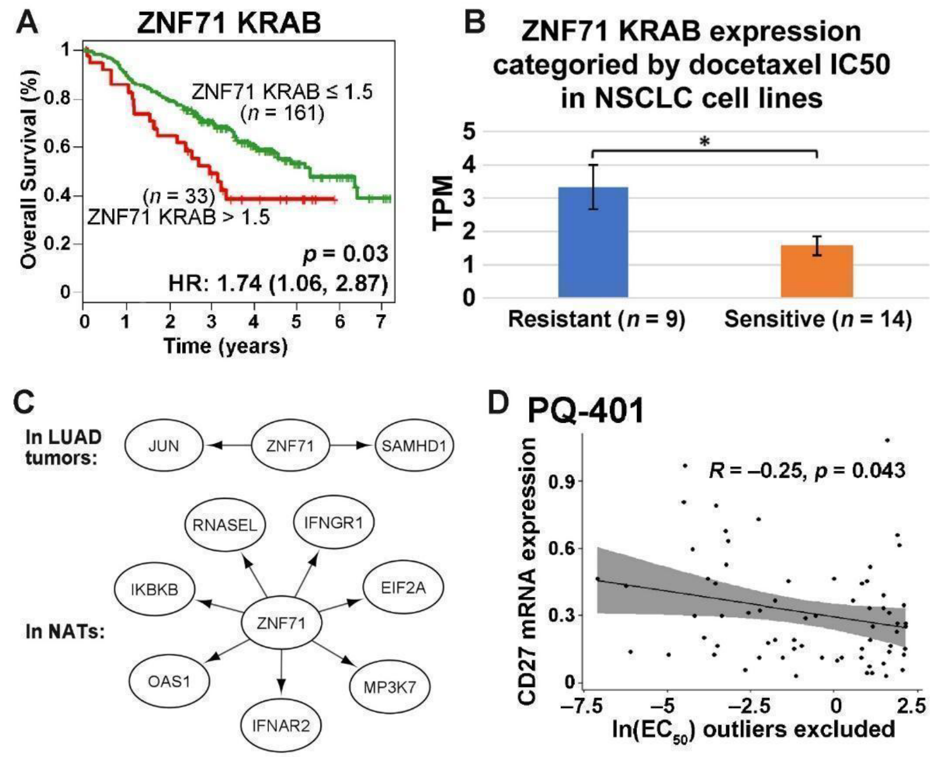

Acting as a bioinformatics professional. Please provide an overview on each panel.

<2-2-1> Certainly!

<2-2-2> Here's an overview of each panel in the image:.

<2-2-3> Panel A: Kaplan-Meier Survival Curve

<2-2-4> The Y-axis represents the overall survival rate of patients, and the X-axis represents the time in years.

<2-2-5> Two survival curves are shown:

<2-2-6> Red curve: Represents patients with ZNF71 KRAB expression ≤1.5. {KRAB ≤ 1.5 green.}

<2-2-7> The number of patients in this group is 161.

<2-2-8> Green curve: Represents patients with ZNF71 KRAB expression >1.5. {KRAB > 1.5 red.}

<2-2-9> The number of patients in this group is 33.

<2-2-10> The survival curve shows that patients with ZNF71 KRAB expression >1.5 have a lower survival rate over time.

<2-2-11> The hazard ratio (HR) for this comparison is 1.74 with a 95% confidence interval of 1.06 to 2.87.

<2-2-12> The p-value for this difference is 0.03, suggesting that the difference in survival between the two groups is statistically significant.

<2-2-13> Panel B: Bar Graph of ZNF71 KRAB Expression and Docetaxel Resistance The Y-axis represents ZNF71 KRAB expression in Transcripts Per Million (TPM).

<2-2-14> Two bars are shown:

<2-2-15> Blue bar: Represents NSCLC (Non-Small Cell Lung Cancer) cell lines that are resistant to docetaxel.

<2-2-16> There are 9 cell lines in this group.

<2-2-17> Orange bar: Represents NSCLC cell lines that are sensitive to docetaxel.

<2-2-18> There are 14 cell lines in this group.

<2-2-19> Cell lines that are resistant to docetaxel have higher ZNF71 KRAB expression compared to sensitive ones.

<2-2-20> The difference is marked by an asterisk, indicating statistical significance.

<2-2-21> Panel C: Pathway Diagram Displays the interactions and pathways involving the ZNF71 gene in Lung Adenocarcinoma (LUAD) tumors (top part) and in Normal Adjacent Tissues (NATs) (bottom part). {Partially incorrect: No pathway (domain knowledge) is indicated here. The use of domain knowledge for the full names of LUAD and NAT are correct.}
<2-2-22> In LUAD tumors, ZNF71 interacts with JUN and SAMHD1.
*<2-2-23> In NATs, ZNF71 has several interactions, including with RNASEL, IFNGR1, IKKB, EIF2A, OAS1, IFNAR2, and MP3K7. {IKBKB, not IKKB. Other than the mis-spelling, the identification of interaction (positional relationship) is correct.}
<2-2-24> The arrows indicate the directionality or influence of these interactions.
*<2-2-25> Panel D: Scatter Plot of PQ-401 and CD271 mRNA Expression {CD27, not CD271. The identification of plot type is correct though.}
*<2-2-26> The Y-axis represents CD271 mRNA expression, and the X-axis represents the natural logarithm of EC50 for PQ-401 (with outliers excluded). {CD27, not CD271. Other than that, the interpretation of the plot type is correct.}
<2-2-27> The scatter plot shows a correlation between the two variables, represented by the trendline.
*<2-2-28> The correlation coefficient (R) is -0.25, indicating a negative correlation between CD271 mRNA expression and the EC50 of PQ-401. {CD27, not CD271. The use of domain knowledge to interpret R itself is correct.}
<2-2-29> The p-value for this correlation is 0.043, suggesting that the correlation is statistically significant.
<2-2-30> In summary, these panels provide insights into the role and significance of ZNF71 KRAB expression in patient survival, its association with docetaxel resistance in NSCLC cell lines, its interaction pathways in both tumors and adjacent tissues, and its correlation with CD271 mRNA expression in the context of PQ-401 response. {Not pathway. CD27, not CD271. The correlation is not related to the ZNF71 KRAB expression.}

Q

In Panel A: Is ZNF71 KRAB favorable or unfavorable? Why?

<2-2-31> In Panel A, ZNF71 KRAB expression is depicted in relation to patient survival over time. .
<2-2-32> When we compare the two survival curves: Patients with ZNF71 KRAB expression ≤1.5 (Red curve) have a higher survival rate over the years than those with ZNF71 KRAB expression >1.5 (Green curve). {KRAB ≤ 1.5 green, KRAB > 1.5 red.}
<2-2-33> Given this observation, ZNF71 KRAB expression levels >1.5 are associated with a lower survival rate, making it unfavorable for patients.
<2-2-34> The reduced survival rate for this group suggests that higher expression of ZNF71 KRAB might be linked to a more aggressive disease or reduced response to treatment.
<2-2-35> Additionally, the hazard ratio (HR) of 1.74 for this group indicates an increased risk of death compared to the group with lower expression, reinforcing the unfavorable nature of higher ZNF71 KRAB expression.

Q

In Panel B: Is ZNF71 KRAB resistant or sensitive to docetaxel? Why and be quantitative?

<2-2-36> In Panel B, the bar graph depicts ZNF71 KRAB expression in NSCLC cell lines categorized by their sensitivity to docetaxel.

<2-2-37> From the graph: The blue bar represents cell lines that are resistant to docetaxel, and their average ZNF71 KRAB expression is higher, approximately around 4.5 TPM. {The average expression in TPM for the resistant group is around 3.}
<2-2-38> The orange bar represents cell lines that are sensitive to docetaxel, and their average ZNF71 KRAB expression is lower, approximately around 1.5 TPM.
<2-2-39> Given this quantitative data, cell lines with higher ZNF71 KRAB expression (around 4.5 TPM) are resistant to docetaxel, whereas cell lines with lower ZNF71 KRAB expression (around 1.5 TPM) are sensitive to docetaxel. {The average expression in TPM for the resistant group is around 3.}
<2-2-40> This indicates that higher levels of ZNF71 KRAB expression may confer resistance to the drug docetaxel.

Q

In Panel C: Which genes are associated with ZNF71 in LUAD tumors? Which genes are associated with ZNF71 in non-cancerous adjacent tissues?

<2-2-41> In Panel C, the interactions and pathways involving the ZNF71 gene in different tissue types are displayed. {Not pathway.}
<2-2-42> For LUAD tumors: The genes associated with ZNF71 are JUN and SAMHD1.
*<2-2-43> For Normal Adjacent Tissues (NATs): The genes associated with ZNF71 are RNASEL, IFNGR1, IKKB, EIF2A, OAS1, IFNAR2, and MP3K7. {IKBKB, not IKKB. The positional relationship of the genes and ZNF71 is correctly identified.}

Q

In Panel D: What is the R value in Panel D? What is the indication on the expression of CD27 and PQ-401 resistance?

<2-2-44> In Panel D: The correlation coefficient, R, is -0.25.
*<2-2-45> Given this value, there's a negative correlation between CD271 mRNA expression and the natural logarithm of EC50 for PQ-401. {CD27, not CD271. The interpretation of R as negative correlation itself is correct.}
*<2-2-46> This means that as CD271 mRNA expression increases, the EC50 for PQ-401 decreases (and vice versa). {CD27, not CD271. The interpretation of R as negative correlation itself is correct.}
<2-2-47> In terms of drug resistance, a lower EC50 value generally indicates that a lower concentration of the drug is required to achieve half-maximal inhibition, suggesting higher sensitivity to the drug.
<2-2-48> Conversely, a higher EC50 value suggests reduced drug sensitivity or increased resistance.
*<2-2-49> Given the negative correlation: Higher CD271 mRNA expression is associated with greater sensitivity to PQ-401 (or decreased resistance). {CD27, not CD271. The explanation on the negative correlation itself is correct.}
*<2-2-50> Lower CD271 mRNA expression is associated with reduced sensitivity or increased resistance to PQ-401. {CD27, not CD271. The explanation on the negative correlation itself is correct.}

The "ZNF71" case study (in-depth assessment 3)

Q

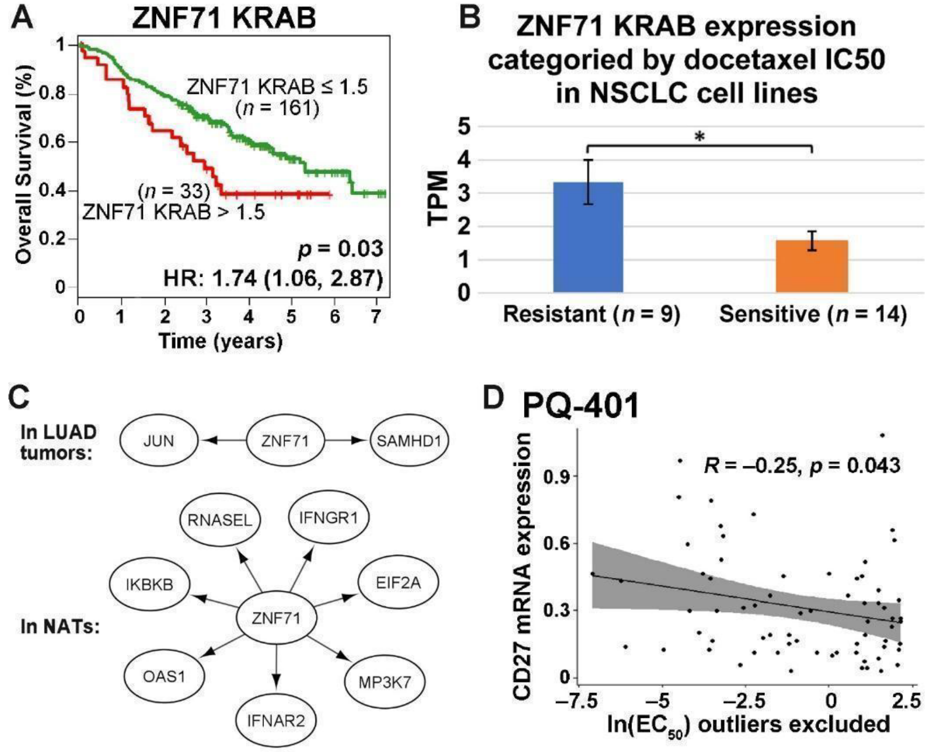

Acting as a bioinformatics professional. Please provide an overview on each panel.

<2-3-1> Certainly!

<2-3-2> Here's a breakdown of each panel:.

<2-3-3> Panel A: Survival Analysis

<2-3-4> This panel depicts the Kaplan-Meier survival curve for patients based on the expression levels of ZNF71 KRAB.

<2-3-5> Patients are divided into two groups: one with ZNF71 KRAB expression levels ≤1.5 (red line) and one with levels >1.5 (green line). {KRAB ≤ 1.5 green, KRAB > 1.5 red.}

<2-3-6> The x-axis represents time in years, and the y-axis represents the percentage of patients surviving.

<2-3-7> The red group seems to have a better overall survival than the green group. {Green is better}

<2-3-8> The hazard ratio (HR) is 1.74 with a 95% confidence interval of 1.06 to 2.87, suggesting a statistically significant difference in survival between the two groups.

<2-3-9> A p-value of 0.03 indicates this difference is statistically significant.

<2-3-10> Panel B: Expression Analysis in Relation to Drug Sensitivity This bar chart shows the expression levels of ZNF71 KRAB categorized by docetaxel IC50 (a measure of drug sensitivity) in NSCLC (Non-Small Cell Lung Cancer) cell lines.

<2-3-11> The y-axis represents expression levels in TPM (Transcripts Per Million).

<2-3-12> NSCLC cell lines are divided into two groups based on their sensitivity to docetaxel: Resistant (blue bar) and Sensitive (orange bar).

<2-3-13> Resistant cell lines show higher ZNF71 KRAB expression levels compared to sensitive ones.

<2-3-14> The asterisk denotes a significant difference between the two groups.

<2-3-15> Panel C: Gene Interaction Diagram

<2-3-16> This panel shows a gene interaction network centered around ZNF71.

<2-3-17> For LUAD tumors (Lung Adenocarcinoma): ZNF71 appears to influence JUN and SAMHD1.

*<2-3-18> For NATs (potentially normal adjacent tissues): ZNF71 seems to interact or influence multiple genes, including RNASEL, IFNGR1, IKKBK, EIF2A, OAS1, IFNAR2, and MP3K7. {IKBKB, not IKKB. The identification of positional relationship to ZNF71 is correct.}
<2-3-19> The interactions are denoted by arrows.
<2-3-20> Panel D: Correlation Plot
*<2-3-21> This scatter plot represents the relationship between the expression of CD271 mRNA and a drug named PQ-401 (probably an inhibitor) with its ln(EC50) values. {CD27, not CD271. The identification of the plot type is correct.}
<2-3-22> Outliers have been excluded for clarity.
*<2-3-23> The x-axis represents ln(EC50) values, a logarithmic measure of drug efficacy, and the y-axis represents CD271 mRNA expression levels. {CD27, not CD271. Other than the mis-spelling, the interpretation on plot type is correct.}
*<2-3-24> The trend line indicates a negative correlation (R = -0.25) between CD271 mRNA expression and ln(EC50), with a p-value of 0.043 suggesting this correlation is statistically significant. {CD27, not CD271. Other than the mis-spelling, others, including the interpretation on R, are correct.}
*<2-3-25> In summary, this figure provides information on the role of ZNF71 KRAB in patient survival, its correlation with drug sensitivity in NSCLC cell lines, its interaction with other genes, and the correlation between CD271 mRNA expression and drug efficacy. {CD27, not CD271.}

Q

In Panel A: Is ZNF71 KRAB favorable or unfavorable? Why?

<2-3-26> In Panel A, ZNF71 KRAB appears to be unfavorable for patient survival.
<2-3-27> The reason for this interpretation is:.
<2-3-28> The group of patients with higher expression levels of ZNF71 KRAB (>1.5) has a lower overall survival rate compared to those with lower expression levels (≤1.5).
<2-3-29> The green survival curve for the higher expression group declines more rapidly than the red curve of the lower expression group. {"Red" survival curve for the higher expression group declines more rapidly than the "green" curve of the lower expression group.}
<2-3-30> The hazard ratio (HR) provided is 1.74 (with a 95% confidence interval of 1.06 to 2.87).
<2-3-31> An HR greater than 1 suggests that there's a higher risk of the event occurring (in this case, death) in the group with higher expression levels compared to the group with lower expression levels.
<2-3-32> The combined observation of the survival curve and the hazard ratio suggests that higher levels of ZNF71 KRAB are associated with decreased overall survival, making it an unfavorable factor in the context presented.

Q

In Panel B: Is ZNF71 KRAB resistant or sensitive to docetaxel? Why and be quantitative?

<2-3-33> In Panel B, ZNF71 KRAB is associated with resistance to docetaxel.
<2-3-34> Here's why, based on the quantitative data provided:.
<2-3-35> The expression level of ZNF71 KRAB in the resistant group (blue bar) is higher than in the sensitive group (orange bar).
<2-3-36> Quantitatively, the average expression in TPM for the resistant group is around 4 (or slightly above), while for the sensitive group, it's around 1.5. {The average expression in TPM for the resistant group is around 3.}

<2-3-37> This suggests that cell lines with higher expression levels of ZNF71 KRAB are more resistant to docetaxel.
<2-3-38> The difference in expression levels, specifically around 2.5 TPM or more, indicates a stronger resistance to the drug in those cell lines with higher ZNF71 KRAB expression. {Difference was around 2.0.}

In Panel C: Which genes are associated with ZNF71 in LUAD tumors? Which genes are associated with ZNF71 in non-cancerous adjacent tissues?

<2-3-39> In Panel C:.
<2-3-40> For LUAD tumors (Lung Adenocarcinoma): ZNF71 is associated with the following genes: JUN SAMHD1.
*<2-3-41> For NATs (potentially normal adjacent tissues): ZNF71 is associated with the following genes: RNASEL IFNGR1 IKKBK EIF2A OAS1 IFNAR2 MP3K7. {IKBKB, not IKKBK. The full name of NAT (from domain knowledge) is correct.}
<2-3-42> These associations are depicted by the arrows, indicating interactions or influence between ZNF71 and the listed genes in the respective tissues.

In Panel D: What is the R value in Panel D? What is the indication on the expression of CD27 and PQ-401 resistance?

<2-3-43> In Panel D:.
<2-3-44> The R value, which represents the correlation coefficient, is R = -0.25.
*<2-3-45> The negative R value indicates a negative correlation between CD271 mRNA expression and ln(EC50) for PQ-401.{CD27, not CD271. The interpretation of R as negative correlation is correct.}
<2-3-46> This means that as the expression of CD271 mRNA increases, the ln(EC50) value decreases, suggesting an increased resistance to PQ-401. {CD27, not CD271. The decrease in ln(EC50) suggest increased sensitivity to PQ401.}
<2-3-47> Conversely, lower CD271 mRNA expression correlates with higher sensitivity to PQ-401. {CD27, not CD271. Lower CD271 mRNA expression correlates with higher ln(EC50) to PQ-401, higher ln(EC50) means higher resistance.}
<2-3-48> In simpler terms, higher expression of CD271 is associated with increased resistance to PQ-401, while lower expression of CD271 is associated with increased sensitivity to the drug. {Higher CD27 is associated with increased sensitivity. Lower CD27 is associated with increased resistance.}

Supplementary Notes S3

Supplementary Notes S3: Parsed, indexed, and annotated statements for the “Clonal” case study.

Instruction

Indexing principle: The indexing format adopted is <Case-Replicate-Statement>. In this format, “Case” represents the case study number, with 1 for “RNA”, 2 for “ZNF71”, 3 for “Clonal”, and 4 for “YY1”. The term “Replicate” indicates the replicate number, which can be either 1, 2, or 3. “Statement” corresponds to the statement’s sequential position within a specific replicate of a case study. For example, the third statement (3) in the second replicate (2) of the “RNA” case study (1) is indexed as <1-2-3>. Furthermore, a letter “B” is appended to the “Case” slot if the chat history originates from prompts associated with the basal assessment model.

Color-coding: True statements in **blue**, false ones in **red** (with errors highlighted in **yellow**), and chitchats (non-informative statements) in **black**, which are excluded from evaluation. An {explanation} highlighted in yellow in a curly bracket would append to the end of the false statement.

Overall performance

For the calculation of overall performance, a statement is marked as true if the contents for all its indicated categories are correct.

Replicate	Number of true statements	Number of evaluated statements	Accuracy
Clonal_rep1	75	109	68.81%
Clonal _rep2	66	101	65.35%
Clonal _rep3	76	102	74.51%

The “Clonal” case study (in-depth assessment 1)

Q

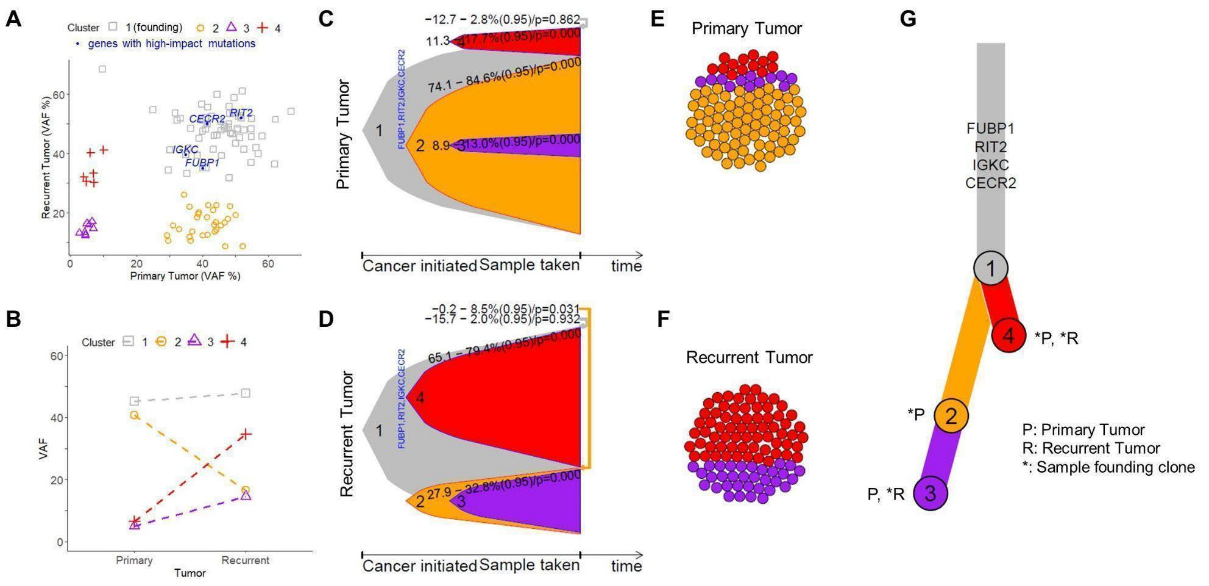

Acting as a bioinformatics professional proficient in cancer genomics data analysis, you will help interpret a figure to better appreciate clonal evolution in multiple myeloma (MM). The attached figure illustrates the results of analyzing somatic mutations detected in a pair of primary and recurrent tumor samples from a patient diagnosed with MM. Please do the following:

1. Provide an overview of each panel. Include the type of plot in the description, e.g., scatter plot, bar plot, etc.

<3-1-1> Certainly!
<3-1-2> Here's an interpretation of the figure's panels:.
<3-1-3> Panel A: Type of plot: Scatter plot
<3-1-4> Description: This plot compares the Variant Allele Frequency (VAF) of mutations in the primary tumor (x-axis) with their VAF in the recurrent tumor (y-axis).
<3-1-5> Each dot represents a mutation, color-coded by the clone they belong to.
<3-1-6> Notably, genes with high-impact mutations are highlighted with their respective gene names (e.g., CECR2, RIT2).
<3-1-7> The plot provides insight into how the prevalence of specific mutations changes between the primary and recurrent tumor stages.
<3-1-8> Panel B: Type of plot: Scatter plot with trend lines
<3-1-9> Description:
<3-1-10> This plot showcases the VAF of mutations in primary and recurrent tumors.
<3-1-11> Different mutation clusters are represented by distinct symbols and color codes.
<3-1-12> The trend lines illustrate the trajectory of VAF changes from the primary to recurrent stages for each mutation cluster.
<3-1-13> Panel C & D: Type of plot: Area plots Description: {Bell plot or simplified fish plot}
<3-1-14> These plots illustrate the clonal composition and their respective expansions or contractions in primary and recurrent tumor stages.
<3-1-15> The x-axis represents a hypothetical timeline from when the cancer was initiated to when the sample was taken.
<3-1-16> The y-axis could represent the proportion of each clone in the tumor.
<3-1-17> The p-values suggest significant changes in clonal compositions between the primary and recurrent tumors. {p-value testing for non-zero prevalence.}
<3-1-18> Panel E & F: Type of plot:

<3-1-19> Cell diagrams Description:
<3-1-20> These diagrams visually represent the clonal composition of the primary and recurrent tumors.
<3-1-21> Each circle represents a cell, and the color indicates its clonal affiliation.
<3-1-22> This gives a tangible sense of how dominant each clone is within the tumor at different stages.
<3-1-23> Panel G: Type of plot: Clonal tree diagram
<3-1-24> Description:
<3-1-25> This is a schematic representation of clonal evolution from the primary to the recurrent tumor.
<3-1-26> Clones are depicted as circles, with their size potentially representing their prevalence. {Sizes are all equal and do not present prevalence.}
<3-1-27> Arrows and branching indicate the emergence and evolution of new clones from their predecessors. {No arrows}
<3-1-28> Gene names listed are probably driver mutations or key mutations that characterize each clone.
<3-1-29> The annotations "P," "R," and "*" give information about which clones were present in the primary tumor, recurrent tumor, and the founding clone, respectively.
<3-1-30> Together, these panels offer a comprehensive view of how tumor composition evolves from the primary to the recurrent stage in this MM patient, highlighting the complexity of tumor heterogeneity and clonal dynamics.

Q

2. Panel A. Identify the number of clusters and their colors. Sort the clusters by size.

<3-1-31> In Panel A:. Clusters and Their Colors:.
<3-1-32> 1. Cluster 1 (founding): Color Purple 2. Cluster 2: Color Yellow 3. Cluster 3: Color Blue 4. Cluster 4: Color Red. {Not purple, should be Gray. Not blue, should be purple}
<3-1-33> Sorted by Size: By visually examining the scatter plot, we can roughly estimate the number of mutations in each cluster and sort them by size:.
<3-1-34> 1. Cluster 2 (Yellow): This cluster appears to have the most mutations, as seen by the dense collection of yellow dots. {It is the 2nd most mutation. The statement on color is correct.}
<3-1-35> 2. Cluster 3 (Blue): Following the yellow cluster, the blue cluster seems to have the second largest number of mutations. {Purple. It is the 3rd largest mutation.}
<3-1-36> 3. Cluster 4 (Red): This cluster has fewer mutations compared to the yellow and blue ones but more than the purple cluster. {Cluster 4 has the least number of mutations. The statement on color is correct.}
<3-1-37> 4. Cluster 1 (founding, Purple): This cluster has the least number of mutations, as indicated by the limited number of purple dots. {Gray. It has the most most number of mutations}
<3-1-38> This order provides an understanding of the prevalence of mutations across different clusters in the tumors.

Q

3. Panel B. Identify clusters showing an increase in VAF from primary to recurrent.

<3-1-39> In Panel B:.
<3-1-40> To identify clusters showing an increase in Variant Allele Frequency (VAF) from the primary to the recurrent tumor, we should focus on the trend lines (or trajectories) of each cluster.
<3-1-41> From the plot:.
<3-1-42> 1. Cluster 4 (Red trend line): This cluster displays a clear upward trajectory from the primary to the recurrent tumor.
<3-1-43> The mutations that belong to this cluster show higher VAF values in the recurrent tumor compared to the primary tumor.
<3-1-44> 2. Cluster 3 (Purple trend line): Though it starts with a high VAF in the primary tumor, there's a subtle increase as we move to the recurrent tumor. {It's low, not high}
<3-1-45> This indicates an increase in VAF for this cluster as well.
<3-1-46> Clusters 1 and 2, on the other hand, show either a decrease or relatively stable VAF from primary to recurrent.

<3-1-47> In conclusion, Clusters 3 and 4 show an increase in VAF from the primary to the recurrent tumor.

Q

4. Panels C and D. Identify the major cluster at the time of sample taken for each panel.

<3-1-48> For Panels C and D, the major cluster at the time of sample taken can be identified by observing the area plots at the "Sample taken" marker on the x-axis:. {Area covered on the y-axis determine the cluster size}
<3-1-49> Panel C (Primary Tumor): The largest section of the plot at the "Sample taken" marker is the orange color, which corresponds to Cluster 2.
<3-1-50> Therefore, Cluster 2 is the major cluster at the time the primary tumor sample was taken.
<3-1-51> Panel D (Recurrent Tumor): The dominant section of the plot at the "Sample taken" marker is the red color, which corresponds to Cluster 4.
<3-1-52> This means that Cluster 4 is the major cluster at the time the recurrent tumor sample was taken.
<3-1-53> In summary: For the primary tumor (Panel C), Cluster 2 is the major cluster.
<3-1-54> For the recurrent tumor (Panel D), Cluster 4 is the major cluster.

Q

5. Panels E and F. Sort the clusters by size for each panel.

<3-1-55> In Panels E and F, we can estimate the cluster sizes based on the number of circles (cells) representing each color.
<3-1-56> Panel E (Primary Tumor): 1. Yellow (Cluster 2): The dominant color with the most circles.
<3-1-57> 2. Red (Cluster 4): Fewer circles than yellow but more than the other two colors. {Only three groups here}
<3-1-58> 3. Blue (Cluster 3): Even fewer circles, making it the third most prevalent. {There is no blue on the plot.}
<3-1-59> 4. Purple (Cluster 1): The least number of circles in this panel, making it the smallest cluster. {Purple means cluster 3.}
<3-1-60> Panel F (Recurrent Tumor): 1. Red (Cluster 4): Now the dominant color in this panel with the most circles.
<3-1-61> 2. Purple (Cluster 1): Significantly increased from the primary tumor and is now the second most prevalent. {Purple means cluster 3.}
<3-1-62> 3. Blue (Cluster 3): Has fewer circles compared to red and purple, making it the third in size. {There is no blue on the plot.}
<3-1-63> 4. Yellow (Cluster 2): Drastically reduced in size from the primary tumor and is now the smallest cluster. {There is no yellow on the plot.}
<3-1-64> In summary: For the primary.
<3-1-65> tumor (Panel E): The order by size is Cluster 2, Cluster 4, Cluster 3, and then Cluster 1. {Cluster 2, 4, 3}
<3-1-66> For the recurrent tumor (Panel F): The order by size is Cluster 4, Cluster 1, Cluster 3, and then Cluster 2. {Cluster 4, 3}

Q

6. Panel G: Identify the cluster at the root and the clusters are the leaves.

<3-1-67> In Panel G, which represents the evolutionary tree of the clusters:.
<3-1-68> Root Cluster:
<3-1-69> The cluster at the base or origin of the tree is represented by the circle labeled 1, which is in red color {Cluster 1 is in gray.}
<3-1-70> This corresponds to Cluster 1.
<3-1-71> The root represents the original or founding clone.

<3-1-72> Leaf Clusters:
<3-1-73> The clusters that are at the tips or ends of the branches are considered the "leaves".
<3-1-74> In this panel, the leaf clusters are represented by circles labeled 2 (in purple color), 3 (in blue color), and 4 (in orange color). {Totally messed up the color-code for each cluster here: 2 should be in orange, 3 in purple, and 4 in red. Only cluster 3 and 4 are at the leaves}
<3-1-75> These correspond to Clusters 2, 3, and 4, respectively.
<3-1-76> In conclusion: Cluster 1 is at the root.
<3-1-77> Clusters 2, 3, and 4 are the leaves. {Cluster 2 is not he leaf}

7. Are there any specific suggestions to improve the data presentation of the figure?

<3-1-78> Certainly!
<3-1-79> While the figure provides a comprehensive representation of clonal evolution in multiple myeloma, there are areas where clarity and presentation can be improved.
<3-1-80> Here are some suggestions:.
<3-1-81> 1. Consistency in Color Representation:
<3-1-82> Throughout different panels, it's crucial to maintain consistent coloring for clusters.
<3-1-83> This reduces confusion.
<3-1-84> For example, in Panel G, Cluster 4 is represented in orange, whereas in other panels it's shown in red. {Cluster 4 is already in red.}
<3-1-85> 2. Enhance Legend Readability:
<3-1-86> A consolidated legend indicating each color with its corresponding cluster number and description could be helpful.
<3-1-87> This way, the reader doesn't need to jump between panels to decode colors.
<3-1-88> 3. Clear Labeling of Axes:
<3-1-89> In Panels A and B, ensuring that both axes are clearly labeled with their respective metrics can help.
<3-1-90> For instance, ensuring "VAF %" is clearly visible.
<3-1-91> 4. Simplified Statistical Annotations:
<3-1-92> The statistical notations in Panels C and D are comprehensive but can be overwhelming.
<3-1-93> Simplifying these or providing them in a supplementary note might make the main figure more digestible.
<3-1-94> 5. Clarity in Cluster Trajectories:
<3-1-95> In Panel B, using arrows to indicate the direction from primary to recurrent could provide a clearer understanding of VAF changes.
<3-1-96> 6. Zoom or Insets for Dense Areas: In Panels A and B, where data points are densely packed, a zoomed n inset could be beneficial for clarity. {B dose not have densely packed points.}
<3-1-97> 7. Panel G Improvements:
<3-1-98> Clarifying the meaning of '*P' and '*R' directly in the panel or in the legend can help. {Already have}
<3-1-99> Using line width to indicate the proportion of cells in each evolutionary path could provide a clearer visualization of dominance.
<3-1-100> 8. Improved Panel Titles:
<3-1-101> More descriptive panel titles or subtitles could provide a quick understanding of each panel's main takeaways.
<3-1-102> 9. Use of 3D or Interactive Plots:
<3-1-103> If this is for a digital publication, interactive plots where the user can hover over data points to get more information might enhance understanding.
<3-1-104> 10. Summary or Key Takeaways:
<3-1-105> A small textbox summarizing the key findings from the figure can aid those unfamiliar with the specifics of clonal evolution.
<3-1-106> Incorporating these suggestions can make the figure more accessible, especially to those who might be new to the topic or the specific visualization methods used.

8. Draft a title and figure legend. Make sure to include details.

<3-1-107> Title: "Clonal Evolution Dynamics in Multiple Myeloma: Analysis of Somatic Mutations in Primary and Recurrent Tumors".
<3-1-108> Figure Legend:.
<3-1-109> Panel A: Scatter plot representing the Variant Allele Frequency (VAF %) of somatic mutations in primary vs. recurrent tumors.
<3-1-110> Each point represents a mutation, colored based on its clonal cluster assignment:
<3-1-111> Cluster 1 (Founding): Purple triangles Cluster 2: Yellow circles Cluster 3: Blue squares Cluster 4: Red crosses. {Read gray as purple in cluster 1, and purple as blue in cluster 3.}
<3-1-112> Panel B: Line plot displaying the change in VAF from primary to recurrent tumors for each clonal cluster.
<3-1-113> Purple dashed line: Cluster 1 Yellow solid line: Cluster 2 Blue dash-dot line: Cluster 3 Red dotted line: Cluster 4. {Cluster 1 is in gray. Line for cluster 2 is dashed. Cluster 3 line is purple.}
<3-1-114> Panels C & D: Area plots illustrating the temporal clonal composition of the primary (C) and recurrent (D) tumors, from cancer initiation to the time the sample was taken. {Bell plots or fish plots}
<3-1-115> Shades represent the proportion of each cluster at different time points.
<3-1-116> Panels E & F: Hexagonal plots showcasing the clonal composition of the primary (E) and recurrent (F) tumors. {Identified circle as hexagons.}
<3-1-117> Each hexagon represents a cell, colored by its clonal assignment. {Identified circle as hexagons.}
<3-1-118> Panel G: Evolutionary tree diagram depicting the lineage relationship among the clusters.
<3-1-119> Circle size at the tree nodes corresponds to the cluster's size.
<3-1-120> The tree's root (Cluster 1) represents the founding clone, while the tips (Clusters 2, 3, and 4) denote evolved clusters. {Cluser 2 is not on the tip.}
<3-1-121> '*P' indicates the sample founding clone in the primary tumor.
<3-1-122> '*R' denotes the sample founding clone in the recurrent tumor.
<3-1-123> Note: VAF represents the proportion of sequencing reads supporting the mutant allele, providing insights into clonal prevalence.

9. Draft a paragraph to describe the findings from the figure. This is one paragraph for the result part of a manuscript. 150 words or less.

<3-1-124> In the presented analysis of somatic mutations from primary and recurrent multiple myeloma tumors, we observed distinct patterns of clonal evolution.
<3-1-125> Panel A's scatter plot reveals a divergence in the Variant Allele Frequency (VAF) between the tumor stages, with certain mutations becoming more prevalent in the recurrent tumor.
<3-1-126> This is corroborated in Panel B, which delineates an increased VAF for Clusters 3 and 4 during recurrence.
<3-1-127> Temporal clonal composition plots (Panels C & D) suggest that by the time of sampling, Cluster 4 dominated in the primary tumor, whereas Cluster 1 was prevalent in the recurrent tumor. {Cluster 2 dominates primary, while cluster 4 dominates recurrent.}
<3-1-128> The hexagonal plots (Panels E & F) visualize the clonal makeup, highlighting the prominence of Cluster 2 in the primary and Cluster 4 in the recurrent tumors. {Identified circle as hexagons.}
<3-1-129> Panel G's evolutionary tree underscores the foundational role of Cluster 1, with Clusters 2, 3, and 4 emerging as branches, elucidating the dynamic lineage relationships among the clusters in this MM patient.

The “Clonal” case study (in-depth assessment 2)

Q

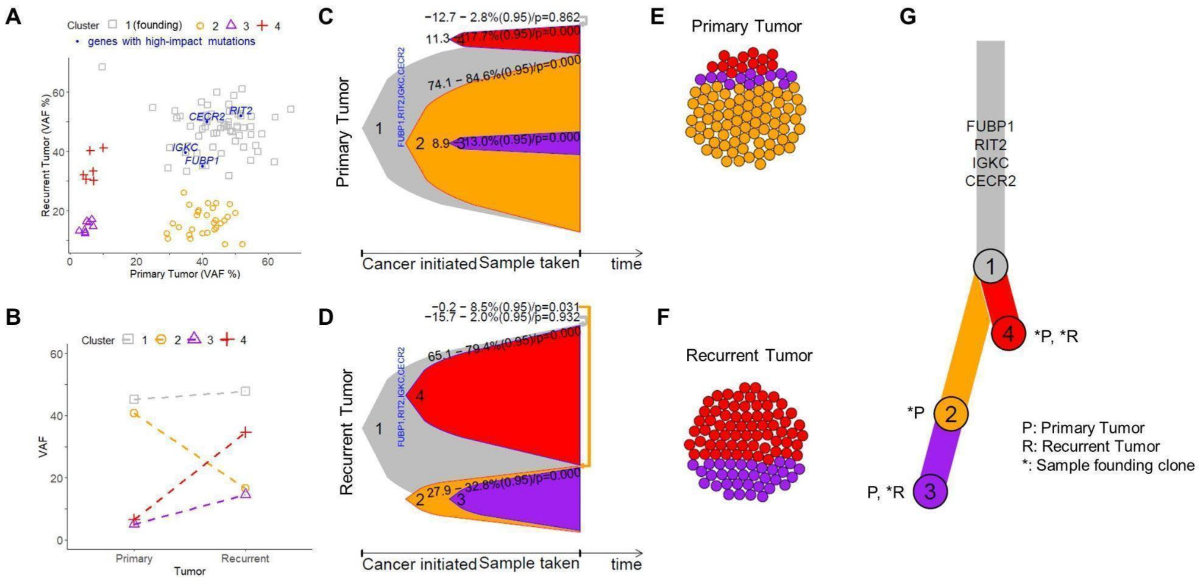

Acting as a bioinformatics professional proficient in cancer genomics data analysis, you will help interpret a figure to better appreciate clonal evolution in multiple myeloma (MM). The attached figure illustrates the results of analyzing somatic mutations detected in a pair of primary and recurrent tumor samples from a patient diagnosed with MM. Please do the following:

1. Provide an overview of each panel. Include the type of plot in the description, e.g., scatter plot, bar plot, etc.

<3-2-1> Certainly!
<3-2-2> Let's dive into the figure and interpret each panel.
<3-2-3> Panel A Type of Plot: Scatter plot Description:
<3-2-4> This panel displays the variant allele frequency (VAF) of mutations in the primary tumor (on the x-axis) versus the recurrent tumor (on the y-axis).
<3-2-5> Each point represents a unique mutation.
<3-2-6> The color and shape of the points represent different genetic clusters.
<3-2-7> The term "genes with high-impact mutations" indicates that the genes labeled on the plot (e.g., CECR2, RIT2) have mutations that likely have significant functional consequences.
<3-2-8> Panel B Type of Plot: Scatter plot with connecting lines
<3-2-9> Description:
<3-2-10> This panel represents the changes in VAF from the primary tumor to the recurrent tumor for specific genetic clusters.
<3-2-11> The x-axis shows the VAF in the primary tumor and the y-axis for the recurrent tumor. {X-axis means tumor status and y-axis means VAF.}
<3-2-12> The dotted lines connect the same genetic clusters between the primary and recurrent tumors, indicating the trajectory of mutation frequency changes.
<3-2-13> Panel C & D Type of Plot:
<3-2-14> Area plots Description: {Bell plot or fish plot}
<3-2-15> These panels illustrate the evolutionary trajectory of the tumor.
<3-2-16> The x xis represents time, starting from when the cancer initiated to when the sample was taken.
<3-2-17> The colored areas (orange, red, purple) depict the proportion of different genetic clusters over time. {Gray is missing}

<3-2-18> The values within the shaded areas indicate percentage change between the primary and recurrent tumors along with statistical significance (p-values). {p-value testing for non-zero prevalence}
<3-2-19> Panel E & F Type of Plot:
<3-2-20> Circle packing diagrams Description: These panels visualize the clonal composition of the primary tumor (E) and recurrent tumor (F).
<3-2-21> Each circle represents a group of cells with specific mutations. {Each circle represents of cells from each clone.}
<3-2-22> The color indicates different genetic clusters.
<3-2-23> The size of each circle correlates with the proportion of cells carrying those specific mutations in the tumor. {No size different here. Proportion of cells from the same clone.}
<3-2-24> Panel G Type of Plot: Schematic representation Description:
<3-2-25> This panel shows a tree-like structure representing the clonal evolution of the tumor.
<3-2-26> The trunk represents the founding clone, from which other sub-clones (branches) emerged.
<3-2-27> The numbers and colors on the tree correspond to the different genetic clusters, while the labels (e.g., FUBP1, RIT2) indicate genes with high-impact mutations.
<3-2-28> The annotations (*P, *R) suggest which clones were present in the primary and recurrent tumors, respectively.
<3-2-29> In summary, this figure provides comprehensive insights into the clonal evolution of a MM patient's tumor from diagnosis (primary) to recurrence.
<3-2-30> It highlights the genetic diversity, temporal dynamics, and relative abundance of different genetic clones over time.

Q

2. Panel A. Identify the number of clusters and their colors. Sort the clusters by size.

<3-2-31> From Panel A:. Number of Clusters and Their Colors:
<3-2-32> 1. Cluster 1 (founding): Red (`+` symbol) 2. Cluster 2: Yellow (circular symbol) {Cluster 1 is gray square.}
<3-2-33> 3
<3-2-34> .
<3-2-35> Cluster 3: Purple (triangular symbol) 4. Cluster 4: Light blue (square symbol). {Red Cross}
<3-2-36> Sorting Clusters by Size:
<3-2-37> To determine the size of the clusters, we typically look at the number of mutations (points) that belong to each cluster:.
<3-2-38> 1. Yellow Cluster (Cluster 2) This cluster seems to have the highest number of points, making it the largest cluster in terms of mutation count. {2nd highest}
<3-2-39> 2. Light blue Cluster (Cluster 4) This is the next largest cluster based on the number of points. {Cluster 4 is red and the smallest cluster.}
<3-2-40> 3. Red Cluster (Cluster 1) This is the founding cluster, and while foundational, it appears to have fewer points than Clusters 2 and 4. {Cluster 1 is in gray and the most in size.}
<3-2-41> 4.
<3-2-42> Purple Cluster (Cluster 3)
<3-2-43> This cluster seems to have the fewest points, making it the smallest in terms of mutation count.{3rd}
<3-2-44> So, sorted by size, the clusters are: Yellow (Cluster 2), Light blue (Cluster 4), Red (Cluster 1), and Purple (Cluster 3).{Gray(Cluster1), Red(Cluster4)}

Q

3. Panel B. Identify clusters showing an increase in VAF from primary to recurrent.

<3-2-45> In Panel B:.
<3-2-46> To determine which clusters show an increase in VAF from primary to recurrent tumors, we need to look at the positioning of the end points of the connecting lines with respect to the diagonal. {No need to compare to the diagonal.}

<3-2-47> If the end point for a cluster is above the diagonal, it indicates an increase in VAF from primary to recurrent. {No need to compare to the diagonal.}
<3-2-48> Based on this:.
<3-2-49> 1. Red Cluster (Cluster 1): The line for this cluster moves upward, indicating an increase in VAF from the primary to the recurrent tumor. {Red is for cluster 4.}
<3-2-50> 2. Purple Cluster (Cluster 3): The line for this cluster also moves upward, showing an increase in VAF from the primary to the recurrent tumor.
<3-2-51> Therefore, the clusters showing an increase in VAF from primary to recurrent tumors are the Red (Cluster 1) and Purple (Cluster 3) clusters.{Red is for cluster 4.}

Q

4. Panels C and D. Identify the major cluster at the time of sample taken for each panel.

<3-2-52> In Panels C and D, the major cluster at the time of sample taken can be identified by observing the cluster that occupies the largest area or proportion of the plot at the point labeled "Sample taken." {The largest width on the y-axis.}
<3-2-53> Panel C (Primary Tumor): The orange shaded region (corresponding to Cluster 4) is the predominant area at the time of sample taken, indicating that Cluster 4 was the major cluster in the primary tumor. {Orange is cluster 2. Should examine the width on y-axis other than the area.}
<3-2-54> Panel D (Recurrent Tumor): The red shaded region (corresponding to Cluster 1) is the predominant area at the time of sample taken, showing that Cluster 1 was the major cluster in the recurrent tumor.{Red is cluster 4. Should examine the width on y-axis other than the area.}
<3-2-55> In summary, for Panel C, the major cluster at the time of sample taken is Cluster 4, and for Panel D, it is Cluster 1.{Panel C is cluster 2. Panel D is cluster 4.}

Q

5. Panels E and F. Sort the clusters by size for each panel.

<3-2-56> Using Panels E and F, which employ circle packing diagrams, we can sort the clusters by size based on the total area occupied by each color (or cluster) within the panel.
<3-2-57> Panel E (Primary Tumor):.
<3-2-58> 1. Yellow (Cluster 2): This color dominates the panel with the most circles, indicating that Cluster 2 is the largest in the primary tumor.
<3-2-59> 2. Orange (Cluster 4): This is the second largest cluster based on its coverage. {Yellow/Orange is one cluster 2. Cluster 4 is red.}
<3-2-60> 3. Red (Cluster 1): While present, it occupies a smaller portion compared to the Yellow and Orange clusters.{Red is cluster 4. Red is the second largest.}
<3-2-61> 4. Purple (Cluster 3): This seems to be the smallest cluster in terms of size, with only a few circles in the diagram.
<3-2-62> Panel F (Recurrent Tumor):.
<3-2-63> 1. Red (Cluster 1): In this panel, the red circles dominate, indicating that Cluster 1 is the largest in the recurrent tumor.{Red is cluster 4.}
<3-2-64> 2. Purple (Cluster 3): This color is present with significant coverage, making it the second largest cluster.
<3-2-65> 3. Orange (Cluster 4): Although still present, this cluster occupies a smaller portion compared to Red and Purple clusters.{Orange is cluster 2. There is no orange circle present.}
<3-2-66> 4. Yellow (Cluster 2): This seems to be the smallest cluster in the recurrent tumor, with very few circles. {Orange is cluster 2. There is no yellow circle present.}
<3-2-67> In summary, for Panel E, the clusters sorted by size are: Yellow (Cluster 2), Orange (Cluster 4), Red (Cluster 1), and Purple (Cluster 3). {Orange is cluster 2. Red is cluster 4. Oder should be: Yellow/Orange > Red > Purple.}
<3-2-68> For Panel F, the clusters sorted by size are: Red (Cluster 1), Purple (Cluster 3), Orange (Cluster 4), and Yellow (Cluster 2). {Red is cluster 4. Orange is cluster 2. No orange or yellow. Order: Red > Purple>Orange (0) = Yellow (0).}

Q

6. Panel G: Identify the cluster at the root and the clusters are the leaves.

<3-2-69> In Panel G, which uses a tree or branch-like representation to illustrate clonal evolution:.
<3-2-70> Cluster at the Root:
<3-2-71> Red (Cluster 1): The base or starting point of the tree is depicted in red, indicating that Cluster 1 is the founding or root cluster. {Cluster 1 is gray.}
<3-2-72> Clusters at the Leaves:
<3-2-73> Orange (Cluster 4): This cluster branches out towards the top, making it one of the leaves {Toward the bottom. Cluster 4 is red.}.
<3-2-74> Purple (Cluster 3): Another branch that moves outwards, establishing it as a leaf.
<3-2-75> Yellow (Cluster 2): It also branches out from the main stem, indicating it's one of the leaf clusters. {Cluster 2 is not a leaf.}
<3-2-76> So, Cluster 1 is at the root, and Clusters 2, 3, and 4 are the leaves in Panel G. {Only cluster 4 (red) and cluster 3 (purple) are on the leaves.}

Q

7. Are there any specific suggestions to improve the data presentation of the figure?

<3-2-77> Certainly!
<3-2-78> Here are some suggestions to potentially improve the data presentation of the figure:.
<3-2-79> 1. Consistency in Color Coding:
<3-2-80> While the figure does seem to maintain consistent color coding for clusters across panels, ensuring this consistency can greatly aid in interpretation.
<3-2-81> 2. Legends and Labels:
<3-2-82> Having a consolidated legend that clearly labels each color to its respective cluster can help reduce confusion.
<3-2-83> Including clear axis labels and titles can also be beneficial, especially if the figure is viewed outside the context of its original publication.
<3-2-84> 3. Clarity in Panels C and D:
<3-2-85> The percentages and p alues in Panels C and D are a bit cluttered.
<3-2-86> Using a larger plot or reducing font size can make it easier to read.
<3-2-87> Additionally, clearer demarcations or lines at the "Sample taken" point can improve readability.
<3-2-88> 4. Use of 3D or Interactive Plots:
<3-2-89> If this is for a digital presentation or publication, consider using interactive plots, especially for Panels E and F.
<3-2-90> This would allow viewers to hover over specific clusters to get more information or to zoom in for a closer look.
<3-2-91> 5. Size Representation in Panels E and F:
<3-2-92> In the circle packing diagrams, the actual size of individual circles might be confused with the quantity.
<3-2-93> An accompanying note to clarify that the total area (and not individual circle size) represents cluster size could be beneficial.
<3-2-94> 6.
<3-2-95> Clarify Branch Thickness in Panel G:
<3-2-96> If the thickness of the branches in Panel G is indicative of anything (e.g., the number of mutations or the size of the cluster), this should be clearly mentioned.
<3-2-97> 7. Descriptive Captions:
<3-2-98> While not directly part of the figure, providing a detailed caption that briefly describes each panel can be immensely helpful to readers unfamiliar with the specifics of the data.
<3-2-99> 8. Enhance Visual Contrast: Ensure that there's enough contrast between different colors and the background, making it accessible to all readers, including those with visual impairments.

<3-2-100> 9. Annotations:
<3-2-101> Brief annotations directly on the plots (especially in Panels C, D, and G) can help highlight key observations or important data points.
<3-2-102> 10. Supplementary Materials:
<3-2-103> For figures with this level of complexity, it might be useful to have supplementary materials or an appendix that dives deeper into the methodology or offers additional plots for clarity.
<3-2-104> Incorporating some or all of these suggestions can make the figure more informative and user riendly, enhancing its overall impact and clarity.

Q

8. Draft a title and figure legend. Make sure to include details.

<3-2-105> Title: "Clonal Evolution of Somatic Mutations in Primary and Recurrent Multiple Myeloma".
<3-2-106> Figure Legend:.
<3-2-107> A. Scatter Plot of VAF in Primary vs. Recurrent Tumor:
<3-2-108> Each point represents a specific mutation, color-coded based on its respective cluster.
<3-2-109> The x-axis indicates the Variant Allele Frequency (VAF) in the primary tumor, while the y-axis represents the VAF in the recurrent tumor.
<3-2-110> B. Trajectory of Clusters from Primary to Recurrent Tumor:
<3-2-111> This plot tracks the shift in VAF of each mutation cluster from primary to recurrent phase.
<3-2-112> Each cluster is represented by its unique color and symbol.
<3-2-113> C & D. Temporal Dynamics of Clonal Evolution:
<3-2-114> These plots showcase the proportional representation of each mutation cluster over time, from cancer initiation to the point of sample collection.
<3-2-115> The 'Sample taken' mark indicates when the tumor samples were collected.
<3-2-116> C corresponds to the primary tumor and D to the recurrent tumor.
<3-2-117> P-values are provided for significant shifts in proportions. {p-value testing for non-zero prevalence}
<3-2-118> E & F.
<3-2-119> Circle Packing Diagrams of Clusters:
<3-2-120> These diagrams visually represent the relative size of each mutation cluster within the primary (E) and recurrent (F) tumors.
<3-2-121> The total area occupied by each color signifies the size of the respective cluster.
<3-2-122> G. Phylogenetic Tree of Clonal Evolution: Depicting the evolutionary relationship between mutation clusters.
<3-2-123> The root (red) represents the founding cluster, while the branches culminate in the leaf clusters, indicating subsequent clonal developments.{Root is in gray not red.}
<3-2-124> Note: In all panels, the colors red, yellow, orange, and purple correspond to clusters 1, 2, 4, and 3 respectively. {Color mismatch. Red = 4; Yellow/Orange = 2; Purple = 3; Gray = 1.}
<3-2-125> VAF denotes Variant Allele Frequency, an indicator of the proportion of mutated alleles to total alleles in the sample.

Q

9. Draft a paragraph to describe the findings from the figure. This is one paragraph for the result part of a manuscript. 150 words or less.

<3-2-126> In the study of clonal evolution in multiple myeloma, distinct mutation clusters were identified within primary and recurrent tumor samples from a single patient.
<3-2-127> As shown in Figure [X], a scatter plot analysis (Panel A) revealed the Variant Allele Frequency (VAF) dynamics between the primary and recurrent tumors, with some clusters demonstrating an increased VAF in the recurrent phase (Panel

B).
<3-2-128> The temporal dynamics (Panels C & D) highlighted the predominant mutation clusters at the time of sample collection.
<3-2-129> Circle packing diagrams further visualized the relative sizes of these clusters in primary (Panel E) and recurrent (Panel F) tumors.
<3-2-130> Most notably, a phylogenetic tree (Panel G) delineated the clonal evolutionary trajectory, pinpointing a founding cluster and its subsequent branching into newer clusters.
<3-2-131> Collectively, these findings illuminate the intricate clonal architecture and evolutionary progression of multiple myeloma, underscoring the dynamic nature of cancer genomics over time.

The “Clonal” case study (in-depth assessment 3)

Q

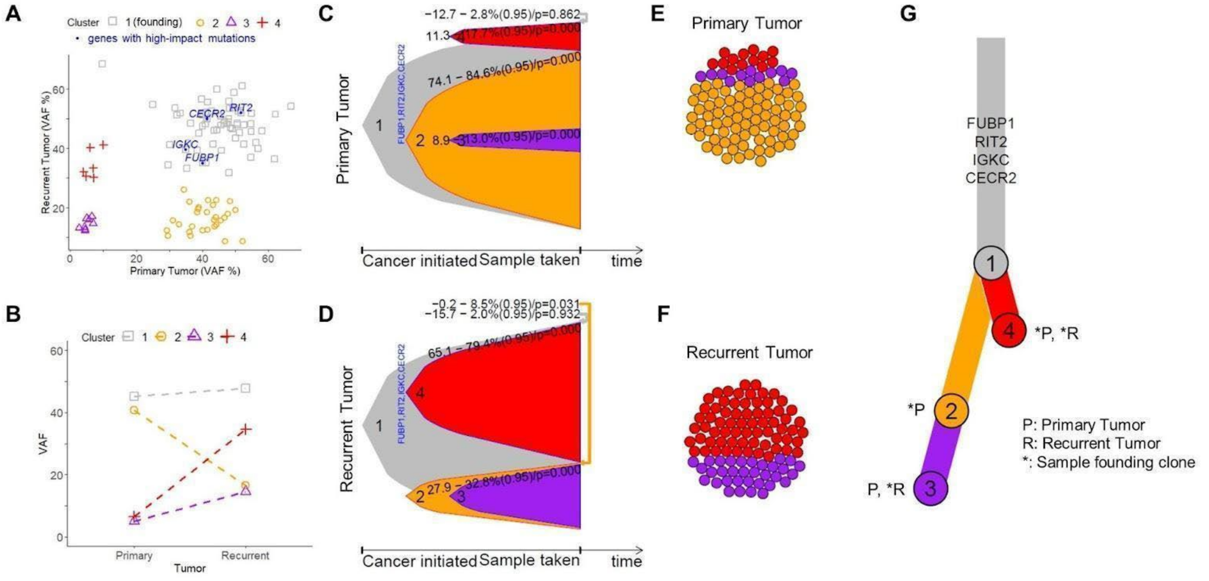

Acting as a bioinformatics professional proficient in cancer genomics data analysis, you will help interpret a figure to better appreciate clonal evolution in multiple myeloma (MM). The attached figure illustrates the results of analyzing somatic mutations detected in a pair of primary and recurrent tumor samples from a patient diagnosed with MM. Please do the following:

1. Provide an overview of each panel. Include the type of plot in the description, e.g., scatter plot, bar plot, etc.

<3-3-1> Certainly!
<3-3-2> Here's a breakdown of each panel in the figure:.
<3-3-3> Panel A Type of Plot: Scatter plot Overview:
<3-3-4> This panel presents the relationship between Variant Allele Frequencies (VAFs) of mutations detected in the primary and recurrent tumors.
<3-3-5> The x-axis represents the VAF percentages in the primary tumor, while the y-axis denotes the VAF percentages in the recurrent tumor.
<3-3-6> Different clusters of mutations are color-coded and labeled, with specific genes having high-impact mutations mentioned within the plot.
<3-3-7> Panel B Type of Plot: Scatter plot with connected lines Overview:
<3-3-8> This panel visually compares the VAF of mutations in the primary tumor to those in the recurrent tumor.
<3-3-9> Each mutation (or group of mutations) is connected with a dashed line from its primary tumor VAF (x-axis) to its recurrent tumor VAF (y-axis). {X-axis: tumor group. Y-axis: VAF}
<3-3-10> This kind of plot helps in observing changes in VAF between primary and recurrent tumor stages, indicating potential clonal expansion or reduction.
<3-3-11> Panel C Type of Plot: Stacked area plot Overview: {Bell plot or fish plot}
<3-3-12> This plot illustrates the trajectory of clonal evolution over time.
<3-3-13> The x-axis denotes the progression of the disease, from the initiation of the cancer to the point where the sample was taken.
<3-3-14> The y axis likely represents the percentage of tumor cells with specific mutations.
<3-3-15> The colored areas represent different mutation clusters and their prevalence over time.
<3-3-16> The numerical values and p-values might indicate statistical significance or shifts in the proportions of mutation clusters between primary and recurrent tumors. {p-value testing for non-zero prevalence}

<3-3-17> Panel D Type of Plot: Stacked area plot Overview: {Bell plot or fish plot}
<3-3-18> Similar to Panel C, this plot showcases the clonal evolution trajectory over time for the recurrent tumor.
<3-3-19> It demonstrates how the proportions of mutation clusters changed from the primary to the recurrent phase.
<3-3-20> Panel E Type of Plot: Circle packing diagram (or close packing of circles)
<3-3-21> Overview:
<3-3-22> This diagram represents the primary tumor's cellular composition concerning various mutation clusters.
<3-3-23> Each circle likely represents a group of cells, with different colors signifying different mutation clusters.
<3-3-24> Panel F Type of Plot:
<3-3-25> Circle packing diagram (or close packing of circles)
<3-3-26> Overview:
<3-3-27> Similar to Panel E, this diagram illustrates the cellular composition of the recurrent tumor concerning different mutation clusters.
<3-3-28> It shows the changes in cellular composition from the primary to the recurrent phase.
<3-3-29> Panel G Type of Plot: Clonal lineage tree Overview:
<3-3-30> This tree diagram displays the lineage of mutation clusters, starting with the founding clone and branching out to various derived clones.
<3-3-31> Labels provide specific genes associated with the mutation clusters.
<3-3-32> The "*P" and "*R" annotations denote clones present in the primary and recurrent tumors, respectively.
<3-3-33> Collectively, these panels provide a comprehensive view of how mutations in the tumor have evolved from the primary to the recurrent phase, offering insights into the clonal dynamics of this MM patient's disease.

Q

2. Panel A. Identify the number of clusters and their colors. Sort the clusters by size.

<3-3-34> Based on the figure in Panel A:. 1. Number of Clusters and Their Colors: Cluster 1 (founding): Purple triangle Cluster 2: Orange circles Cluster 3: Red plus signs (+) Cluster 4: Blue squares. 2. {Gray for cluster 1; Purple for cluster 3; Red for cluster 4.}
<3-3-35> Sorted by Size: Cluster 2 (Orange circles): This seems to be the largest cluster based on the number of data points. {Orange is the second most.}
<3-3-36> Cluster 3 (Red plus signs): The next in size, with a moderate number of data points. {Cluster is in purple. Red is for cluster 4. Both clusters are not the second most.}
<3-3-37> Cluster 4 (Blue squares): Fewer points compared to Cluster 2 and 3, but still more than the founding cluster. {Cluster 4 is in red. There is no clue cluster. Cluster 4 has the least.}
<3-3-38> Cluster 1 (founding) (Purple triangle): Contains the least number of points, indicating it's the smallest cluster. {Cluster 1 is in gray and is the most in size. Purple triangle is cluster 3, which is the third in size.}
<3-3-39> .
<3-3-40> Note: This sorting is based on the visible number of data points in Panel A.

Q

3. Panel B. Identify clusters showing an increase in VAF from primary to recurrent.

<3-3-41> In Panel B, clusters that show an increase in VAF from primary to recurrent would be depicted by points that move upwards from the primary (x-axis) to the recurrent (y-axis) tumor VAF. {X-axis: tumor group. Y-axis: VAF}
<3-3-42> From the plot:.
<3-3-43> 1. Cluster 3 (Red plus signs): The data points for this cluster seem to show a clear upward trajectory from the primary to the recurrent tumor, suggesting an increase in VAF. {Red is for cluster 4}
<3-3-44> 2. Cluster 4 (Blue squares): At least one of the data points for this cluster also seems to move upwards, indicating an increase in VAF for some mutations within this cluster. {Square on the plot is gray, and there is no blue color on the plot.}

<3-3-45> The other clusters either remain stable or decrease in VAF from primary to recurrent, based on the observed trajectories of their respective data points.

Q

4. Panels C and D. Identify the major cluster at the time of sample taken for each panel.

<3-3-46> In Panels C and D, the major cluster at the time of sample taken can be identified as the cluster that occupies the largest area or proportion at the far right end of the x xis (where the sample is taken). {The overlapping with y-axis by the area determine the cluster size.}
<3-3-47> Panel C (Primary Tumor): The major cluster appears to be the one represented by the orange area, which dominates the plot at the time of sample collection.
<3-3-48> Thus, the major cluster for the primary tumor is the one represented in orange.
<3-3-49> Panel D (Recurrent Tumor): The major cluster in this panel appears to be represented by the red area, which dominates the plot at the time of the recurrent sample collection.
<3-3-50> Hence, the major cluster for the recurrent tumor is the one represented in red.

Q

5. Panels E and F. Sort the clusters by size for each panel.

<3-3-51> Based on the circle packing diagrams in Panels E and F, we can sort the clusters by size by gauging the total area occupied by each color, representing different mutation clusters:.
<3-3-52> Panel E (Primary Tumor): 1.
<3-3-53> Orange Cluster: This cluster dominates most of the diagram and is the largest.
<3-3-54> 2. Red Cluster: The next largest, though significantly smaller than the orange cluster.
<3-3-55> 3.
<3-3-56> Purple Cluster: Has fewer circles than the red cluster, making it smaller in size.
<3-3-57> 4.
<3-3-58> Blue Cluster: Appears to be the smallest, with the least number of circles in the diagram.{There is no blue cluster.}
<3-3-59> Panel F (Recurrent Tumor): 1.
<3-3-60> Red Cluster: This cluster now takes up the most space in the diagram, making it the largest.
<3-3-61> 2. Purple Cluster: Significantly increased from Panel E and now is the second largest.
<3-3-62> 3.
<3-3-63> Blue Cluster: Still relatively small, but seems larger than in the primary tumor diagram. {There is no blue cluster show up.}
<3-3-64> 4. Orange Cluster: Appears to have shrunk considerably from Panel E and is now the smallest in Panel F. {There is no orange cluster show up.}
<3-3-65> This indicates dynamic shifts in the clonal populations from the primary to the recurrent tumor stages.

Q

6. Panel G: Identify the cluster at the root and the clusters are the leaves.

<3-3-66> In Panel G, the diagram seems to depict a phylogenetic or clonal evolution tree, where the root represents the founding or original clone, and the leaves represent the evolved or derived clones.
<3-3-67> Root:
<3-3-68> The cluster at the root is represented by the large circle labeled "1", which is colored in red. {Cluster 1 is in GRAY}

<3-3-69> This indicates the founding or primary clone from which all other clones derived.
<3-3-70> Leaves:
<3-3-71> The clusters that serve as leaves (or terminal nodes) in this evolutionary tree are: Circle labeled "2", which is colored in purple. Circle labeled "3", which is also colored in purple. {“2” is not a leave and it is in orange.}
<3-3-72> Circle labeled "4", which is colored in orange. {“4” is in red.}
<3-3-73> These leaf clusters represent the most evolved or derived clones from the founding clone, showcasing the clonal heterogeneity and evolutionary trajectory of the tumor.

Q

7. Are there any specific suggestions to improve the data presentation of the figure?

<3-3-74> Certainly!
<3-3-75> While the figure is quite informative, there are several suggestions that could potentially improve the data presentation and enhance its clarity:.
<3-3-76> 1. Consistent Color Coding:
<3-3-77> Ensure that the color code remains consistent across all panels.
<3-3-78> This helps the reader trace the progression of each cluster across different plots without confusion. .
<3-3-79> 2. Key/Legend:
<3-3-80> A unified legend for the entire figure would be beneficial.
<3-3-81> While some panels have annotations, a legend that details the color, shape, and any other pertinent symbols for each cluster would aid interpretation.
<3-3-82> 3. Scale and Axis Labels:
<3-3-83> Some panels lack clear axis labels or units.
<3-3-84> It's essential to label axes clearly in Panels A and B and provide scale where necessary. {Axes in Panels A and B already labeled clearly.}
<3-3-85> 4. Clarity in Panels C and D:
<3-3-86> The percentage figures and p alues in Panels C and D are a bit cluttered.
<3-3-87> Consider using a table or another format to present this numerical data more cleanly.
<3-3-88> 5. Enlarge Panels E and F:
<3-3-89> These panels are particularly informative but might benefit from being enlarged or given more space for clarity.
<3-3-90> This would help discern the smaller clusters more easily.
<3-3-91> 6. Simplify Panel G: While the tree diagram in Panel G is informative, it might benefit from some simplification or clearer labeling.
<3-3-92> For instance, the gray bar in the middle might be made more transparent or labeled to indicate its purpose.
<3-3-93> 7.
<3-3-94> Descriptive Titles for Each Panel:
<3-3-95> Brief descriptive titles above each panel would guide the reader on what to expect in each plot, making the figure more intuitive.
<3-3-96> 8. Annotate Significant Data Points:
<3-3-97> In scatter plots like Panel A and B, consider annotating or highlighting points that are outliers or are of particular significance.
<3-3-98> This can draw attention to key findings.
<3-3-99> 9.
<3-3-100> Panel Arrangement:
<3-3-101> Consider rearranging panels based on the flow of information or the story you want to convey, ensuring that related panels are adjacent or close to each other.
<3-3-102> 10. Quality of Text:
<3-3-103> Ensure that all text, especially in Panels C and D, is clear, readable, and of high resolution, even when zoomed in.
<3-3-104> Incorporating these suggestions could make the figure more reader friendly, enhancing the understanding and interpretation of the data presented.

Q

8. Draft a title and figure legend. Make sure to include details.

<3-3-105> Title: "Clonal Evolution and Mutation Dynamics in Primary and Recurrent Multiple Myeloma Tumors".
<3-3-106> Figure Legend: Figure X: Comprehensive analysis of clonal evolution in a patient with multiple myeloma, from primary to recurrent stages.
<3-3-107> Panel A: Scatter plot showing the Variant Allele Frequency (VAF) of mutations in the primary tumor (x xis) vs. the recurrent tumor (y xis).
<3-3-108> Each point represents a mutation, with colors indicating different mutation clusters: Red (Cluster 1 founding), Orange (Cluster 2), Purple (Cluster 3), and Blue (Cluster 4). {Colors for cluster 1 and cluster 2 are gray and red, respectively.}
<3-3-109> Panel B: Scatter plot illustrating the shift in VAF for each mutation cluster from the primary to the recurrent tumor.
<3-3-110> Symbols represent mutations within specific clusters: Red plus signs (Cluster 3), Orange circles (Cluster 2), Purple triangles (Cluster 1), and Blue squares (Cluster 4). {Cluster 3 = Purple; Cluster 1 = Gray; Cluster 4 = Red.}
<3-3-111> Panel C: Temporal clonal evolution diagram for the primary tumor.
<3-3-112> The width of each colored segment represents the proportion of each mutation cluster at a given time.
<3-3-113> P values indicate statistical significance of changes in cluster proportions. {p-value testing for non-zero prevalence.}
<3-3-114> Panel D: Temporal clonal evolution diagram for the recurrent tumor, with a similar representation as in Panel C.
<3-3-115> Panel E: Circle packing diagram representing the proportion and distribution of mutation clusters in the primary tumor.
<3-3-116> Each circle represents a mutation, with colors corresponding to specific clusters.
<3-3-117> Panel F: Circle packing diagram for the recurrent tumor, highlighting the shift in mutation clusters from the primary stage.
<3-3-118> Panel G: Clonal phylogenetic tree showcasing the evolutionary trajectory of mutation clusters.
<3-3-119> The root (red circle labeled "1") represents the founding clone, while terminal nodes (circles labeled "2", "3", and "4") represent derived clones. {"1" is in gray. "2" is not a leave node.}
<3-3-120> Note: Colors for mutation clusters are consistent across all panels.
<3-3-121> Red denotes Cluster 1, Orange represents Cluster 2, Purple is for Cluster 3, and Blue indicates Cluster 4. {On the plot: Gray for 1, Orange for 2, Purple for 3, Red for 4.}

Q

9. Draft a paragraph to describe the findings from the figure. This is one paragraph for the result part of a manuscript. 150 words or less.

<3-3-122> In our examination of clonal evolution in multiple myeloma, the paired analysis of primary and recurrent tumor samples revealed intricate dynamics of clonal shifts.
<3-3-123> Panel A's scatter plot uncovers a discernible correlation in the Variant Allele Frequency (VAF) between primary and recurrent tumors, with specific mutation clusters emerging prominently in the recurrent phase.
<3-3-124> This prominence is further visualized in Panel B, where Cluster 3 showcases an increased VAF from primary to recurrent stages. {Cluster 4 not cluster 3}
<3-3-125> Panels C and D intricately chart the temporal dominance of these mutation clusters, with Cluster 4 becoming a predominant presence by the time of the recurrent sample collection.
<3-3-126> The circle packing diagrams in Panels E and F further affirm this observation, spotlighting the clonal expansion in the recurrent tumor.
<3-3-127> The clonal phylogenetic tree in Panel G elegantly encapsulates the evolutionary trajectory, tracing back to a

foundational clone and branching out to diverse evolved clones, reflecting the complexity of clonal evolution in multiple myeloma.

Supplementary Notes S4

Supplementary Notes S4: Parsed, indexed, and annotated statements for the "YY1" case study.

Instruction

Indexing principle: The indexing format adopted is <Case-Replicate-Statement>. In this format, "Case" represents the case study number, with 1 for "RNA", 2 for "ZNF71", 3 for "Clonal", and 4 for "YY1". The term "Replicate" indicates the replicate number, which can be either 1, 2, or 3. "Statement" corresponds to the statement's sequential position within a specific replicate of a case study. For example, the third statement (3) in the second replicate (2) of the "RNA" case study (1) is indexed as <1-2-3>. Furthermore, a letter "B" is appended to the "Case" slot if the chat history originates from prompts associated with the basal assessment model.

Color-coding: True statements in **blue**, false ones in **red** (with errors highlighted in **yellow**), and chitchats (non-informative statements) in **black**, which are excluded from evaluation. An {explanation} highlighted in yellow in a curly bracket would append to the end of the false statement.

Overall performance

For the calculation of overall performance, a statement is marked as true if the contents for all its indicated categories are correct.

Replicate	Number of true statements	Number of evaluated statements	Accuracy
YY1_basal_rep1	74	76	97.37%
YY1_basal_rep2	78	80	97.5%
YY1_basal_rep3	74	77	96.1%
YY1_ indepth_rep1	122	167	73.05%
YY1_ indepth_rep2	122	163	74.85%
YY1_ indepth_rep3	137	163	84.05%

The “YY1” case study (basal assessment 1)

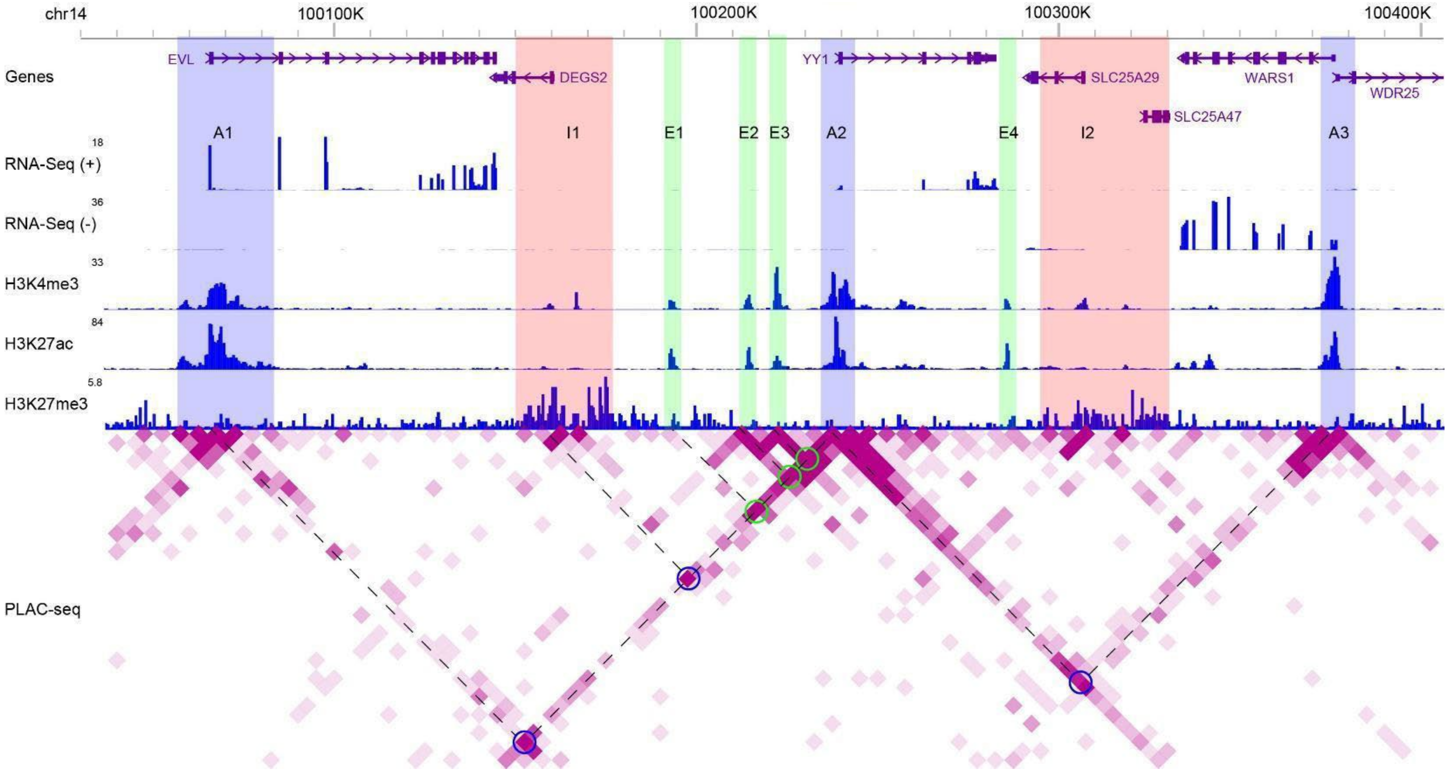

Acting as a bioinformatics professional proficient in RNA-Seq, ChIP-seq, and 3D chromatin analysis, you will help interpret a figure to appreciate transcription regulation at the chromatin level for YY1 in GM12878. The attached figure is a screenshot from the WashU genome browser, encompassing gene annotation, expression data, ChIP-Seq data, and chromatin-chromatin interaction data, for a genomic region enclosing YY1. The aim is to infer transcriptional regulatory mechanisms for YY1 from the epigenomic data. Please do the following.

1. Go over the tracks and explain specific findings addressing the aim.

<4B-1-1> Certainly, let's break down the figure to understand the transcriptional regulatory mechanisms for the gene YY1 in GM12878 based on the epigenomic data presented.

<4B-1-2> 1.

<4B-1-3> Gene Track (Genes):

<4B-1-4> The gene track shows multiple genes in this genomic region, with YY1 being the main gene of interest.

<4B-1-5> The arrows indicate the direction of transcription.

<4B-1-6> YY1 is transcribed from left to right, meaning it's on the plus strand.

<4B-1-7> 2. RNA-Seq Tracks (RNA-Seq (+) and RNA-Seq (-)): These tracks display the RNA sequencing data which reveals gene expression.

<4B-1-8> The plus and minus tracks show the expression levels for genes transcribed on the positive and negative strands respectively.

<4B-1-9> For YY1 (which is on the positive strand), we see distinct peaks, indicating that the YY1 gene is actively transcribed in GM12878.

<4B-1-10> 3.

<4B-1-11> ChIP-Seq Tracks: H3K4me3:

<4B-1-12> This is a marker for active promoter regions.

<4B-1-13> We observe a strong peak near the start of the YY1 gene, which suggests that this region functions as an active promoter for YY1 in GM12878.
<4B-1-14> H3K27ac:
<4B-1-15> This is a marker for active enhancer regions as well as active promoters.
<4B-1-16> We see multiple peaks across the region, indicating potential enhancer sites and further reinforcing the active promoter status of YY1.
<4B-1-17> H3K27me3:
<4B-1-18> This is a marker for repressed chromatin.
<4B-1-19> The lack of strong peaks in the vicinity of YY1 suggests that the chromatin is not in a repressed state around this gene.
<4B-1-20> 4.
<4B-1-21> PLAC-seq Track:
<4B-1-22> PLAC-seq is a method to capture long-range chromatin interactions.
<4B-1-23> The interaction heatmap reveals regions of the genome that are in close physical proximity, suggesting potential regulatory interactions.
<4B-1-24> Around the YY1 gene, we see some strong interactions (dark pink/red regions).
<4B-1-25> The circled areas indicate particularly significant interactions.
<4B-1-26> These interactions could be between the YY1 promoter and distant enhancer elements. {Not only interacting with distal enhancers but also promoters.}
<4B-1-27> By cross-referencing with the H3K27ac track, we can pinpoint potential enhancer regions that are in physical contact with the YY1 promoter, suggesting a direct regulatory relationship.
<4B-1-28> Interpretation: YY1 is actively transcribed in the GM12878 cell line, as indicated by the RNA-Seq data.
<4B-1-29> The YY1 promoter region is active, and there are several potential enhancer regions nearby, as shown by the H3K4me3 and H3K27ac peaks.
<4B-1-30> The YY1 promoter is interacting with distant regions, some of which may function as enhancers regulating YY1 expression.
<4B-1-31> These interactions can be mediated by protein complexes that bring these distant regions into close physical proximity to enhance YY1 transcription.
<4B-1-32> The lack of H3K27me3 suggests that the chromatin environment around YY1 is not repressive in GM12878.
<4B-1-33> Overall, the data suggests a complex regulatory landscape for YY1 in GM12878, with multiple potential enhancers in interaction with its promoter, ensuring its active transcription in this cell type.

Q

2. Are there any specific suggestions to improve the data presentation of the figure?

<4B-1-34> Certainly!
<4B-1-35> While the figure provides a wealth of information, there are a few suggestions that could improve its clarity and data presentation:.
<4B-1-36> 1. Color Contrast and Palette:
<4B-1-37> Ensure there's a good color contrast to make sure each track stands out distinctly.
<4B-1-38> This can help in quickly distinguishing between different data types.
<4B-1-39> Consider using a colorblind-friendly palette for all tracks to make the figure more accessible.
<4B-1-40> 2. Label Enhancements:
<4B-1-41> It might be helpful to label the significant peaks directly on the figure with short annotations.
<4B-1-42> For example, label the significant peaks in the H3K27ac and H3K4me3 tracks as potential 'enhancer' or 'promoter', respectively.

<4B-1-43> Enhance the gene labels, especially for YY1, to make it more pronounced than other genes.
<4B-1-44> 3. Zoomed-in Regions:
<4B-1-45> For regions with dense interactions or overlapping peaks, consider providing a zoomed-in panel to dissect the intricate details without crowding the main plot.
<4B-1-46> 4. Scale/Units:
<4B-1-47> Ensure that each track has a clear scale or unit of measurement (e.g., reads per million for RNA-Seq).
<4B-1-48> This helps in quantitatively comparing the data across different regions.
<4B-1-49> 5. Legends and Keys:
<4B-1-50> A clear legend explaining the color intensity in the PLAC-seq heatmap (indicating the strength of interaction) would be beneficial.
<4B-1-51> Add legends or a key for any symbols or special markings, like the circles in the PLAC-seq track.
<4B-1-52> 6. Interaction Highlight:
<4B-1-53> Consider using arrows or dashed lines to connect regions in the YY1 gene with the specific distant regions it interacts with on the PLAC-seq track.
<4B-1-54> This can visually guide the reader on which regions are interacting.
<4B-1-55> 7. Consolidate Tracks:
<4B-1-56> If certain tracks like RNA-Seq (+) and RNA-Seq (-) can be combined without loss of clarity, it might reduce the overall height of the figure, making it more compact.
<4B-1-57> Alternatively, collapsing tracks that aren't immediately necessary for the main point can also be considered.
<4B-1-58> 8.
<4B-1-59> Background Shading:
<4B-1-60> Use alternating light shades for different genomic regions or genes to provide a visual separation between them.
<4B-1-61> 9. Track Titles:
<4B-1-62> Make sure each track's title clearly defines what the track represents, possibly including the specific cell line or condition if applicable.
<4B-1-63> 10. Interactive Features (for online presentation):
<4B-1-64> If this figure is for an online platform, consider making it interactive.
<4B-1-65> For instance, hovering over a peak could provide additional details, or clicking on an interaction could highlight the corresponding regions on other tracks.
<4B-1-66> Implementing these suggestions can enhance the figure's comprehensibility, ensuring that the rich data it contains is easily accessible to readers, regardless of their familiarity with the specific field.

Q

3. Draft a title and figure legend. Make sure to include details.

<4B-1-67> Title: "Epigenomic Landscape and Chromatin Interactions Regulating YY1 Transcription in GM12878 Cells".
<4B-1-68> Figure Legend: Figure X: Multi-layered epigenomic data elucidating the transcriptional regulatory mechanisms of the YY1 gene in GM12878 cells. .
<4B-1-69> Genes:
<4B-1-70> Genomic annotations displaying the direction of transcription with arrows.
<4B-1-71> The highlighted gene is YY1. {YY1 is not the only gene highlighted in the plot}
<4B-1-72> RNA-Seq (+) and RNA-Seq (-): RNA sequencing tracks represent the expression levels of genes transcribed on the positive and negative strands, respectively.
<4B-1-73> Peaks in the RNA-Seq tracks denote active transcription.
<4B-1-74> H3K4me3:

<4B-1-75> ChIP-seq data showcasing the trimethylation of histone H3 at lysine 4.
<4B-1-76> Peaks represent active promoter regions.
<4B-1-77> .
<4B-1-78> H3K27ac: ChIP-seq data for histone H3 acetylation at lysine 27, a marker for active enhancer regions as well as active promoters.
<4B-1-79> H3K27me3:
<4B-1-80> ChIP-seq data indicating the trimethylation of histone H3 at lysine 27.
<4B-1-81> This mark is associated with repressed chromatin.
<4B-1-82> The absence of peaks near YY1 suggests non-repressive chromatin.
<4B-1-83> PLAC-seq: Chromatin interaction heatmap displaying regions of the genome in close physical proximity, indicating potential regulatory interactions.
<4B-1-84> Darker shades of pink/red signify stronger interactions.
<4B-1-85> Circled regions highlight significant chromatin interactions of interest.
<4B-1-86> Note: Data derived from the WashU genome browser, focusing on a genomic region encompassing the YY1 gene.
<4B-1-87> This comprehensive view provides insights into the intricate interplay of chromatin modifications and their potential role in regulating YY1 expression in GM12878 cells.
{Forget to explain shaded rectangles and dashed lines.}

Q

4. Draft a paragraph to describe the findings from the figure. This is one paragraph for the result part of a manuscript. 150 words or less.

<4B-1-88> In the epigenomic landscape surrounding the YY1 gene in GM12878 cells, our data reveals pronounced transcriptional activity as indicated by prominent RNA-Seq peaks.
<4B-1-89> Concomitantly, the presence of H3K4me3 at the YY1 start site designates an active promoter, while multiple H3K27ac peaks across the genomic stretch signify both an active promoter status and potential enhancer sites.
<4B-1-90> Notably, the absence of H3K27me3 suggests a non-repressed chromatin environment around the YY1 locus.
<4B-1-91> More intriguingly, PLAC-seq data delineates robust chromatin-chromatin interactions involving the YY1 promoter region.
<4B-1-92> These interactions, signified by darker hues on the heatmap, suggest a network of distal regulatory elements potentially orchestrating YY1 transcription.
<4B-1-93> Together, this multi-dimensional epigenomic overview underscores the intricate regulatory mechanisms governing YY1 expression in GM12878, with its promoter engaging in dynamic crosstalk with multiple distant regulatory elements.
{Missing details what regulatory regions could interacting with the YY1 promoter}

The “YY1” case study (basal assessment 2)

Q

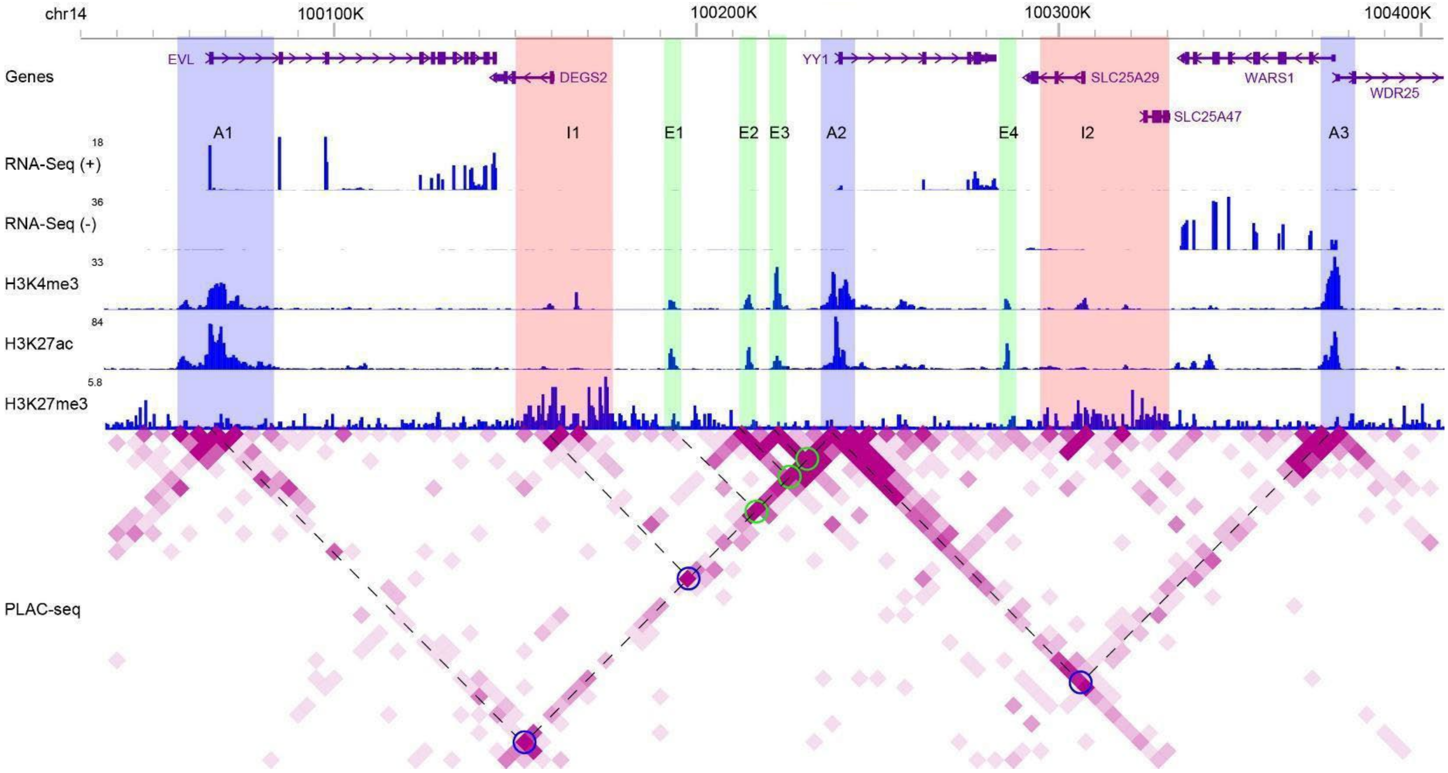

Acting as a bioinformatics professional proficient in RNA-Seq, ChIP-seq, and 3D chromatin analysis, you will help interpret a figure to appreciate transcription regulation at the chromatin level for YY1 in GM12878. The attached figure is a screenshot from the WashU genome browser, encompassing gene annotation, expression data, ChIP-Seq data, and chromatin-chromatin interaction data, for a genomic region enclosing YY1. The aim is to infer transcriptional regulatory mechanisms for YY1 from the epigenomic data. Please do the following.

1. Go over the tracks and explain specific findings addressing the aim.

<4B-2-1> Certainly!

<4B-2-2> Let's analyze the data step by step:.

<4B-2-3> 1. Gene Annotation (Genes):

<4B-2-4> At the top, the figure provides a gene annotation track, illustrating the genomic location and orientation of several genes, including `YY1`.

<4B-2-5> `YY1` is in the forward orientation, as indicated by the arrows.

<4B-2-6> 2. Expression Data (RNA-Seq):

<4B-2-7> Below the gene annotations are two RNA-Seq tracks representing RNA sequencing data on the positive (+) and negative (-) strands.

<4B-2-8> The height of the peaks in the RNA-Seq tracks represents the expression level of the genes.

<4B-2-9> Findings: `YY1` appears to be actively transcribed, as indicated by the peaks in the RNA-Seq (+) track directly above its location.

<4B-2-10> Other genes like `DEGS2`, `SLC25A29`, and `WARS1` also show active transcription. {DEGS2 and SLC25A29 are not expressed}

<4B-2-11> 3. ChIP-Seq Data for Histone Modifications:

<4B-2-12> The next three tracks show ChIP-Seq data for three histone modifications: H3K4me3, H3K27ac, and H3K27me3.
<4B-2-13> H3K4me3:
<4B-2-14> This modification is associated with active promoters.
<4B-2-15> Findings: There's a notable peak of H3K4me3 at the beginning of the `YY1` gene, suggesting the presence of an active promoter region there.
<4B-2-16> H3K27ac:
<4B-2-17> This modification indicates active enhancer regions or active promoters.
<4B-2-18> Findings:
<4B-2-19> We see multiple peaks of H3K27ac, especially around the `YY1` gene and between `YY1` and `DEGS2`, suggesting the presence of active enhancers or promoters in these regions.
<4B-2-20> H3K27me3:
<4B-2-21> This mark indicates repressed chromatin.
<4B-2-22> Findings:
<4B-2-23> There's no significant H3K27me3 peak in the vicinity of `YY1`, suggesting that the `YY1` region isn't repressed by this particular histone modification.
<4B-2-24> 4. Chromatin-Chromatin Interaction Data (PLAC-seq):
<4B-2-25> The bottom track illustrates chromatin-chromatin interactions detected by PLAC-seq.
<4B-2-26> Points on this track represent pairs of genomic loci that are in close physical proximity, potentially indicating regulatory relationships.
<4B-2-27> Findings:
<4B-2-28> The main diagonal line represents interactions between nearby genomic regions (due to the natural folding of DNA).
<4B-2-29> Off the diagonal, we see several highlighted points, indicating specific long-range interactions. {The highlighted points (circles) are on the diafonal.}
<4B-2-30> The circles highlight potential interactions of interest.
<4B-2-31> Notably, there are interactions between regions near the `YY1` promoter (with H3K4me3 and H3K27ac marks) and other regions with H3K27ac marks (indicative of active enhancers).
<4B-2-32> This suggests that the YY1 gene might be regulated by enhancers located at these interacting regions.
<4B-2-33> Inference:
<4B-2-34> From the given epigenomic data, we can infer the following about the transcriptional regulatory mechanisms of `YY1`:
<4B-2-35> The `YY1` gene is actively transcribed in GM12878 cells.
<4B-2-36> It possesses an active promoter region, as indicated by the H3K4me3 mark.
<4B-2-37> Several potential enhancer regions nearby (and possibly some at a distance) could be regulating its transcription, as evidenced by H3K27ac marks and chromatin-chromatin interactions.
<4B-2-38> There's no evidence of repression by the H3K27me3 mark in the vicinity of `YY1`.
<4B-2-39> In conclusion, `YY1` appears to be actively transcribed in GM12878, potentially regulated by multiple enhancer regions both nearby and at a distance, as indicated by the chromatin-chromatin interaction data.

Q

2. Are there any specific suggestions to improve the data presentation of the figure?

<4B-2-40> Certainly!
<4B-2-41> Effective visualization is critical for the interpretation and communication of complex genomic data.
<4B-2-42> Here are some suggestions to improve the data presentation of the figure:.

<4B-2-43> 1. Contrast & Color Selection:
<4B-2-44> While the current color scheme is functional, using a more diverse palette can help in distinguishing between different tracks more effectively.
<4B-2-45> However, ensure the palette is colorblind-friendly.
<4B-2-46> 2. Scale Consistency:
<4B-2-47> Make sure all tracks that measure similar things (like peak heights in RNA-Seq and ChIP-Seq data) have consistent scales, or at least clearly marked scales, so viewers can compare them directly.
<4B-2-48> 3. Enhance Interaction Points on PLAC-seq track:
<4B-2-49> The interaction points on the PLAC-seq track could be made more prominent or color-coded based on interaction strength.
<4B-2-50> This would allow viewers to quickly identify strong versus weak interactions.
<4B-2-51> 4. Labeling & Annotations:
<4B-2-52> Provide clear labels for each track.
<4B-2-53> This might seem obvious, but a concise description or title for each track can be incredibly helpful.
<4B-2-54> Offer a legend explaining the color-coding, especially if introducing a new palette.
<4B-2-55> Indicate the direction of gene transcription more clearly.
<4B-2-56> While the arrows are present, making them bolder or more prominent can be helpful.
<4B-2-57> 5.
<4B-2-58> Zoom-In Views:
<4B-2-59> For regions of particular interest, like the promoter of `YY1`, a zoomed-in view could be provided in a separate panel to showcase details.
<4B-2-60> 6. Additional Functional Annotation:
<4B-2-61> Overlay potential functional annotations, like transcription start sites (TSS), transcription factor binding sites, or DNase hypersensitivity sites, if available.
<4B-2-62> This would give more context to the ChIP-Seq peaks.
<4B-2-63> 7. Contextual Information:
<4B-2-64> A brief description or header indicating the cell type (GM12878) and the focus of the analysis (`YY1` transcriptional regulation) would set the context right away.
<4B-2-65> 8. Interactive Features (for online versions):
<4B-2-66> If this is being presented on a platform that supports interactivity, tooltips can be added.
<4B-2-67> Hovering over a region might provide additional details, like exact genomic coordinates or interaction strength values.
<4B-2-68> 9. Reducing Visual Noise:
<4B-2-69> Some tracks might have regions with minimal or no data.
<4B-2-70> Collapsing or minimizing these areas can help focus attention on the most relevant parts.
<4B-2-71> 10. Reference Lines:
<4B-2-72> Consider adding vertical reference lines across all tracks at key regions, like the `YY1` start site.
<4B-2-73> This can help in correlating features across tracks.
<4B-2-74> Implementing these suggestions can make the figure more accessible, interpretable, and valuable to both experts in the field and those less familiar with such data presentations.

Q

3. Draft a title and figure legend. Make sure to include details.

<4B-2-75> Title: Epigenomic Landscape Surrounding the `YY1` Gene in GM12878 Cells: Insights into Transcriptional Regulation Mechanisms.

<4B-2-76> Figure Legend:.
<4B-2-77> Overview:
<4B-2-78> This figure displays a comprehensive epigenomic overview of a genomic region encompassing the `YY1` gene in GM12878 cells.
<4B-2-79> The data is sourced from the WashU genome browser and includes gene annotations, expression profiles, histone modification patterns, and chromatin-chromatin interaction maps.
<4B-2-80> Tracks (from top to bottom):.
<4B-2-81> 1. Genes: Genomic annotation of genes in the region.
<4B-2-82> The direction of arrows indicates the transcriptional orientation of the genes.
<4B-2-83> Notable genes: `YY1`, `DEGS2`, `SLC25A29`, `WARS1`.
<4B-2-84> 2. RNA-Seq (+) and RNA-Seq (-): RNA sequencing data representing gene expression on the positive and negative strands.
<4B-2-85> Peak heights are indicative of transcriptional activity.
<4B-2-86> 3. H3K4me3: ChIP-Seq data for the H3K4me3 histone modification, a marker for active promoters.
<4B-2-87> 4. H3K27ac: ChIP-Seq data for the H3K27ac modification, associated with active enhancer regions and active promoters.
<4B-2-88> 5. H3K27me3: ChIP-Seq data for the H3K27me3 modification, indicating regions of repressed chromatin.
<4B-2-89> 6.
<4B-2-90> PLAC-seq: Chromatin-chromatin interaction map, revealing physical interactions between different genomic loci.
<4B-2-91> Highlighted points off the diagonal signify specific long-range interactions, with circles marking interactions of particular interest. {The circles are on the diagonal.}
<4B-2-92> Interpretation:
<4B-2-93> The presented data offers insights into the transcriptional regulation of the `YY1` gene.
<4B-2-94> Active transcription is observed for `YY1`, and its regulatory landscape includes potential enhancer regions and promoter activity as indicated by the H3K4me3 and H3K27ac marks.
<4B-2-95> The PLAC-seq interactions further suggest distant regulatory elements that may influence `YY1` expression.
{Forget to explain shaded rectangles and dashed lines.}

Q

4. Draft a paragraph to describe the findings from the figure. This is one paragraph for the result part of a manuscript. 150 words or less.

<4B-2-96> In our investigation of the transcriptional regulatory mechanisms governing the `YY1` gene in GM12878 cells, we observed distinct epigenomic signatures reflective of its active transcriptional state.
<4B-2-97> RNA-Seq data unequivocally confirmed the gene's expression.
<4B-2-98> A notable peak of the H3K4me3 histone mark, indicative of active promoters, was found at the onset of `YY1`.
<4B-2-99> Furthermore, surrounding regions exhibited prominent H3K27ac peaks, suggesting the presence of active enhancers or additional promoter activities.
<4B-2-100> Intriguingly, these H3K27ac-rich areas participated in notable chromatin-chromatin interactions, as evidenced by our PLAC-seq data.
<4B-2-101> Notably absent were significant H3K27me3 signals, negating the possibility of repression by this histone modification in the vicinity of `YY1`.

<4B-2-102> Cumulatively, our findings underscore the role of a dynamic epigenomic landscape, comprising active promoters, potential enhancers, and long-range chromatin interactions, in modulating the transcriptional activity of `YY1` in GM12878 cells.

{Missing details what regulatory regions could interacting with the YY1 promoter}

The "YY1" case study (basal assessment 3)

Q

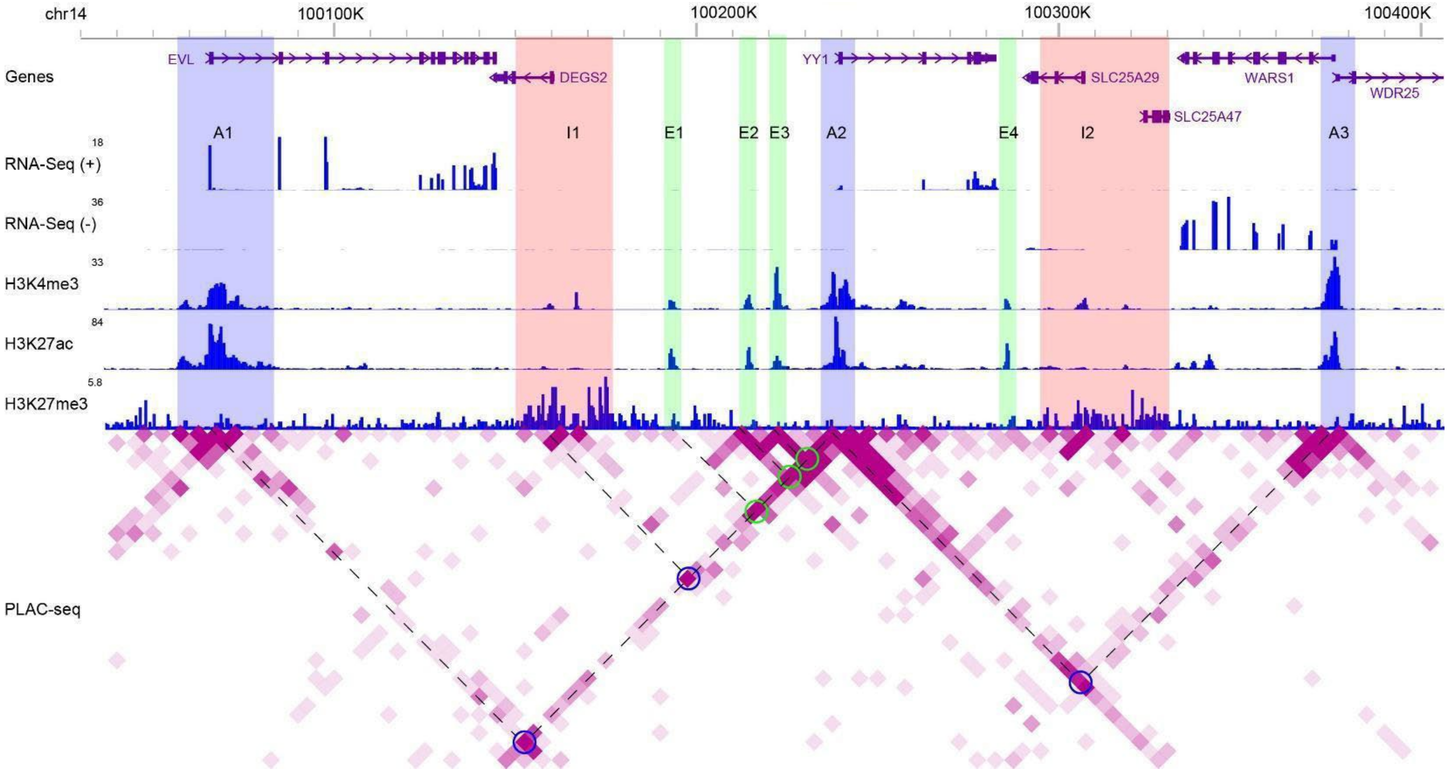

Acting as a bioinformatics professional proficient in RNA-Seq, ChIP-seq, and 3D chromatin analysis, you will help interpret a figure to appreciate transcription regulation at the chromatin level for YY1 in GM12878. The attached figure is a screenshot from the WashU genome browser, encompassing gene annotation, expression data, ChIP-Seq data, and chromatin-chromatin interaction data, for a genomic region enclosing YY1. The aim is to infer transcriptional regulatory mechanisms for YY1 from the epigenomic data. Please do the following.

1. Go over the tracks and explain specific findings addressing the aim.

<4B-3-1> Sure, let's break down the data tracks presented in this figure:.

<4B-3-2> 1. Gene Annotation Track:

<4B-3-3> The genes in the region are EVL, DEGS2, YY1, SLC25A29, WARS1, SLC25A47, and WDR25.

<4B-3-4> Our gene of interest, YY1, is in the middle of this genomic segment and it's transcribed from right to left (denoted by the arrow direction). {Left to right}

<4B-3-5> 2. RNA-Seq Tracks:

<4B-3-6> There are two RNA-Seq tracks: positive strand (+) and negative strand (-).

<4B-3-7> The peaks on these tracks represent the expression level of genes.

<4B-3-8> YY1 shows notable expression peaks in both strands, suggesting it is actively transcribed in GM12878 cells. {YY1 shows expression signals only in the positive strands.}

<4B-3-9> 3.

<4B-3-10> ChIP-Seq Data: H3K4me3 (Tri-methylation of lysine 4 on histone H3):

<4B-3-11> This mark is usually associated with active promoters.

<4B-3-12> A distinct peak is observed upstream of the YY1 transcription start site (TSS), suggesting an active promoter region.

<4B-3-13> H3K27ac (Acetylation of lysine 27 on histone H3):

<4B-3-14> This mark is found near active regulatory regions (both promoters and enhancers).
<4B-3-15> There are peaks observed in the YY1 region, supporting its active transcription and potential enhancer regions nearby.
<4B-3-16> H3K27me3 (Tri-methylation of lysine 27 on histone H3):
<4B-3-17> This mark is associated with transcriptionally repressed regions.
<4B-3-18> There seems to be little to no enrichment of this mark near the YY1 gene, indicating that there isn't significant transcriptional repression in this region for GM12878 cells.
<4B-3-19> 4. PLAC-seq (Chromatin-Chromatin Interaction Data):
<4B-3-20> The PLAC-seq track displays long-range interactions between different chromatin regions.
<4B-3-21> There are interactions (lines) connecting the YY1 region with other distant sites.
<4B-3-22> The highlighted circles signify the strength or frequency of these interactions. {Circles highlights interacting regions, not the strength or frequency of the interaction.}
<4B-3-23> The areas where YY1 interacts (highlighted in the PLAC-seq track) likely represent regulatory regions like enhancers that might be regulating YY1 transcription, as supported by the presence of the H3K27ac mark.
<4B-3-24> Summary: 1. YY1 is Actively Transcribed:
<4B-3-25> The RNA-Seq data shows distinct expression peaks for YY1, indicating active transcription in GM12878 cells.
<4B-3-26> 2. Active Promoter for YY1:
<4B-3-27> The H3K4me3 peak near the TSS of YY1 suggests the presence of an active promoter.
<4B-3-28> 3.
<4B-3-29> Potential Enhancer Regions:
<4B-3-30> The H3K27ac peaks around YY1, and the chromatin-chromatin interactions highlighted in the PLAC-seq data, suggest the presence of potential enhancer regions that might be regulating YY1 transcription.
<4B-3-31> 4. No Significant Transcriptional Repression:
<4B-3-32> The lack of a pronounced H3K27me3 peak suggests that the YY1 region isn't transcriptionally repressed in these cells.
<4B-3-33> In conclusion, the epigenomic data suggests that YY1 is actively transcribed in GM12878 cells, with evidence of both an active promoter and potential enhancer regions that may be involved in its regulation.
<4B-3-34> The chromatin-chromatin interaction data from PLAC-seq provides additional insight into the regulatory landscape, highlighting regions that may be interacting with the YY1 locus to modulate its expression.

Q

2. Are there any specific suggestions to improve the data presentation of the figure?

<4B-3-35> Certainly!
<4B-3-36> While the provided figure offers a comprehensive view of the genomic landscape surrounding the YY1 gene, there are always ways to improve data visualization for clarity and enhanced interpretation.
<4B-3-37> Here are some suggestions:.
<4B-3-38> 1. Consistent Color Scheme:
<4B-3-39> Using a consistent color scheme for related data can help in the quick identification of specific tracks.
<4B-3-40> For example, all RNA-Seq data could be in shades of blue, ChIP-Seq data in shades of purple, and interaction data in shades of red.
<4B-3-41> 2. Scale Adjustment:
<4B-3-42> Ensure all tracks are on a consistent and relevant scale.
<4B-3-43> If one particular ChIP-seq track has a significantly higher signal than others, consider normalizing or presenting on a log scale to avoid overshadowing other tracks.

<4B-3-44> 3. Track Labeling:
<4B-3-45> While the tracks are labeled, providing a brief description or legend for each track might be helpful for those less familiar with the data types.
<4B-3-46> For instance, briefly describing what H3K4me3, H3K27ac, and H3K27me3 represent can aid interpretation.
<4B-3-47> 4. Highlight Key Regions:
<4B-3-48> Important regions, like the promoter of YY1 or potential enhancers, can be highlighted or annotated with text labels directly on the figure.
<4B-3-49> 5. Interactive Features (if feasible):
<4B-3-50> If this visualization is meant for an online platform, interactive features like zooming, panning, and tooltips with more information about specific regions or peaks can be extremely helpful.
<4B-3-51> 6. Simplify Data where Possible:
<4B-3-52> For a general audience or a high-level presentation, consider simplifying the view by showing only the most relevant tracks.
<4B-3-53> For instance, if the main focus is YY1 transcription, perhaps show only the RNA-Seq data, the H3K4me3, H3K27ac, and the PLAC-seq interaction data.
<4B-3-54> 7. Grid Lines and Reference Points:
<4B-3-55> Adding faint grid lines or specific reference points can help in correlating peaks across different tracks.
<4B-3-56> 8. Improve Resolution of PLAC-seq Data:
<4B-3-57> The PLAC-seq data is dense, and it might be beneficial to increase the resolution or clarity of interaction points, especially those that are of particular interest.
<4B-3-58> 9. Order Tracks by Relevance:
<4B-3-59> Tracks can be ordered by their relevance to the central aim of the study.
<4B-3-60> For instance, if the primary aim is to explore YY1 transcription, then RNA-Seq data can be placed closest to the gene annotation track, followed by ChIP-seq data and then PLAC-seq data.
<4B-3-61> 10. Unified Peak Highlighting:
<4B-3-62> If certain peaks or regions in different tracks correspond to the same genomic location, consider using the same color or style to highlight them for easy cross-reference.
<4B-3-63> Implementing these suggestions can enhance the visual appeal of the figure, streamline data interpretation, and cater to both expert and general audiences.

Q

3. Draft a title and figure legend. Make sure to include details.

<4B-3-64> Certainly!. ---.
<4B-3-65> Title: Epigenomic Landscape and Chromatin Interactions Surrounding the YY1 Gene in GM12878 Cells.
<4B-3-66> ---.
<4B-3-67> Figure Legend:.
<4B-3-68> Figure X: Comprehensive epigenomic profiling of the YY1 gene region in GM12878 cells.
<4B-3-69> .
<4B-3-70> Gene Annotation:
<4B-3-71> This track displays the genomic context, including several genes with YY1 in the center.
<4B-3-72> The direction of arrows indicates the transcription direction.
<4B-3-73> RNA-Seq (+/-):
<4B-3-74> Expression levels from RNA sequencing data are shown for both positive (+) and negative (-) strands.
<4B-3-75> Peaks represent active transcription regions.
<4B-3-76> Notably, YY1 shows significant expression in these cells.

<4B-3-77> H3K4me3 ChIP-Seq:
<4B-3-78> Tri-methylation of lysine 4 on histone H3 is associated with active promoters.
<4B-3-79> A distinct peak upstream of YY1's transcription start site suggests an active promoter region.
<4B-3-80> H3K27ac ChIP-Seq:
<4B-3-81> Acetylation of lysine 27 on histone H3 indicates active regulatory regions.
<4B-3-82> Peaks around YY1 hint at active transcription and potential enhancer regions.
<4B-3-83> H3K27me3 ChIP-Seq:
<4B-3-84> Tri-methylation of lysine 27 on histone H3 marks transcriptionally repressed regions.
<4B-3-85> Minimal enrichment near YY1 indicates a lack of significant transcriptional repression.
<4B-3-86> PLAC-seq: Chromatin-chromatin interaction data unveils long-range interactions between chromatin regions.
<4B-3-87> Interactions connecting YY1 with distant sites could represent enhancer regions or other regulatory elements.
<4B-3-88> Colored rectangles (e.g., A1, E1, etc.) mark specific regions of interest for more detailed study or reference.
{Forget to mention circles, shaded rectangles, and dashed lines.}
<4B-3-89> ---.
<4B-3-90> When using this title and legend, please ensure that it fits the context and format of the publication or presentation, and adjust as needed.

Q

4. Draft a paragraph to describe the findings from the figure. This is one paragraph for the result part of a manuscript. 150 words or less.

<4B-3-91> Certainly!. ---.
<4B-3-92> In the GM12878 cellular context, the transcriptional regulatory landscape surrounding the YY1 gene was extensively characterized using multi-layered epigenomic data.
<4B-3-93> RNA-Seq analysis confirmed YY1's active transcription, as indicated by prominent expression peaks on both transcriptional strands.
<4B-3-94> The presence of an H3K4me3 peak proximal to the YY1 transcription start site underscores an active promoter, while H3K27ac enrichment suggests regions of transcriptional activation and potential enhancer activity nearby.
<4B-3-95> Notably, the scarcity of the H3K27me3 mark around YY1 indicates minimal transcriptional repression.
<4B-3-96> Importantly, PLAC-seq data revealed long-range chromatin interactions between YY1 and distant genomic regions, hinting at potential distal regulatory elements.
<4B-3-97> Collectively, these data highlight a dynamic chromatin environment around YY1 in GM12878 cells, pointing to multiple regulatory elements that likely govern its transcriptional activity. ---
{Missing details what regulatory regions could interacting with the YY1 promoter}

The “YY1” case study (in-depth assessment 1)

Q

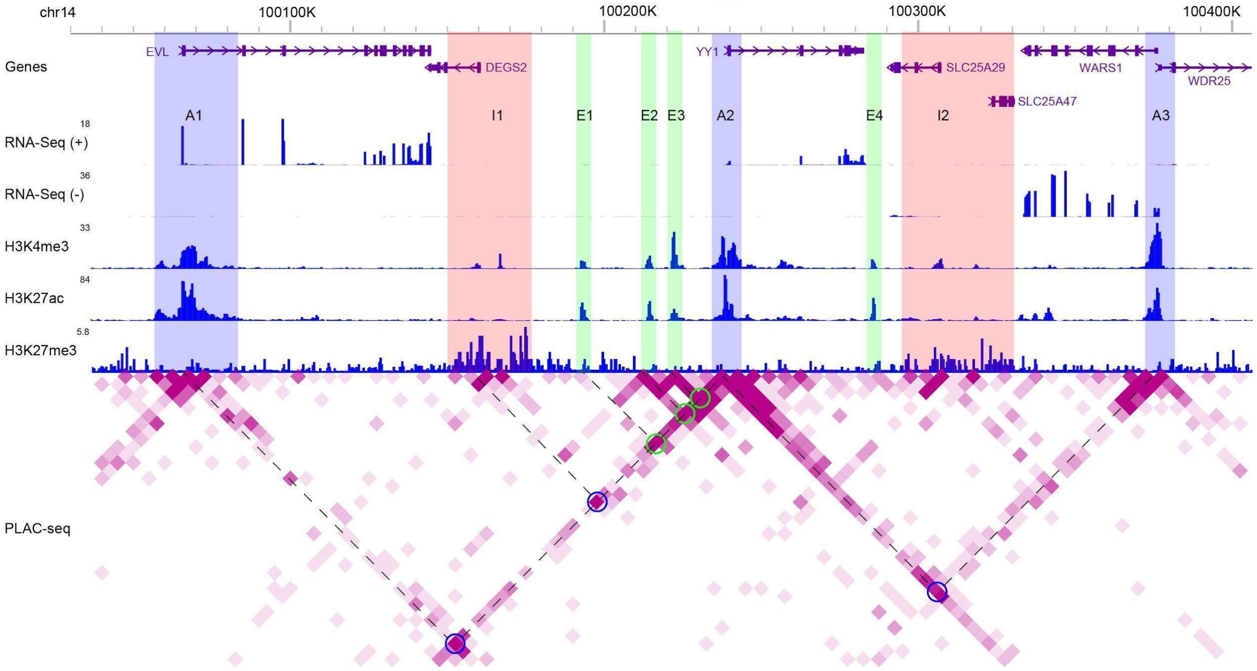

Acting as a bioinformatics professional proficient in RNA-Seq, ChIP-seq, and 3D chromatin analysis, you will help interpret a figure to appreciate transcription regulation at the chromatin level for YY1 in GM12878. The attached figure is a screenshot from the WashU genome browser, encompassing gene annotation, expression data, ChIP-Seq data, and chromatin-chromatin interaction data, for a genomic region enclosing YY1. The aim is to infer transcriptional regulatory mechanisms for YY1 from the epigenomic data. You will be guided with questions to identify findings addressing the aim. Please do the following.

1. Go over the RefSeq gene track and spell out the genes shown on the plot. Explain the presentation of gene structure. For example, what does the arrowhead mean?

<4-1-1> Sure, let's break down the figure step-by-step.

<4-1-2> 1. RefSeq gene track and gene structure: Genes shown on the plot:

<4-1-3> The genes presented on this plot, moving from left to right, are EVL, DEGS2, YY1, SLC25A29, WARS1, SLC25A47, and WDR25. {SLC25A47 comes before WARS1.}

<4-1-4> Presentation of gene structure:

<4-1-5> The genes are represented as horizontal lines that span their genomic regions.

<4-1-6> The directionality of each gene is indicated by the arrowheads.

<4-1-7> These arrows help to visually understand the direction in which the gene is transcribed, with the direction of the arrow indicating the 5' to 3' direction of the gene.

<4-1-8> Exons are depicted as thicker blocks, while introns are shown as thin lines connecting these blocks.

<4-1-9> This visual representation helps in distinguishing the coding regions (exons) from the non-coding regions (introns) of the gene.

<4-1-10> The arrowheads, or the direction of the arrows, help in understanding whether the gene is on the "+" (plus) or "-" (minus) strand.

<4-1-11> For example, YY1 has arrowheads pointing to the right, which means it is transcribed from left to right on the "+" strand.

<4-1-12> Conversely, genes like DEGS2, with arrowheads pointing to the left, are transcribed from right to left and are on the "-" strand.

<4-1-13> With this information, we can infer the orientation and structure of each gene in the genomic region shown.

<4-1-14> Would you like to proceed with the next questions or further explore this aspect?

Q

2. Check the expression data. What do “(+)” and “(-)” mean? Which genes are expressed and why?

<4-1-15> 2. Expression Data:.
<4-1-16> What do “(+)” and “(-)” mean?
<4-1-17> The symbols “(+)” and “(-)” refer to the two strands of DNA: the positive (or plus) strand and the negative (or minus) strand, respectively.
<4-1-18> RNA-Seq data is strand-specific, meaning that it can determine which DNA strand was used as a template to produce the RNA. {Depending on protocol of library preparation, not all RNA-Seq data is strand-specific.}
<4-1-19> This allows us to distinguish between RNAs that are transcribed from the positive strand versus the negative strand.
<4-1-20> Which genes are expressed and why?
<4-1-21> EVL: Shows expression peaks on the “(+)” strand, indicating that it's actively transcribed from the positive strand.
<4-1-22> DEGS2: Shows expression peaks on the “(-)” strand, which means it's being transcribed from the negative strand. {This gene is not expressed. Domain knowledge negative strand transcription, which by itself is correct, is used to interpret a false claim “peaks on the ‘(-)’ strand track.”}
<4-1-23> YY1: Displays prominent expression peaks on the “(+)” strand, highlighting its active transcription from the positive strand.
<4-1-24> SLC25A29:
<4-1-25> There are expression peaks on the “(+)” strand, implying transcription from the positive strand. {This gene is on “(-)” strand and is not expressed. Domain knowledge positive strand transcription, which by itself is correct, is used to interpret a false claim “peaks on the ‘(+)’ strand”}
<4-1-26> WARS1:
<4-1-27> There's visible expression on the “(-)” strand, indicating it's being transcribed from the negative strand.
<4-1-28> SLC25A47:
<4-1-29> Has minor peaks on the “(+)” strand, suggesting some level of transcription. {This gene is not expressed; Domain knowledge, transcription level, which by itself is correct, is used to interpret a false claim “minor peaks on the ‘(+)’ strand”}
<4-1-30> WDR25:
<4-1-31> Doesn't show significant expression peaks on either strand, which suggests it might not be actively transcribed in the sampled condition or cell type (GM12878 in this case).
<4-1-32> The expression of genes can be influenced by various factors, including cell type, developmental stage, external stimuli, and regulatory mechanisms.
<4-1-33> In this context, the cell type GM12878 (a lymphoblastoid cell line) might have certain requirements or functions, leading to the expression of specific genes.
<4-1-34> The presence of expression peaks in the RNA-Seq data indicates that these genes are being transcribed into RNA in these cells.
<4-1-35> The exact "why" would require deeper insights into the cell's biology, its role, and the function of each gene in that particular cellular context.
<4-1-36> Would you like to move on to the next section or discuss the expression data further?

Q

3. Check the ChIP-seq data. Explain the association of H3K4me3, H3K27ac, and H3K27me3 with gene expression. Based on the three histone modifications, which genes are likely expressed, and which are not likely expressed?

<4-1-37> 3. ChIP-seq Data and Histone Modifications:.
<4-1-38> H3K4me3 (Tri-methylation of lysine 4 on histone H3): Association with Gene Expression:
<4-1-39> H3K4me3 is typically associated with active transcription start sites (TSS).
<4-1-40> The presence of this mark near the TSS is indicative of genes that are either actively being transcribed or are

poised for transcription.
<4-1-41> H3K27ac (Acetylation of lysine 27 on histone H3): Association with Gene Expression:
<4-1-42> H3K27ac is associated with active enhancers and promoters.
<4-1-43> The presence of this mark, especially in conjunction with H3K4me3, usually signifies gene activation.
<4-1-44> H3K27me3 (Tri-methylation of lysine 27 on histone H3): Association with Gene Expression: H3K27me3 is a repressive mark, often found on genes that are transcriptionally silenced.
<4-1-45> Its presence usually indicates genes that are not currently being transcribed or genes that are kept in a repressed state.
<4-1-46> Based on the three histone modifications:.
<4-1-47> EVL: Shows strong H3K4me3 and H3K27ac peaks near its TSS, suggesting it's likely expressed.
<4-1-48> There's no significant H3K27me3 peak, further supporting its active status.
<4-1-49> DEGS2: Displays pronounced H3K4me3 and H3K27ac peaks, implying it's likely actively transcribed. {No peak of H3K27ac observed. Domain knowledge, H3K27ac indicating transcription, which by itself is correct, is used to interpret a false claim on the H3K27ac presence on DEGS2.}
<4-1-50> The absence of a H3K27me3 mark reinforces this conclusion. {H3K27me3 peak observed.}
<4-1-51> YY1: Has dominant peaks of both H3K4me3 and H3K27ac, indicating it's actively transcribed.
<4-1-52> Again, the lack of H3K27me3 supports this.
<4-1-53> SLC25A29: Shows prominent H3K4me3 and H3K27ac peaks, suggesting it's likely expressed. {No peak for H3K27ac and weak peak for H3K4me3. Domain knowledge, H3K27ac indicating transcription, which by itself is correct, is used to interpret a false claim on the H3K27ac presence on SLCA29.}
<4-1-54> No H3K27me3 peak further endorses its active transcription. {H3K27me3 peak observed.}
<4-1-55> WARS1:
<4-1-56> While there are H3K4me3 and H3K27ac peaks, they aren't as pronounced as for some of the other genes, but it still suggests some level of transcriptional activity. {The peaks for H3K4me3 and H3K27ac are strong and pronounced. Domain knowledge, transcription level, which by itself is correct, is used to interpret a false claim on the less pronounced H3K4me3 and H3K27ac.}
<4-1-57> SLC25A47: Has minor H3K4me3 and H3K27ac peaks, hinting at potential low-level transcription.
<4-1-58> The absence of a significant H3K27me3 peak supports this. {H3K27me3 peak observed.}
<4-1-59> WDR25:
<4-1-60> This gene has minimal signals for H3K4me3 and H3K27ac and displays a noticeable H3K27me3 peak. {Strong signal for H3K4me3 and H3K27ac; No peak observed for H3K27me3.}
<4-1-61> This suggests that WDR25 is not likely expressed and might be in a repressed state.
<4-1-62> In summary, EVL, DEGS2, YY1, SLC25A29, and WARS1 are likely actively expressed. {DEGS2, SLC25A29 are not actively expressed.}
<4-1-63> SLC25A47 might have low-level expression, and WDR25 appears to be not expressed or in a repressed state in GM12878 cells based on the histone modifications. {SLC25A47 is not expressed. WDR25 is not expressed according to RNA-seq data, while histone modification pattern indicates expressed or a poised state for expression.}
<4-1-64> Would you like to delve deeper into another aspect or move on to the next section?

Q

4. Three groups of genomic regions are highlighted: “A”s in light blue, “I”s in pink, and “E”s in green. Identify the number of regions for each group.

<4-1-65> Sure, let's identify the number of regions for each group based on the highlighted colors in the figure:.
<4-1-66> "A" regions (light blue): A1 A2 A3.
<4-1-67> Total: 3 regions.
<4-1-68> "I" regions (pink): I1 I2.
<4-1-69> Total: 2 regions.
<4-1-70> "E" regions (green): E1 E2 E3 E4.
<4-1-71> Total: 4 regions.
<4-1-72> In summary: There are 3 "A" regions highlighted in light blue.
<4-1-73> There are 2 "I" regions highlighted in pink.

<4-1-74> There are 4 "E" regions highlighted in green.
<4-1-75> Would you like to discuss these regions further or move on to another topic?

Q

5. For genomic regions in "A"s, what are their shared features in terms of positional relationship with genes and histone modification patterns? What is their potential function in transcription regulation?

<4-1-76> 5. Features of "A" Regions:.
<4-1-77> Positional Relationship with Genes: A1: Located upstream of the DEGS2 gene and overlaps slightly with its TSS (transcription start site). {A1 has substantial overlap with the region surrounding TSS of EVL, both in upstream and downstream.}
<4-1-78> A2: Positioned within an intronic region of the YY1 gene. {A2 has substantial overlap with the region surrounding TSS of YY1, both in upstream and downstream.}
<4-1-79> A3: Located downstream of the WDR25 gene and overlaps slightly with its TSS. {A3 has substantial overlap with the region surrounding TSSs of WDR25 and WARS1, both in upstream and downstream.}
<4-1-80> Histone Modification Patterns:
<4-1-81> All "A" regions exhibit prominent H3K4me3 peaks.
<4-1-82> They also show peaks of H3K27ac.
<4-1-83> Importantly, none of the "A" regions display the repressive H3K27me3 mark.
<4-1-84> Potential Function in Transcription Regulation:
<4-1-85> Given their positional relationship with genes and the histone modification patterns observed: The presence of H3K4me3, especially overlapping with TSS regions for A1 and A3, strongly suggests that these "A" regions are associated with active promoters of the respective genes (DEGS2 for A1 and WDR25 for A3). {EVL for A1.}
<4-1-86> A2, being within the intronic region of YY1 and exhibiting H3K4me3 and H3K27ac marks, might suggest an intronic enhancer or alternative promoter activity for YY1. {A2 overlaps YY1 promoter. Domain knowledge, "intronic enhancer", which by itself is correct, is used to interpret a false claim on A2 being an intronic region.}
<4-1-87> The presence of H3K27ac in these regions, devoid of the repressive H3K27me3 mark, further supports their role in gene activation.
<4-1-88> H3K27ac is a mark of active enhancers and promoters.
<4-1-89> The combination of H3K4me3 and H3K27ac is a hallmark of active transcriptional regulation, often seen at active promoters or enhancers.
<4-1-90> In conclusion, the "A" regions, based on their positional relationships with genes and their histone modification patterns, likely represent active promoter regions for genes (especially A1 and A3).
<4-1-91> A2 might indicate an intronic enhancer or alternative promoter activity. {A2 is a prompter region.}
<4-1-92> These regions play a pivotal role in transcriptional initiation and are essential for the activation and regulation of their respective or nearby genes.

Q

6. How about genomic regions in "I"s? What is their potential function in transcription regulation?

<4-1-93> 6. Features of "I" Regions:.
<4-1-94> Histone Modification Patterns:
<4-1-95> Both "I" regions (I1 and I2) lack the active marks H3K4me3 and H3K27ac, which are typically associated with active promoters or enhancers. {Both I1 and I2 have minor peaks for H3k4me3.}
<4-1-96> Notably, these regions exhibit a strong presence of the H3K27me3 mark, which is a repressive chromatin modification.
<4-1-97> Potential Function in Transcription Regulation:

<4-1-98> The presence of the H3K27me3 mark suggests that the "I" regions are associated with transcriptional repression or silencing.
<4-1-99> This mark is typically found on chromatin regions that are kept in a condensed, non-permissive state, preventing the transcription of genes in those regions.
<4-1-100> Given their lack of active histone marks (H3K4me3 and H3K27ac) and the prominence of the repressive H3K27me3 mark, the "I" regions likely represent silenced or repressed chromatin domains. {The “I” regions show minor H3K4me3 peaks.}
<4-1-101> These regions might be involved in keeping nearby genes or genomic regions in a repressed state, possibly to prevent erroneous or untimely expression.
<4-1-102> It's also possible that these regions could be involved in long-range chromatin interactions, bringing other distant regulatory elements or genes into proximity for coordinated repression, but this would require further 3D chromatin interaction data for confirmation.
<4-1-103> In conclusion, the "I" regions, based on their histone modification patterns, are likely associated with transcriptional repression or silencing.
<4-1-104> They play a role in ensuring that certain genes or genomic regions remain inactive under specific conditions or in particular cell types.

Q

7. How about genomic regions in “E”s? What is their potential function in transcription regulation?

<4-1-105> 7. Features of "E" Regions:.
<4-1-106> Histone Modification Patterns:
<4-1-107> All "E" regions (E1, E2, E3, and E4) show a strong presence of the H3K27ac mark.
<4-1-108> This modification is commonly associated with active enhancers.
<4-1-109> Importantly, these regions lack significant peaks of the H3K4me3 mark, which is typically associated with active promoters. {H3K4me3 peaks are observed for “E” regions. Enhancers may also have H3K4me3.}
<4-1-110> None of the "E" regions display the repressive H3K27me3 mark, indicating they are not in a repressed state.
<4-1-111> Potential Function in Transcription Regulation:
<4-1-112> The presence of H3K27ac, especially in the absence of H3K4me3, is indicative of enhancer activity.
<4-1-113> Enhancers are distal regulatory elements that can activate gene transcription by looping to bring themselves in proximity to target gene promoters.
<4-1-114> This can occur even if the enhancer is several kilobases away from the promoter.
<4-1-115> Given their histone modification patterns, the "E" regions likely represent active enhancer regions.
<4-1-116> These enhancers can recruit transcriptional co-activators and the basal transcription machinery to facilitate the transcription of target genes.
<4-1-117> They can also establish chromatin loops to physically interact with their target promoters.
<4-1-118> The specific genes they regulate would need to be confirmed with further experimental data, such as Chromatin Conformation Capture (3C) or its derivatives (4C, 5C, Hi-C), which can provide insights into the 3D interactions of these enhancers with potential target promoters.
<4-1-119> In conclusion, the "E" regions, based on their histone modification patterns, likely represent active enhancer elements.
<4-1-120> They play a pivotal role in upregulating the transcription of their target genes, often from a distance, by establishing physical interactions with their respective or nearby gene promoters.

Q

8. On the heatmap for chromatin-chromatin interactions, there are several circles each connected with dashed lines. Explain what the circle means and how the dashed lines are connected to the circle. What are the common features of the circles in terms of indicated genomic region(s)?

<4-1-121> 8. Chromatin-Chromatin Interactions Heatmap:.
<4-1-122> What the Circle Means:
<4-1-123> The circles on the heatmap represent significant chromatin-chromatin interactions.
<4-1-124> In the context of this type of visualization, these circles indicate that the two corresponding genomic regions (on the x and y axes of the heatmap) are interacting with each other more frequently than expected by chance. {There is no y axis needed be defined in the heatmap.}
<4-1-125> Such interactions can suggest that these regions are in physical proximity within the 3D structure of the nucleus, often mediated by protein complexes or other structural elements.
<4-1-126> How the Dashed Lines are Connected to the Circle:
<4-1-127> Each circle is connected by two dashed lines that extend from the circle to the respective axes of the heatmap.{Extend both to the x axis, which defines genomic positions.}
<4-1-128> These dashed lines help identify which two genomic regions are involved in the interaction represented by the circle.
<4-1-129> The point where the dashed line meets an axis corresponds to a specific genomic location.
<4-1-130> By tracing these lines, you can determine which regions are interacting with one another.
<4-1-131> Common Features of the Circles in Terms of Indicated Genomic Region(s):
<4-1-132> Upon observation, many of the circles (or interaction points) seem to involve the "E" regions.
<4-1-133> This is consistent with the notion that "E" regions, which are indicative of enhancers, often participate in long-range chromatin interactions.
<4-1-134> The interaction of these "E" regions with other parts of the genome, especially near gene promoters (like "A" regions), supports the idea that enhancers (E regions) are looping to interact with promoters to regulate gene expression.
<4-1-135> Some circles also appear to involve interactions between "A" regions, suggesting potential promoter-promoter interactions, or possibly shared regulatory mechanisms between nearby genes.
<4-1-136> In summary, the circles on the heatmap represent significant chromatin-chromatin interactions, and the dashed lines help identify the specific genomic regions involved in each interaction.
<4-1-137> Many of the circles indicate interactions involving the "E" regions, consistent with the role of enhancers in establishing long-range interactions to regulate gene expression.

Q

9. There are two groups of circles by color. Identify the number of circles for each group.

<4-1-138> From the provided figure:.
<4-1-139> Purple Circles: {There are no purple circle.}
<4-1-140> Counting the distinct purple circles, I observe: One near the interaction between E1 and I1 One connecting E2 and I1 One between E3 and I2 One linking E4 and I2. {There is no circle for E1::I1, E2::I1, E3::I2, and E4::I2.}
<4-1-141> Total: 4 purple circles. {No purple circle.}
<4-1-142> Blue Circles: {GPT-4V likely meant to refer green circles.}
<4-1-143> Counting the distinct blue circles, I observe: One connecting E1 and A2 One linking E2 and A2 One between E3 and A2 One for the interaction between E4 and A2. {There is no circle for E4::A2.}
<4-1-144> Total: 4 blue circles. {3 green circles.}
<4-1-145> In summary: There are 4 purple circles. {No purple circle here.}
<4-1-146> There are 4 blue circles. {3 green circles.}
<4-1-147> Would you like to explore the significance of the color differentiation or any other aspect of the heatmap?

Q

10. Focus on the circles in blue, and identify their indicated interacting genomic regions.

<4-1-148> Sure, focusing on the blue circles:.
<4-1-149> 1. First Blue Circle: Interacts with: E1 and A2. {GPT-4V likely meant to refer the first green circle, counting from the left.}
<4-1-150> 2.
<4-1-151> Second Blue Circle: Interacts with: E2 and A2. {GPT-4V likely meant to refer the second green circle, counting from the left.}
<4-1-152> 3.
<4-1-153> Third Blue Circle: Interacts with: E3 and A2. {GPT-4V likely meant to refer green circle, counting from the left.}
<4-1-154> 4.
<4-1-155> Fourth Blue Circle: Interacts with: E4 and A2. {GPT-4V likely meant to refer green circle; There is no circle for E4::A2}
<4-1-156> In summary, all blue circles indicate interactions between the respective "E" regions (E1, E2, E3, and E4) and the "A2" region.{3 blue circles}
<4-1-157> This suggests that all these enhancer regions ("E"s) may have a role in regulating or influencing the transcriptional activity associated with the "A2" region, potentially related to the YY1 gene given the position of "A2".

11. Focus on the circles in green, and identify their indicated interacting genomic regions.

<4-1-158> I apologize for the oversight earlier, but from the image you provided, there are no green circles mentioned. {3 green circles}
<4-1-159> We previously identified blue and purple circles. {No purple circles}
<4-1-160> However, if you'd like, I can continue the analysis based on the purple circles or address any other questions you might have. {No purple circles}

12. Are there any specific suggestions to improve the data presentation of the figure?

<4-1-161> Certainly!
<4-1-162> While the figure provides a lot of valuable information, there are always potential improvements that can be made to enhance data presentation and interpretation.
<4-1-163> Here are some suggestions:.
<4-1-164> 1. Legends and Annotations:
<4-1-165> Ensure that all colors, symbols, and tracks have clear and concise legends.
<4-1-166> This helps the viewer to quickly understand the significance of each visual element.
<4-1-167> Provide a brief description or title for each track to give context.
<4-1-168> For example, instead of just labeling tracks as "RNA-Seq (+)" or "RNA-Seq (-)", they could be labeled as "RNA-Seq expression (positive strand)" and "RNA-Seq expression (negative strand)" respectively.
<4-1-169> 2. Heatmap Scaling:
<4-1-170> The heatmap for chromatin-chromatin interactions could benefit from a color scale that indicates the strength of interactions.
<4-1-171> This helps viewers gauge the relative significance of each interaction.
<4-1-172> 3. Clarity of Highlights:
<4-1-173> The highlighted regions ("A", "I", "E") could use a slightly thicker border or a different shading pattern to make them stand out more.
<4-1-174> 4. Expand on Histone Marks:
<4-1-175> For the histone modification tracks, consider providing a brief note or inset explaining the significance of each mark in terms of gene regulation (activating, repressing, etc.).

<4-1-176> 5. Interactive Features (for digital platforms):
<4-1-177> If presented digitally, consider adding interactive features where viewers can hover over specific regions or circles to get more detailed information.
<4-1-178> Zooming capability would be helpful, allowing viewers to focus on specific regions of interest.
<4-1-179> 6. Consistent Coloring:
<4-1-180> Ensure that colors are consistent throughout the figure.
<4-1-181> For instance, if enhancer regions are marked in green in one part of the figure, they should be in green everywhere else as well.
<4-1-182> 7.
<4-1-183> Simplify Complex Regions:
<4-1-184> For particularly dense or complex areas of the figure, consider breaking them into sub-panels or providing an enlarged inset to help viewers understand the details better.
<4-1-185> 8. 3D Interaction Visualization:
<4-1-186> The chromatin-chromatin interaction heatmap provides a 2D representation of 3D interactions.
<4-1-187> It could be helpful to have a supplementary figure or inset showing a schematic 3D representation of how these regions might interact in the spatial organization of the nucleus.
<4-1-188> 9.
<4-1-189> Reference Lines:
<4-1-190> Consider adding subtle reference lines or gridlines to help trace interactions from the heatmap to the genomic coordinates.
<4-1-191> 10.
<4-1-192> Figure Description: Include a brief but comprehensive figure description that guides the reader through each part of the image, explaining the relevance and importance.
<4-1-193> Improving the figure in these ways can make it more accessible, especially for readers who might not be experts in the field, and enhance the overall interpretability of the presented data.

Q

13. Draft a title and a legend for this figure based on our previous discussion. Make sure to include details.

<4-1-194> Title: "Epigenomic Landscape and Chromatin-Chromatin Interactions Surrounding the YY1 Gene in GM12878 Cells".
<4-1-195> Legend:.
<4-1-196> Tracks:
<4-1-197> Genes:
<4-1-198> Represents RefSeq annotated genes with their transcriptional orientation.
<4-1-199> Arrowheads indicate the direction of transcription from 5' to 3'.
<4-1-200> Thicker blocks represent exons, and thin lines indicate introns.
<4-1-201> RNA-Seq (+/-): Strand-specific RNA expression profiles.
<4-1-202> Peaks in the "(+)" track denote transcription from the positive strand, while peaks in the "(-)" track indicate transcription from the negative strand.
<4-1-203> H3K4me3:
<4-1-204> ChIP-seq data highlighting tri-methylation at lysine 4 of histone H3, typically associated with active transcription start sites.
<4-1-205> H3K27ac: ChIP-seq data indicating acetylation at lysine 27 of histone H3, a mark of active enhancers and promoters.
<4-1-206> H3K27me3: ChIP-seq data showing tri-methylation at lysine 27 of histone H3, a repressive mark indicating transcriptionally silenced regions.
<4-1-207> Highlighted Regions: A (Light Blue): Putative active promoter regions, often characterized by the presence of H3K4me3 and H3K27ac.
<4-1-208> I (Pink): Potential repressive or silenced domains, typically marked by H3K27me3.
<4-1-209> E (Green): Predicted enhancer regions, often showing enrichment for H3K27ac.
<4-1-210> Heatmap:

<4-1-211> Represents chromatin-chromatin interactions.
<4-1-212> Darker shades indicate stronger interactions.
<4-1-213> Purple Circles: Signify significant interactions between enhancer ("E") regions and repressive ("I") domains. {There is no purple circle.}
<4-1-214> Blue Circles: Denote significant interactions between enhancer ("E") regions and active promoter ("A") domains. {GPT-4V likely meant to refer green circle.}
<4-1-215> This legend, coupled with the figure, aims to provide a comprehensive view of the epigenomic data and chromatin interactions in the vicinity of the YY1 gene in GM12878 cells.

14. Draft a paragraph to describe the findings from our previous discussion on transcription regulation of YY1. This is one paragraph for the result part of a manuscript. 150 words or less.

<4-1-216> In the GM12878 cell line, the transcriptional regulation of the YY1 gene has been elucidated through an integrated analysis of epigenomic landscapes and chromatin-chromatin interactions.
<4-1-217> Our data revealed three active promoter regions (A1, A2, and A3) marked by prominent H3K4me3 and H3K27ac peaks.
<4-1-218> Specifically, region A2 within the intronic span of YY1 indicates potential intronic enhancer or alternative promoter activity. {A2 marks the promoter region of YY1. Domain knowledge, "intronic enhancer", which by itself is correct, is used to interpret a false claim on A2 being an intronic region.}
<4-1-219> Four putative enhancer regions (E1-E4) enriched for H3K27ac were identified, suggesting active enhancer functions.
<4-1-220> Notably, chromatin interaction analyses showcased that all these "E" regions establish significant interactions with the A2 region, indicating a likely coordinated regulatory mechanism for YY1 transcription. {E1 to E3 but not E4 are indicated to interact with A2. Domain knowledge, coordinated regulation, which by itself is correct, is used to interpret a false claim on all "E" regions interacting with A2.}
<4-1-221> Additionally, two domains (I1 and I2) marked predominantly by the repressive H3K27me3 modification were detected, hinting at regions of transcriptional silencing.
<4-1-222> Together, these findings underscore a complex interplay of enhancers, promoters, and repressed domains orchestrating the transcriptional regulation of YY1 in GM12878 cells.

The “YY1” case study (in-depth assessment 2)

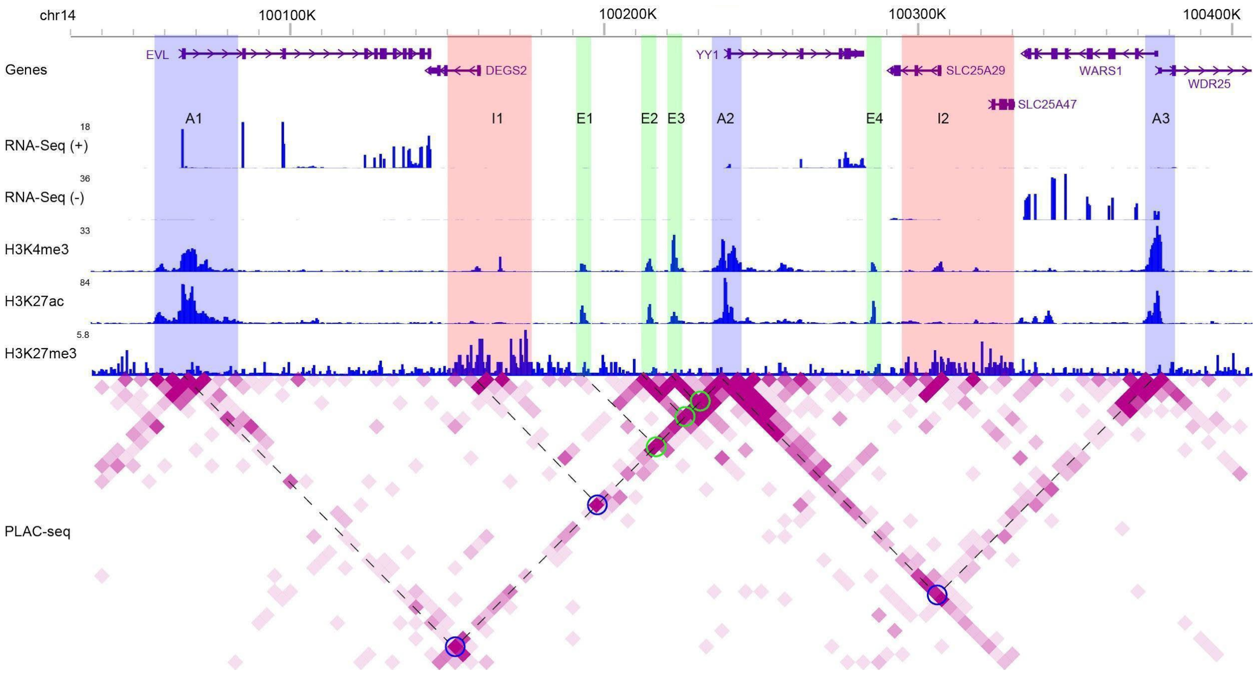

Acting as a bioinformatics professional proficient in RNA-Seq, ChIP-seq, and 3D chromatin analysis, you will help interpret a figure to appreciate transcription regulation at the chromatin level for YY1 in GM12878. The attached figure is a screenshot from the WashU genome browser, encompassing gene annotation, expression data, ChIP-Seq data, and chromatin-chromatin interaction data, for a genomic region enclosing YY1. The aim is to infer transcriptional regulatory mechanisms for YY1 from the epigenomic data. You will be guided with questions to identify findings addressing the aim. Please do the following.

1. Go over the RefSeq gene track and spell out the genes shown on the plot. Explain the presentation of gene structure. For example, what does the arrowhead mean?

<4-2-1> Sure, let's dive into the figure:.

<4-2-2> 1. RefSeq Gene Track & Gene Structure: Genes Shown on the Plot:

<4-2-3> The genes displayed in this region are EVL, DEGS2, YY1, SLC25A29, WARS1, SLC25A47, and WDR25. {SLC25A47 comes before WARS1.}

<4-2-4> Presentation of Gene Structure:

<4-2-5> The gene structure is represented by horizontal lines and arrowheads.

<4-2-6> Horizontal Lines:

<4-2-7> These lines represent introns.

<4-2-8> Boxes or Rectangles:

<4-2-9> These typically represent exons.

<4-2-10> Exons are the coding sequences of the gene.

<4-2-11> Arrowheads:

<4-2-12> These indicate the direction of transcription or the orientation of the gene.

<4-2-13> If the arrowhead points to the right, the gene is transcribed from left to right on the forward strand.

<4-2-14> If the arrowhead points to the left, the gene is transcribed from right to left on the reverse strand.

<4-2-15> From the figure, for example: The YY1 gene has arrowheads pointing to the right, indicating it's transcribed from left to right on the forward strand.

<4-2-16> The EVL gene has arrowheads pointing to the left, suggesting it's transcribed from right to left on the reverse strand. {Arrow heads for EVL pointing to the right not the left.}

<4-2-17> The visualization gives us an understanding of the gene's exon-intron structure, its orientation, and its relative

position to other genes in the genomic landscape.
<4-2-18> This is crucial for understanding potential regulatory mechanisms and interactions between different genes and regulatory elements.

Q

2. Check the expression data. What do “(+)” and “(-)” mean? Which genes are expressed and why?

<4-2-19> 2. Expression Data (RNA-Seq):.
<4-2-20> (+) and (-): These denote the two strands of DNA.
<4-2-21> (+): Refers to the positive or forward strand.
<4-2-22> (-): Refers to the negative or reverse strand.
<4-2-23> The RNA-Seq data provides information on the expression of genes based on the sequencing of RNA.
<4-2-24> In the context of this figure, peaks or bars seen in the RNA-Seq tracks represent regions where RNA has been detected and thus, where genes are being transcribed.
<4-2-25> Which genes are expressed and why?
<4-2-26> EVL:
<4-2-27> There are peaks in the RNA-Seq (-) track, indicating that EVL is expressed and is transcribed from the reverse strand. {Peaks are in (+) track, so transcribed from the forward strand. Domain knowledge “reverse strand”, which by itself is correct, is used to interpret a false claim “peaks in the RNA-Seq (-) track.”}
<4-2-28> DEGS2:
<4-2-29> There are peaks in the RNA-Seq (-) track, indicating that DEGS2 is expressed and is transcribed from the reverse strand. {There is no RNA-Seq peak for DEGS2. This gene is not expressed. Domain knowledge “reverse strand”, which by itself is correct, is used to interpret a false claim “peaks in the RNA-Seq (-) track.”}
<4-2-30> YY1:
<4-2-31> There are pronounced peaks in the RNA-Seq (+) track, which means YY1 is actively expressed and is transcribed from the forward strand.
<4-2-32> SLC25A29, WARS1, SLC25A47, and WDR25:
<4-2-33> These genes also show peaks in their respective RNA-Seq tracks, indicating their expression. {SLC25A29, SLC25A47, and WDR25 are not expressed}
<4-2-34> The why part of the question is more nuanced.
<4-2-35> The exact reason for gene expression depends on cellular context, regulatory mechanisms, and other factors.
<4-2-36> However, based on the data given: Genes that show peaks in the RNA-Seq data are actively transcribed in the GM12878 cell line under the conditions the data was collected.
<4-2-37> The presence of regulatory marks (as shown in other tracks, e.g., H3K27ac, which is a mark of active enhancers) can also provide clues as to why a gene is being expressed.
<4-2-38> In summary, the RNA-Seq data demonstrates that EVL, DEGS2, YY1, SLC25A29, WARS1, SLC25A47, and WDR25 are expressed in the GM12878 cell line in this specific genomic context. {DEGS2, SLC25A29, SLC25A47, and WDR25 are not expressed}
<4-2-39> The exact regulatory mechanisms driving their expression can be inferred by looking at the combination of chromatin modifications, chromatin-chromatin interactions, and other epigenomic data present in the figure.

Q

3. Check the ChIP-seq data. Explain the association of H3K4me3, H3K27ac, and H3K27me3 with gene expression. Based on the three histone modifications, which genes are likely expressed, and which are not likely expressed?

<4-2-40> 3. ChIP-seq Data & Histone Modifications:.
<4-2-41> H3K4me3 (Tri-methylation of lysine 4 on histone H3): Association with Gene Expression:
<4-2-42> H3K4me3 is typically found near the transcription start site (TSS) of active genes.
<4-2-43> It is a mark associated with active promoters and is correlated with gene transcription.

<4-2-44> A strong presence of H3K4me3 is a good indication that the corresponding gene is actively transcribed.
<4-2-45> H3K27ac (Acetylation of lysine 27 on histone H3): Association with Gene Expression:
<4-2-46> H3K27ac is associated with active enhancers and promoters.
<4-2-47> Its presence indicates that a genomic region is involved in active transcriptional regulation.
<4-2-48> Enhancers with H3K27ac can activate gene expression from a distance.
<4-2-49> H3K27me3 (Tri-methylation of lysine 27 on histone H3): Association with Gene Expression:
<4-2-50> H3K27me3 is a repressive mark.
<4-2-51> It is associated with transcriptional silencing and is typically found covering extended regions of inactive genes.
<4-2-52> If a gene's promoter is enriched with H3K27me3, it is likely that the gene is repressed or not actively transcribed.
<4-2-53> Based on the three histone modifications:.
<4-2-54> Likely Expressed:
<4-2-55> YY1: Shows strong H3K4me3 and H3K27ac signals near its TSS, indicating it is actively transcribed.
<4-2-56> EVL: Has H3K4me3 and H3K27ac marks, suggesting it is actively transcribed.
<4-2-57> DEGS2: Presents H3K4me3 and H3K27ac signals, indicating active transcription. {No H3K27ac peak. The domain knowledge, H3K27ac indicating expression, which by itself is correct, is used to interpret a false observation the presence of H3K27ac on DEGS2.}
<4-2-58> WARS1:
<4-2-59> Although the H3K4me3 signal is not very pronounced, it does display H3K27ac, suggesting possible active transcription. {The H3K4me3 peak is very pronounced.}
<4-2-60> SLC25A29, SLC25A47, and WDR25:
<4-2-61> These genes also have indications of H3K27ac, suggesting potential transcriptional activity, though the evidence is not as strong as for YY1, EVL, and DEGS2. {SLC25A29, SLC25A47, and DEGS2 do not have H3K27ac peaks. The domain knowledge, H3K27ac indicating expression, which by itself is correct, is used to interpret a false observation the presence of H3K27ac on SLC25A29 and SLC25A47.}
<4-2-62> Not Likely Expressed or Repressed:
<4-2-63> Any gene that displays H3K27me3 over its TSS or gene body could be considered repressed.
<4-2-64> From the provided image, no gene is overtly covered with H3K27me3, so none of the genes seem to be repressed based on this mark. {DEGS2, SLC25A29, and SLC25A47 have their promoters marked by H3K27me3. The domain knowledge, H3K27me3 indicating repression, which by itself is correct, is used to interpret a false observation the total absence of H3Kme3 across all genes.}
<4-2-65> In summary, based on the histone modifications, YY1, EVL, DEGS2, and possibly WARS1, SLC25A29, SLC25A47, and WDR25 are likely actively transcribed. {DEGS2, SLC25A29, SLC25A47 and WDR25 are not expressed}
<4-2-66> There isn't any gene that is clearly repressed based on H3K27me3 from the given data. {DEGS2, SLC25A29, and SLC25A47 have their promoters marked by H3K27me3.}

Q

4. Three groups of genomic regions are highlighted: “A”s in light blue, “I”s in pink, and “E”s in green. Identify the number of regions for each group.

<4-2-67> 4. Highlighted Genomic Regions:.
<4-2-68> A (Light Blue): There are three regions highlighted in light blue, labeled as A1, A2, and A3.
<4-2-69> I (Pink): There are two regions highlighted in pink, labeled as I1 and I2.
<4-2-70> E (Green): There are four regions highlighted in green, labeled as E1, E2, E3, and E4.
<4-2-71> In summary: There are 3 "A" regions.
<4-2-72> There are 2 "I" regions.
<4-2-73> There are 4 "E" regions.

Q

5. For genomic regions in “A”s, what are their shared features in terms of positional relationship with genes and histone modification patterns? What is their potential function in transcription regulation?

<4-2-74> 5. Genomic Regions in "A" (Light Blue) Features & Potential Function:.
<4-2-75> Positional Relationship with Genes:
<4-2-76> All "A" regions (A1, A2, A3) are positioned over the gene bodies or near the transcription start site (TSS) of genes. {All "A" regions covered TSS and nearby regions in the upstream and downstream. The mention of gene bodies and TSS is correct though.}
<4-2-77> They do not appear to be distal from genes, suggesting they are closely associated with the genes they overlay.
<4-2-78> Histone Modification Patterns:
<4-2-79> All three "A" regions show pronounced H3K4me3 peaks, which, as previously mentioned, is a histone modification associated with active promoters and active transcription.
<4-2-80> There is no noticeable presence of the repressive mark H3K27me3 in the "A" regions.
<4-2-81> H3K27ac, a mark of active enhancers and promoters, can be seen, especially in A1 and A3 regions. {H3K27ac in A2 region is as prominently as A1 and A3. The use of domain knowledge to explain H3K27ac is correct though.}
<4-2-82> Potential Function in Transcription Regulation:
<4-2-83> Given their positional relationship and histone modification patterns, the "A" regions are likely to be active promoters or gene regulatory regions.
<4-2-84> Their proximity to the TSS of genes and the presence of H3K4me3 suggest that these regions might be directly involved in the initiation of transcription.
<4-2-85> The presence of H3K27ac further supports the idea that these regions are transcriptionally active.
<4-2-86> In essence, the "A" regions appear to be crucial for the transcriptional initiation of the genes they are associated with, acting as active promoters or regulatory regions that facilitate gene transcription.

Q

6. How about genomic regions in "I"s? What is their potential function in transcription regulation?

<4-2-87> 6. Genomic Regions in "I" (Pink) Potential Function:.
<4-2-88> Positional Relationship with Genes:
<4-2-89> The "I" regions (I1 and I2) are interspersed between genes or are located within intronic regions of genes.
{They overlap with promoters of DEGS2 and span a region covering SLC25A29 and SLC25A47.}
<4-2-90> Histone Modification Patterns:
<4-2-91> Neither of the "I" regions shows a pronounced H3K4me3 peak, indicating they are not typical active promoters.
<4-2-92> They also lack strong H3K27ac signals, suggesting they may not be active enhancers in the traditional sense.
<4-2-93> No noticeable presence of the repressive mark H3K27me3, indicating they are not repressed regions. {The regions are featured by H3K27me3. The domain knowledge, H3K27me3 indicating repression, which by itself is correct, is used to interpret a false observation the absence of H3K27me3 across in "I" regions.}
<4-2-94> Potential Function in Transcription Regulation:
<4-2-95> Given their location and absence of traditional promoter or enhancer marks, the "I" regions might function as:
Intronic Enhancers: {The location of these region overlaps with gene promoters. So these regions are promoters. Note that inactive promoter may not have traditional promoter marker such as H3K4me3. The domain knowledge "intronic enhancer", which by itself is correct, is used to interpret a false observation the absence of promoters in "I" regions.}
<4-2-96> Some introns can contain enhancers that regulate the expression of their host gene or even distal genes. {The domain knowledge on intronic enhancer, which by itself is correct, is used to interpret a false observation the absence of promoters in "I" regions.}
<4-2-97> Splicing Regulators:
<4-2-98> Being intronic, they might play roles in alternative splicing, influencing which exons are included in the mature mRNA. {The domain knowledge on intronic enhancer, which by itself is correct, is used to interpret a false observation the absence of promoters in "I" regions.}

<4-2-99> Structural or Topological Roles:
<4-2-100> They might play roles in the 3D structure of the chromatin, influencing how genes are positioned relative to their regulatory elements. {The domain knowledge on intronic enhancer, which by itself is correct, is used to interpret a false observation the absence of promoters in “I” regions.}
<4-2-101> In conclusion, the "I" regions seem to represent non-traditional regulatory elements that might function as intronic enhancers, splicing regulators, or play roles in chromatin topology. {“I” regions covered promoters of silenced genes marked by H3K27me3. The domain knowledge “intronic enhancer” or “splicing regulators”, which by itself are correct, is used to interpret a false observation the absence of promoters in “I” regions.}
<4-2-102> Their precise function would require further experimental validation, but their intronic position and epigenetic marks suggest a regulatory role that is distinct from classic promoters or enhancers. {“I” regions covered promoters of silenced genes marked by H3K27me3. The domain knowledge “intronic”, which by itself are correct, is used to interpret a false observation the absence of promoters in “I” regions.}

Q

7. How about genomic regions in “E”s? What is their potential function in transcription regulation?

<4-2-103> 7. Genomic Regions in "E" (Green) Potential Function:.
<4-2-104> Positional Relationship with Genes:
<4-2-105> The "E" regions (E1, E2, E3, and E4) are located between genes, suggesting they might be distal regulatory elements.
<4-2-106> Histone Modification Patterns:
<4-2-107> The "E" regions lack pronounced H3K4me3 peaks, indicating they are not typical active promoters.
<4-2-108> They do show the presence of H3K27ac signals.
<4-2-109> As mentioned before, H3K27ac is a hallmark of active enhancers, suggesting that these regions are involved in transcriptional activation.
<4-2-110> No significant presence of the repressive mark H3K27me3 is observed, indicating they aren't silenced or repressed regions.
<4-2-111> Potential Function in Transcription Regulation:
<4-2-112> Given their epigenetic marks and location: Enhancers: The presence of H3K27ac strongly suggests that the "E" regions function as active enhancers.
<4-2-113> Enhancers are DNA sequences that can increase the transcription of genes, and they can do so even if they are located far away from the target gene's promoter.
<4-2-114> Looping Mediators:
<4-2-115> Enhancers often function by looping the DNA to come into close contact with the promoters of the genes they regulate.
<4-2-116> This allows transcription factors and co-activators bound at the enhancer to influence the initiation of transcription at the promoter.
<4-2-117> In conclusion, the "E" regions are likely functioning as active enhancers that influence the transcription of nearby or even distally located genes.
<4-2-118> The presence of H3K27ac in these regions is a strong indication of their enhancer activity.
<4-2-119> They play pivotal roles in increasing the transcriptional output of their target genes, potentially by mediating chromatin looping to bring genes in proximity to their regulatory machinery.

Q

8. On the heatmap for chromatin-chromatin interactions, there are several circles each connected with dashed lines. Explain what the circle means and how the dashed lines are connected to the circle. What are the common features of the circles in terms of indicated genomic region(s)?

<4-2-120> 8. Heatmap for Chromatin-Chromatin Interactions:.
<4-2-121> The Circle:
<4-2-122> The circles on the heatmap represent significant chromatin-chromatin interactions.
<4-2-123> In the context of such heatmaps, these interactions often denote that two distant genomic regions come into close spatial proximity within the cell nucleus, implying a functional interaction between them.
<4-2-124> This is a result of the 3D folding and organization of chromatin, allowing distal regions of the genome to physically interact.
<4-2-125> The Dashed Lines:
<4-2-126> The dashed lines connect the circle on the heatmap to the linear genomic coordinates.
<4-2-127> This visually demonstrates which specific genomic regions are involved in the interaction.
<4-2-128> It's a way to map the 2D interaction (on the heatmap) back to the 1D genome sequence.
<4-2-129> Common Features of the Circles in terms of Indicated Genomic Region(s):
<4-2-130> Most of the circles, if not all, appear to be connected to one of the highlighted regions (A, I, or E).
<4-2-131> This suggests that these highlighted regions are key nodes or hubs of chromatin-chromatin interactions in this genomic landscape.
<4-2-132> Many of the interactions seem to involve an "E" region, indicating that these potential enhancer regions might be actively engaging with other parts of the genome, potentially bringing enhancers in proximity to target gene promoters to regulate transcription.
<4-2-133> In summary, the circles on the heatmap indicate significant chromatin interactions, and the dashed lines connect these interactions back to their respective genomic locations.
<4-2-134> The consistent involvement of the highlighted regions, especially the "E" regions, in these interactions underscores their potential importance in the 3D organization of the genome and in the transcriptional regulation of the encompassed genes.

Q

9. There are two groups of circles by color. Identify the number of circles for each group.

<4-2-135> Based on the provided image:.
<4-2-136> Purple Circles: {There is no purple circle.}
<4-2-137> I can identify three distinct purple circles on the heatmap. {There is no purple circle.}
<4-2-138> Green Circles:
<4-2-139> I can identify two distinct green circles on the heatmap. {3 green circles}
<4-2-140> In summary: There are 3 purple circles. {There is no purple circle.}
<4-2-141> There are 2 green circles. {3 green circles}

Q

10. Focus on the circles in blue, and identify their indicated interacting genomic regions.

<4-2-142> 10. Blue Circles & Their Indicated Interacting Genomic Regions:.
<4-2-143> Upon examining the provided image, there seems to be a small oversight.
<4-2-144> The previously mentioned circles were identified as purple and green. {There is no purple color.}
<4-2-145> There don't appear to be any distinctly "blue" circles on the heatmap. {There are 3 Blue circles.}
<4-2-146> If by "blue" you are referring to the "purple" circles mentioned earlier, here are the indicated interactions:
<4-2-147> 1. The first purple circle seems to indicate an interaction between the A1 region and the E2 region. {There is no circle for A1::E2.}
<4-2-148> 2.
<4-2-149> The second purple circle appears to connect the A3 region with the E3 region. {There is no circle for A1::E2.}

<4-2-150> 3.
<4-2-151> The third purple circle suggests an interaction between the A3 region and another region located between E4 and I2 (the exact label isn't clear from the screenshot). {There is no circle for A3 and the region located between E4 and I2.}
<4-2-152> In summary, based on the provided image and assuming you meant the purple circles: A1 interacts with E2. {See annotation to 4-2-147.}
<4-2-153> A3 interacts with E3. {See annotation to 4-2-149.}
<4-2-154> A3 also interacts with a region between E4 and I2. {See annotation to 4-2-151.}

Q

11. Focus on the circles in green, and identify their indicated interacting genomic regions.

<4-2-155> 11. Green Circles & Their Indicated Interacting Genomic Regions:.
<4-2-156> For the green circles present on the heatmap:.
<4-2-157> 1. The first green circle indicates an interaction between the E1 region and the E3 region. {The first circle counting from left to right marks E1::A2.}
<4-2-158> 2.
<4-2-159> The second green circle suggests an interaction between the E3 region and the E4 region. {The second circle counting from left to right marks E2::A2. GPT-4V may refer this to the last green circle. If so, it marks E3::A2 other than E3::E4 as GPT-4 indicated. In both cases, GPT-4V failed.}
<4-2-160> In summary, for the green circles: E1 interacts with E3. {There is no circle for E1::E3.}
<4-2-161> E3 interacts with E4. {There is no circle for E3::E4.}

Q

12. Are there any specific suggestions to improve the data presentation of the figure?

<4-2-162> 12. Suggestions to Improve Data Presentation of the Figure:. 1. Legend & Annotation:
<4-2-163> A clear legend explaining each color, symbol, and pattern would be beneficial.
<4-2-164> While the figure is detailed, it assumes the viewer understands the nuances of each track.
<4-2-165> The heatmap interactions (circles) should be clearly labeled or given a legend to differentiate between different types (e.g., purple vs. green interactions). {There is no purple color}
<4-2-166> 2. Scale & Resolution:
<4-2-167> The figure might benefit from a higher resolution, especially for reading small labels or discerning subtle differences in peak heights.
<4-2-168> Including a scale or reference for peak heights, especially in RNA-Seq and ChIP-Seq tracks, could help quantify the intensity of signals.
<4-2-169> 3. Highlight Consistency:
<4-2-170> Ensure that the highlighted regions (A, I, E) have consistent coloring throughout.
<4-2-171> Any slight variation in shading might be misconstrued as significant.
<4-2-172> 4. Heatmap Clarity:
<4-2-173> The heatmap for chromatin-chromatin interactions could use clearer delineation or gridlines to better identify the exact regions of interaction.
<4-2-174> Enhance the contrast or color differentiation for significant interactions to make them pop out more against the background.
<4-2-175> 5. Expand on Annotations:
<4-2-176> Additional annotations or labels for specific features might be helpful.
<4-2-177> For instance, if a specific peak or interaction is of particular importance, it could be labeled or pointed out.
<4-2-178> 6.
<4-2-179> Contextual Information:

<4-2-180> A brief title or description above the figure can provide quick context.
<4-2-181> For instance, "Chromatin landscape around the YY1 gene in GM12878 cells" gives immediate clarity.
<4-2-182> Incorporating a brief methods or data source section below the figure can help readers understand the origin of the data and any processing or normalization applied.
<4-2-183> 7. Interactive Features (for online presentations):
<4-2-184> If presented online or in a digital format, consider making the figure interactive.
<4-2-185> This can allow users to zoom in on regions of interest, click on specific elements for more information, or even toggle certain tracks on and off.
<4-2-186> Incorporating these suggestions can enhance the figure's clarity, making it more accessible to a wider audience, and ensuring that key findings are readily apparent to viewers.

Q

13. Draft a title and a legend for this figure based on our previous discussion. Make sure to include details.

<4-2-187> 13.
<4-2-188> Title and Legend for the Figure:.
<4-2-189> ---.
<4-2-190> Title: "Chromatin Landscape and Interactions Surrounding the YY1 Gene in GM12878 Cells".
<4-2-191> ---.
<4-2-192> Legend:.
<4-2-193> Tracks: RefSeq Gene Track:
<4-2-194> Displays the gene structure with horizontal lines representing introns, boxes indicating exons, and arrowheads showing the direction of transcription.
<4-2-195> RNA-Seq (+/-): Represents RNA expression data with peaks indicating active transcription.
<4-2-196> (+) signifies the forward strand, and (-) denotes the reverse strand.
<4-2-197> H3K4me3: Tri-methylation of lysine 4 on histone H3.
<4-2-198> A marker for active promoters.
<4-2-199> H3K27ac: Acetylation of lysine 27 on histone H3.
<4-2-200> Indicates active enhancers and promoters.
<4-2-201> H3K27me3: Tri-methylation of lysine 27 on histone H3.
<4-2-202> A repressive mark associated with gene silencing.
<4-2-203> PLAC-seq: Heatmap showing chromatin-chromatin interactions.
<4-2-204> Darker colors or circles indicate regions of significant interaction.
<4-2-205> Highlighted Regions:
<4-2-206> A (Light Blue): Active promoters or gene regulatory regions associated with the TSS of genes.
<4-2-207> I (Pink): Intronic or interspersed regions, potentially playing roles in alternative splicing, intronic enhancers, or chromatin structure. {The interpretation on "I" regions are misleading. "I" denotes chromatin domains marked by the repressive marker H3K27me3. The domain knowledge "intronic enhancer" or "alternative splicing", which by itself are correct, is used to interpret a false observation the absence of promoters in "I" regions.}
<4-2-208> E (Green): Likely enhancer regions, situated distally from gene bodies and involved in transcriptional activation of genes.
<4-2-209> Heatmap Interactions:
<4-2-210> Purple Circles: {No purple circles}
<4-2-211> Indicate significant chromatin interactions involving 'A' regions.
<4-2-212> Green Circles: Denote significant interactions between different 'E' regions. { Green circles, enhancers (E1 to E3) interacting with YY1 promoter (A2)}
<4-2-213> Note: The heatmap's interactions represent the 3D folding and organization of chromatin, where distant genomic regions come into close spatial proximity within the cell nucleus.
<4-2-214> --.
<4-2-215> This title and legend aim to provide an organized and comprehensive overview of the figure, with detailed explanations of each feature, ensuring clarity for readers.

14. Draft a paragraph to describe the findings from our previous discussion on transcription regulation of YY1. This is one paragraph for the result part of a manuscript. 150 words or less.

<4-2-216> 14. Description of Findings:.

<4-2-217> In the GM12878 cell line, our examination of the chromatin landscape surrounding the YY1 gene unveiled intricate transcriptional regulatory mechanisms.

<4-2-218> Active transcription at the YY1 locus was evidenced by pronounced RNA-Seq peaks, complemented by the presence of H3K4me3 and H3K27ac, hallmark modifications of active promoters and enhancers, respectively.

<4-2-219> Significantly, distal regulatory elements, denoted as "E" regions, showcased H3K27ac enrichment, suggesting their role as active enhancers.

<4-2-220> Notably, chromatin-chromatin interaction heatmaps revealed that these enhancers, especially E1 and E3, are in close spatial proximity, hinting at cooperative transcriptional upregulation. {E1 and E3 do not interact per PLAC-seq. The domain knowledge "cooperative transcriptional upregulation", which by itself is correct, is used to interpret a false claim on E1::E3 interaction.}

<4-2-221> Concurrently, regions labeled "A," proximal to gene transcription start sites, indicate potential promoter activities, given their H3K4me3 enrichment.

<4-2-222> Intriguingly, interspersed "I" regions, despite their intronic positioning, may be regulatory hotspots, potentially affecting alternative splicing or acting as intronic enhancers. {They are promoter regions. The domain knowledge "regulatory hotspots", which by itself is correct, is used to interpret a false observation the absence of promoters in "I" regions.}

<4-2-223> Collectively, this intricate web of interactions and modifications underscores the multifaceted regulation governing YY1 transcription in GM12878 cells.

The “YY1” case study (in-depth assessment 3)

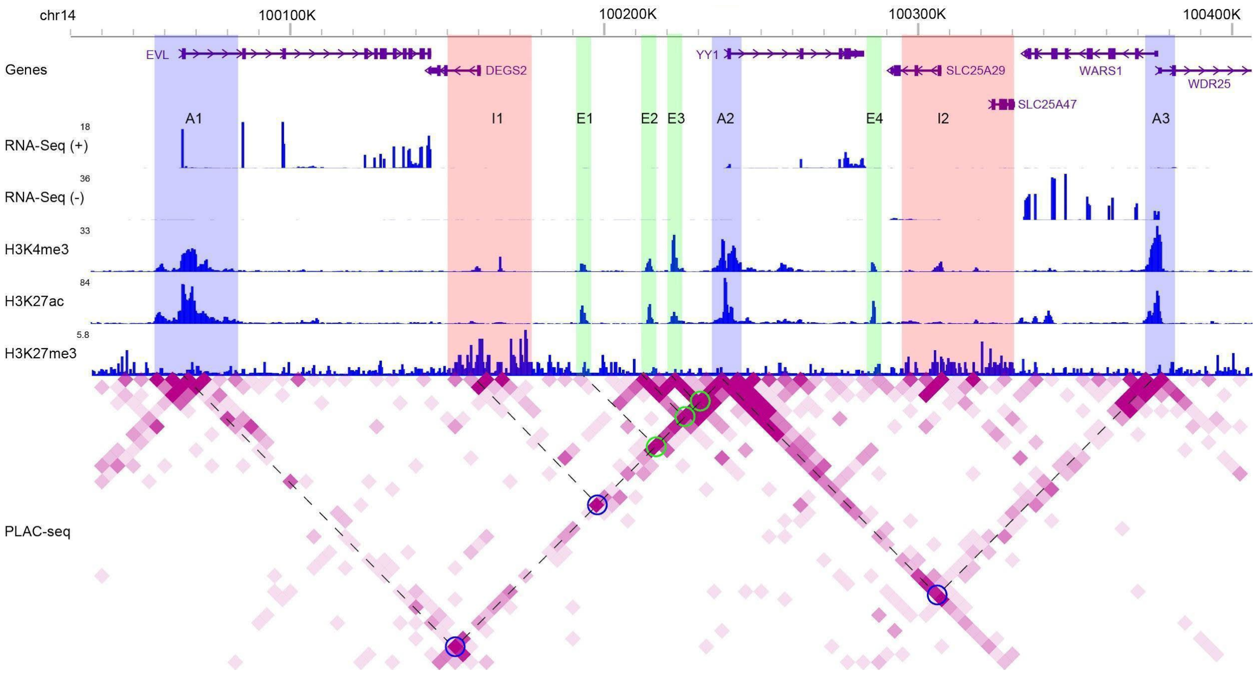

Acting as a bioinformatics professional proficient in RNA-Seq, ChIP-seq, and 3D chromatin analysis, you will help interpret a figure to appreciate transcription regulation at the chromatin level for YY1 in GM12878. The attached figure is a screenshot from the WashU genome browser, encompassing gene annotation, expression data, ChIP-Seq data, and chromatin-chromatin interaction data, for a genomic region enclosing YY1. The aim is to infer transcriptional regulatory mechanisms for YY1 from the epigenomic data. You will be guided with questions to identify findings addressing the aim. Please do the following.

1. Go over the RefSeq gene track and spell out the genes shown on the plot. Explain the presentation of gene structure. For example, what does the arrowhead mean?

<4-3-1> 1. RefSeq Gene Track:
<4-3-2> The RefSeq gene track presents the gene annotation for the given genomic region.
<4-3-3> From the screenshot provided, the genes shown on this plot are:.
<4-3-4> EVL DEGS2 YY1 SLC25A29 SLC25A47 WARS1 WDR25.
<4-3-5> Presentation of Gene Structure:
<4-3-6> The presentation provides an intuitive representation of the gene's structure and direction of transcription.
<4-3-7> .
<4-3-8> Exons:
<4-3-9> Exons are represented by rectangular blocks.
<4-3-10> The size of the block indicates the relative size of the exon.
<4-3-11> Introns:
<4-3-12> The lines connecting the rectangular blocks represent introns, which are non-coding regions between exons.
<4-3-13> Arrowheads:
<4-3-14> The arrowheads (or arrows) on the lines indicate the direction of transcription.
<4-3-15> If the arrowheads point to the right, it means the gene is transcribed from left to right (on the positive strand).
<4-3-16> If they point to the left, the gene is transcribed from right to left (on the negative strand).
<4-3-17> For instance, the gene "EVL" has arrowheads pointing to the left, indicating that it is transcribed from right to left on the negative strand. {EVL is pointing to the right.}
<4-3-18> This directional representation helps understand the orientation of the gene within the genome, which is essential for various genomic analyses and understanding gene regulation.

2. Check the expression data. What do “(+)” and “(-)” mean? Which genes are expressed and why?

<4-3-19> 2. Expression Data:
<4-3-20> The expression data is presented as RNA-Seq tracks, which depict the number of sequencing reads mapped to a particular genomic location.
<4-3-21> This gives an insight into the expression levels of genes in that region.
<4-3-22> (+) and (-) symbols:
<4-3-23> The "(+)" and "(-)" symbols refer to the two different strands of DNA:
<4-3-24> (+) represents the positive or forward strand (also known as the sense strand).
<4-3-25> (-) represents the negative or reverse strand (also known as the antisense strand).
<4-3-26> These tracks show the RNA sequencing reads that align to each strand, respectively.
<4-3-27> When a gene is expressed, the RNA derived from that gene (mRNA) gets sequenced in an RNA-Seq experiment, and these sequences (or reads) then get aligned back to the genome to infer the expression level.
<4-3-28> Expressed Genes:
<4-3-29> Based on the RNA-Seq data presented in the plot: EVL has noticeable expression on the negative strand (as indicated by the peaks in the RNA-Seq (-) track). {It is on positive strand. Domain knowledge negative strand transcription, which by itself is correct, is used to interpret a false claim “peaks on the RNA-Seq (-) track.”}
<4-3-30> YY1 has prominent expression on the positive strand (as evidenced by the peaks in the RNA-Seq (+) track).
<4-3-31> WARS1 also shows expression on the negative strand.
<4-3-32> The other genes (DEGS2, SLC25A29, SLC25A47, and WDR25) have relatively lower or negligible expression, as evidenced by the lack of significant peaks in their corresponding regions.
<4-3-33> Why Are These Genes Expressed?:
<4-3-34> Expression levels of genes can be influenced by various factors, including cell type, developmental stage, external stimuli, and underlying regulatory mechanisms (e.g., transcription factors binding, chromatin state).
<4-3-35> The screenshot provided is from GM12878 cells, which is a lymphoblastoid cell line.
<4-3-36> Thus, the genes expressed (like YY1) might be playing roles relevant to the functions or identity of this cell type.
<4-3-37> The presence of certain histone modifications (like H3K27ac and H3K4me3, which are associated with active transcription) near the promoter regions of these genes can also hint at their active transcriptional status.
<4-3-38> For example, we can see peaks for H3K27ac and H3K4me3 near the YY1 gene, supporting its active transcription.

3. Check the ChIP-seq data. Explain the association of H3K4me3, H3K27ac, and H3K27me3 with gene expression. Based on the three histone modifications, which genes are likely expressed, and which are not likely expressed?

<4-3-39> 3. ChIP-seq Data and Histone Modifications:.
<4-3-40> Histone modifications play a pivotal role in regulating gene expression by affecting chromatin structure and the accessibility of the DNA to transcriptional machinery.
<4-3-41> The three histone modifications presented in the figure – H3K4me3, H3K27ac, and H3K27me3 – are commonly associated with gene regulation in the following ways:.
<4-3-42> H3K4me3 (Tri-methylation of lysine 4 on histone H3):
<4-3-43> This is often found near the transcription start site (TSS) of actively transcribed genes.
<4-3-44> It's associated with gene activation and is a hallmark of active promoters.
<4-3-45> H3K27ac (Acetylation of lysine 27 on histone H3):
<4-3-46> This modification is found both at promoters and enhancers of active genes.
<4-3-47> Acetylation generally opens up the chromatin structure, making it more accessible to transcriptional machinery and thus facilitating gene expression.
<4-3-48> H3K27me3 (Tri-methylation of lysine 27 on histone H3):

<4-3-49> This modification is associated with gene repression.
<4-3-50> It's often found across the body of genes that are silenced.
<4-3-51> It leads to a more compact chromatin structure, hindering the access of transcriptional machinery.
<4-3-52> Based on the three histone modifications:.
<4-3-53> Likely Expressed: EVL:
<4-3-54> There are peaks for H3K4me3 and H3K27ac near its promoter region, indicating active transcription.
<4-3-55> The absence of H3K27me3 further supports its active status.
<4-3-56> YY1:
<4-3-57> Similar to EVL, there are prominent peaks for both H3K4me3 and H3K27ac near its promoter region, indicating it's actively transcribed.
<4-3-58> WARS1:
<4-3-59> It also shows peaks for H3K4me3 and H3K27ac, suggesting active transcription.
<4-3-60> Not Likely Expressed or Lowly Expressed: DEGS2, SLC25A29, SLC25A47:
<4-3-61> While there might be minor peaks for H3K4me3 and H3K27ac, they aren't as prominent as those seen for YY1 or EVL. {There is no H3K27ac peak for the three genes.}
<4-3-62> Furthermore, regions of these genes display H3K27me3 peaks, hinting at potential repression or lower expression levels.
<4-3-63> WDR25:
<4-3-64> Shows minor peaks for H3K27ac but also has some regions with H3K27me3, suggesting it might be lowly expressed or in a bivalent state (a state in which genes are poised for either activation or repression). {There is no peak of H3K27me3. The H3K27ac peak is strong. The domain knowledge "bivalent state", which is correct by itself, is used to interpret a false claim on the presence of H3K27me3.}
<4-3-65> In summary, based on these modifications, EVL, YY1, and WARS1 are most likely actively expressed, while DEGS2, SLC25A29, SLC25A47, and WDR25 might be either lowly expressed or not expressed.

Q

4. Three groups of genomic regions are highlighted: "A"s in light blue, "I"s in pink, and "E"s in green. Identify the number of regions for each group.

<4-3-66> 4. Number of Genomic Regions in Each Group:.
<4-3-67> From the provided screenshot:.
<4-3-68> "A" regions (light blue): A1 A2 A3.
<4-3-69> Total: 3 regions.
<4-3-70> "I" regions (pink): I1 I2.
<4-3-71> Total: 2 regions.
<4-3-72> "E" regions (green): E1 E2 E3 E4.
<4-3-73> Total: 4 regions.
<4-3-74> To summarize: "A" group has 3 regions.
<4-3-75> "I" group has 2 regions.
<4-3-76> "E" group has 4 regions.

Q

5. For genomic regions in "A"s, what are their shared features in terms of positional relationship with genes and histone modification patterns? What is their potential function in transcription regulation?

<4-3-77> 5. Shared Features of "A" Regions:.
<4-3-78> Positional Relationship with Genes:
<4-3-79> From the provided screenshot, the "A" regions (A1, A2, and A3) have the following positional relationships with genes:.

<4-3-80> A1: Located within the gene body of EVL {A1 overlaps EVL promote, here prompter means TSS + its upstream region + its downstream region.}
<4-3-81> A2: Located close to the TSS (Transcription Start Site) of YY1.
<4-3-82> A3: Located within the gene body of WDR25 {A3 overlaps prompters of WARS and WDR25}
<4-3-83> Histone Modification Patterns:
<4-3-84> For the "A" regions, we can observe the following histone modification patterns:.
<4-3-85> All three "A" regions display prominent peaks for H3K4me3, which is a marker for active promoters.
<4-3-86> Similarly, there are noticeable peaks for H3K27ac in these regions, which indicates active transcriptional regions (either active promoters or enhancers).
<4-3-87> There's an absence of H3K27me3 peaks in these regions, indicating that these regions are not repressed.
<4-3-88> Potential Function in Transcription Regulation:
<4-3-89> Given their positional relationships with genes and the observed histone modification patterns, the potential functions of "A" regions in transcription regulation are:.
<4-3-90> A1:
<4-3-91> As it's located within the gene body of EVL, it could represent an intragenic enhancer or another regulatory element modulating the expression of EVL. {A1 overlaps EVL promote. The domain knowledge "intragenic enhancer", which by itself is correct, is used to interpret a false observation "within the gene body of EVL".}
<4-3-92> A2:
<4-3-93> Being close to the TSS of YY1 and showing both H3K4me3 and H3K27ac marks, this suggests that A2 could be the primary promoter region for YY1, playing a crucial role in its active transcription.
<4-3-94> A3:
<4-3-95> Located within the gene body of WDR25, it might also represent an intragenic enhancer or a regulatory element for WDR25. {A3 overlaps prompters of WARS and WDR25. The domain knowledge "intragenic enhancer", which by itself is correct, is used to interpret a false observation "within the gene body of WDR25"}
<4-3-96> In general, the "A" regions appear to be associated with active transcriptional regulation, either as promoters or enhancers, given their histone modification patterns and their proximity to the genes.

6. How about genomic regions in "I"s? What is their potential function in transcription regulation?

<4-3-97> 6. Genomic Regions in "I"s:.
<4-3-98> From the provided screenshot, the "I" regions (I1 and I2) exhibit the following features:.
<4-3-99> Histone Modification Patterns: For the "I" regions:.
<4-3-100> There's a noticeable absence of the H3K4me3 peak, which is typically associated with active promoters. {The H3K4me3 peak is weak, not absent.}
<4-3-101> Similarly, there's no prominent peak for H3K27ac, which marks active transcriptional regions.
<4-3-102> Notably, there's a clear presence of H3K27me3 across these regions, which is a marker associated with gene repression.
<4-3-103> Potential Function in Transcription Regulation: Given the observed histone modification patterns:.
<4-3-104> The presence of H3K27me3 suggests that these "I" regions are associated with transcriptional repression or silencing.
<4-3-105> They could represent repressed chromatin domains or regions where genes are in a "poised" state, ready to be activated under specific conditions but currently inactive.
<4-3-106> Alternatively, they might be involved in maintaining genes in a silent state, preventing their transcription in the particular cell type or under the specific conditions sampled.
<4-3-107> In summary, the "I" regions, given their histone modification patterns, appear to be associated with repressive transcriptional regulation, ensuring that certain genes or genomic regions remain inactive or silenced.

Q

7. How about genomic regions in “E”s? What is their potential function in transcription regulation?

<4-3-108> 7. Genomic Regions in "E"s:.
<4-3-109> From the provided screenshot, the "E" regions (E1, E2, E3, and E4) exhibit the following features:.
<4-3-110> Histone Modification Patterns: For the "E" regions:.
<4-3-111> They display noticeable peaks for H3K27ac, which is typically associated with active enhancer regions.
<4-3-112> The presence of H3K27ac indicates that these regions are transcriptionally active.
<4-3-113> There's an absence of the repressive marker H3K27me3, indicating these regions are not in a repressed state.
<4-3-114> Unlike "A" regions, the "E" regions do not consistently show strong H3K4me3 peaks, which is more associated with active promoters.
<4-3-115> Potential Function in Transcription Regulation: Given the observed histone modification patterns:.
<4-3-116> The presence of H3K27ac, combined with the absence of H3K27me3, suggests that these "E" regions are likely functioning as active enhancers.
<4-3-117> Enhancers are DNA sequences that can up-regulate the transcription of target genes.
<4-3-118> They can be located far from the genes they regulate and can act in either orientation.
<4-3-119> Enhancers can play a role in cell-type-specific gene expression, ensuring that genes are expressed at the right levels in the right contexts.
<4-3-120> The "E" regions might be binding sites for transcription factors and other regulatory proteins that help recruit the transcriptional machinery to the target gene's promoter.
<4-3-121> In summary, the "E" regions, given their histone modification patterns, appear to be associated with enhancer-mediated transcriptional activation.
<4-3-122> They might be involved in up-regulating the expression of nearby or even distant genes in the particular cell type or under the specific conditions sampled.

Q

8. On the heatmap for chromatin-chromatin interactions, there are several circles each connected with dashed lines. Explain what the circle means and how the dashed lines are connected to the circle. What are the common features of the circles in terms of indicated genomic region(s)?

<4-3-123> 8. Chromatin-Chromatin Interactions on the Heatmap:.
<4-3-124> The heatmap represents chromatin-chromatin interaction data, which visualizes the physical interactions between different regions of the genome, often derived from techniques such as Hi-C or ChIA-PET.
<4-3-125> Circles:
<4-3-126> These circles on the heatmap highlight significant interactions between two genomic regions.
<4-3-127> The position of the circle on the heatmap represents the two interacting regions.
<4-3-128> For instance, if there's a circle at the intersection of the E1 and A2 lines, it means there's a significant chromatin interaction between the E1 and A2 regions.
<4-3-129> Dashed Lines:
<4-3-130> The dashed lines connect the circles on the heatmap to their corresponding genomic regions on the top and side axes of the heatmap. {Dashed lines points to two genomic regions indicated from the top axis.}
<4-3-131> They serve as a visual aid to trace back the interactions to the exact genomic locations.
<4-3-132> For example, a circle connected by two dashed lines to the E2 region and the A2 region indicates that these two genomic regions are interacting with each other.
<4-3-133> Common Features of the Circles in Terms of Indicated Genomic Region(s):.
<4-3-134> From the provided screenshot:.
<4-3-135> Many of the circles, or interaction points, are connected to the "A" regions, specifically A2, which is near the YY1 gene.
<4-3-136> This suggests that A2, which is likely the promoter region of YY1, has multiple interactions with other genomic

regions.
<4-3-137> A considerable number of circles also connect the "E" regions to the A2 region.
<4-3-138> This indicates that these "E" regions, which are potential enhancer regions, might be interacting with the promoter region of YY1 (A2) to regulate its transcription.
<4-3-139> It is also worth noting that there are interactions between different "E" regions themselves, suggesting possible cooperation or co-localization in the nucleus.
<4-3-140> In essence, the common feature of these circles is that they often involve interactions of potential enhancer regions ("E"s) with the promoter region of YY1 (A2), indicating a complex regulatory landscape for the YY1 gene, where multiple enhancers might be coming into proximity with its promoter to modulate its expression.

Q

9. There are two groups of circles by color. Identify the number of circles for each group.

<4-3-141> 9. Number of Circles in Each Color Group:.
<4-3-142> From the provided screenshot:.
<4-3-143> Purple Circles: {There is no purple circle.}
<4-3-144> I count 3 purple circles on the heatmap. {There is no purple circle.}
<4-3-145> Blue Circles:
<4-3-146> I count 2 blue circles on the heatmap {3 blue circles.}
<4-3-147> To summarize: There are 3 purple circles. {There is no purple circle.}
<4-3-148> There are 2 blue circles. {3 blue circles.}

Q

10. Focus on the circles in blue, and identify their indicated interacting genomic regions.

<4-3-149> 10. Interacting Genomic Regions Indicated by Blue Circles:.
<4-3-150> From the provided screenshot, the blue circles indicate the following interacting genomic regions:.
<4-3-151> The first blue circle is connecting the "E2" region with the "A2" region. {The interaction is for A1::A2.}
<4-3-152> This suggests a chromatin interaction between the E2 and A2 genomic regions. {The interaction is for A1::A2.}
<4-3-153> The second blue circle is connecting the "E3" region with the "A2" region. {The interaction is for I1::A2.}
<4-3-154> This indicates an interaction between the E3 and A2 genomic regions. {The interaction is for I1::A2.}
<4-3-155> To summarize: The first blue circle indicates an interaction between E2 and A2. {The interaction is for A1::A2.}
<4-3-156> The second blue circle indicates an interaction between E3 and A2. {The interaction is for I1::A2.}

Q

11. Focus on the circles in green, and identify their indicated interacting genomic regions.

<4-3-157> I apologize for the oversight in my previous responses, but from the provided screenshot, there aren't any green circles. {There 3 green circles}
<4-3-158> You mentioned earlier there were purple and blue circles. {Blue not purple.}
<4-3-159> I've already addressed the blue circles.
<4-3-160> Would you like me to focus on the purple circles instead and identify their indicated interacting genomic regions? {There is no purple circle.}

Q

12. Are there any specific suggestions to improve the data presentation of the figure?

<4-3-161> 12. Suggestions to Improve Data Presentation:.
<4-3-162> While the figure provides a comprehensive view of multiple data types, there are always ways to enhance clarity and interpretation.
<4-3-163> Here are some suggestions:.
<4-3-164> 1. Enhanced Color Contrast:
<4-3-165> Ensure there's sufficient color contrast between different elements (like the "A", "E", and "I" regions) to help those with color vision deficiencies.
<4-3-166> 2. Legends and Annotations:
<4-3-167> Including a clear legend that defines each color, symbol, or pattern will help the viewer understand the data more effectively.
<4-3-168> For instance, while the heatmap shows interactions, a simple legend explaining the color intensity's significance (darker color = stronger interaction) might be beneficial.
<4-3-169> 3. Simplify Visual Noise:
<4-3-170> The dashed lines connecting the circles on the heatmap to their corresponding genomic regions are helpful, but if there are too many, it can make the figure look cluttered.
<4-3-171> Using straight lines or varying line styles (dotted, dashed, solid) for different circle groups could help differentiate them.
<4-3-172> 4. Scale Bars: Include a scale bar or markers to provide a sense of the genomic distance covered by the figure.
<4-3-173> 5. Histogram Y-Axis Labels:
<4-3-174> For the RNA-Seq and ChIP-seq plots, labeling the y-axis with the read count or signal intensity will provide context to the peaks' relative heights.
<4-3-175> 6. Zoomed-In Views:
<4-3-176> For regions with complex interactions or multiple overlapping elements, a zoomed-in view or an inset can help provide clarity.
<4-3-177> 7. Interactive Features:
<4-3-178> If this figure is presented online, adding interactive features like tooltips, zoom functions, and clickable elements can enhance user experience.
<4-3-179> 8. Additional Descriptive Text:
<4-3-180> Brief textual descriptions or annotations near critical regions, especially highlighting significant findings or unusual patterns, can guide the viewer's attention.
<4-3-181> 9.
<4-3-182> Group Related Data:
<4-3-183> Group related data tracks together for better comparison.
<4-3-184> For instance, having all histone modifications grouped can make it easier to compare and contrast their patterns across the genomic region.
<4-3-185> 10. 3D Visualization:
<4-3-186> Given that chromatin interactions are inherently 3D, integrating 3D visualizations (if feasible) can give a more intuitive representation of the interactions.
<4-3-187> These suggestions aim to enhance clarity, reduce potential ambiguity, and offer a more intuitive understanding of the data presented.

Q

13. Draft a title and a legend for this figure based on our previous discussion. Make sure to include details.

<4-3-188> 13.
<4-3-189> Title and Legend for the Figure:.
<4-3-190> ---.
<4-3-191> Title: "Integrated Epigenomic Landscape Reveals Transcriptional Regulation of YY1 in GM12878 Cells".
<4-3-192> ---.

<4-3-193> Legend:.
<4-3-194> RefSeq Gene Track:
<4-3-195> Displayed genes include EVL, DEGS2, YY1, SLC25A29, SLC25A47, WARS1, and WDR25.
<4-3-196> Arrows indicate the direction of transcription.
<4-3-197> RNA-Seq (+/-): Represents RNA sequencing reads aligned to the positive (+) and negative (-) DNA strands.
<4-3-198> Peaks signify gene expression levels.
<4-3-199> H3K4me3: ChIP-seq track indicating tri-methylation of lysine 4 on histone H3, a marker for active promoters.
<4-3-200> H3K27ac: ChIP-seq track for acetylation of lysine 27 on histone H3, signifying active transcriptional regions (promoters or enhancers).
<4-3-201> H3K27me3: ChIP-seq track showing tri-methylation of lysine 27 on histone H3, associated with gene repression.
<4-3-202> Highlighted Regions:
<4-3-203> Light Blue (A): Regions associated with gene bodies or near transcription start sites, marked by active transcriptional histone modifications.
<4-3-204> Pink (I): Regions marked predominantly by the repressive H3K27me3 modification.
<4-3-205> Green (E): Potential enhancer regions, marked by H3K27ac.
<4-3-206> Heatmap (Chromatin-Chromatin Interactions): Blue Circles:
<4-3-207> Indicate significant chromatin interactions between regions such as E2-A2 and E3-A2. {E2-A2 and E3-A2 are wrong examples. Corrects are: A2-A1, A2-I1, and A2-A3}
<4-3-208> Purple Circles: Highlight other noteworthy chromatin-chromatin interactions. {There is no purple circle.}
<4-3-209> Dashed Lines: Connect significant interaction points on the heatmap to their corresponding genomic regions, visually indicating interacting pairs.
<4-3-210> ---.
<4-3-211> This title and legend provide a comprehensive overview of the figure and its elements based on the discussion and information provided.

Q

14. Draft a paragraph to describe the findings from our previous discussion on transcription regulation of YY1. This is one paragraph for the result part of a manuscript. 150 words or less.

<4-3-212> 14. Descriptive Paragraph on Transcription Regulation of YY1:. ---.
<4-3-213> In our comprehensive epigenomic analysis of GM12878 cells, we observed intricate transcriptional regulation mechanisms governing YY1 expression.
<4-3-214> The promoter region of YY1, demarcated as A2, exhibited prominent H3K4me3 and H3K27ac peaks, indicative of active transcription.
<4-3-215> This region displayed significant chromatin interactions with potential enhancer regions, specifically E2 and E3, as evidenced by the chromatin interaction heatmap.
<4-3-216> These enhancers were marked by H3K27ac, further substantiating their active regulatory roles.
<4-3-217> Notably, regions I1 and I2, characterized by the repressive H3K27me3 mark, suggested a landscape poised for transcriptional modulation.
<4-3-218> Such a complex interplay of promoter-enhancer interactions and chromatin modifications underscores the intricate regulatory network orchestrating YY1 expression in GM12878 cells.
